# Structural Insights of Humins/Epoxidized Linseed Oil/ Hardener Terpolymerization

**DOI:** 10.3390/polym12071583

**Published:** 2020-07-16

**Authors:** Erol Licsandru, Marc Gaysinski, Alice Mija

**Affiliations:** Institut de Chimie de Nice, Université Côte d’Azur, UMR CNRS 7272, Cedex 02, 06108 Nice, France; erol.licsandru@unice.fr (E.L.); Marc.GAYSINSKI@univ-cotedazur.fr (M.G.)

**Keywords:** renewable resources, biorefinery by-products, humins, copolymerization, epoxidized vegetal oils, reaction mechanism, ^1^H, ^13^C NMR

## Abstract

Bio-based thermosetting resins were synthesized from a ternary composition: humins; epoxidized linseed oil (ELO); and an industrial hardener, Capcure3-800 (CAP). Humins are in a focused attention in the last years, as biorefinery by-product, therefore its valorization through materials design is very important. Here we present a structural study of terpolymerization of humins/ ELO/CAP. The reactivity of these systems was highlighted by in situ FT-IR and ^1^H and ^13^C NMR. The integration of humins in thermosetting resins gives alternatives to new feedstocks for future bio-based materials.

## 1. Introduction

The variance in oil prices and the inherent instability of oil producing zones lead to an increased pressure in the development of alternative materials’ feedstock. Bio-refineries are a promising step towards the future [1,2,3] by conversion of bio-polymers such as cellulose and hemicellulose to simple monomeric building blocks such as hydroxymethylfurfural (HMF), methoxymethylfurfural (MMF), and levulinic acid (LA). The process requires the catalytical conversion of simple carbohydrates towards the aforementioned monomers [4]. These bio-monomers can be used in the synthesis of bio-plastics.

**Figure 1:**
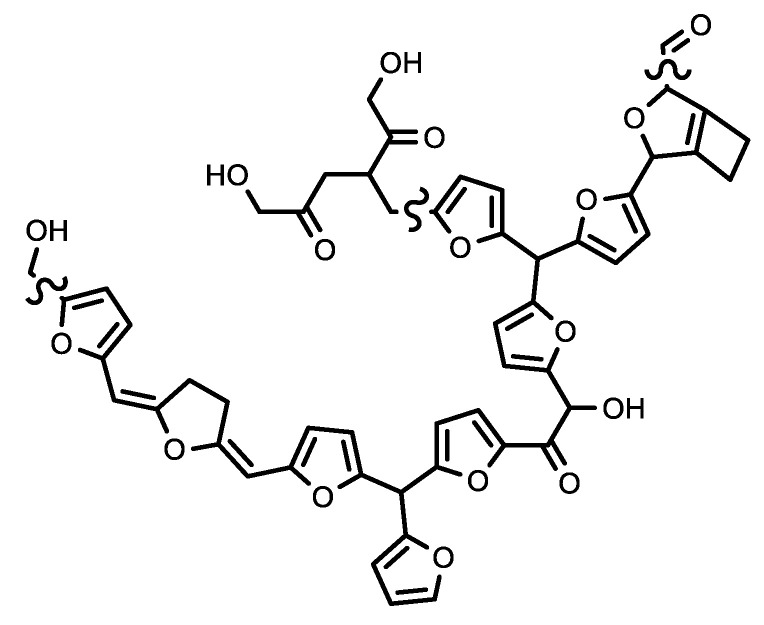
Proposed fragment of a humins [5]. Polyfuranic moieties observed. Multiple types of reactive oxygen atoms are present (acids, alcohol, ketones, etc.).

In this work, we focus on the synthesis of thermosetting resins starting from renewable products for the eco-design of a novel family of materials. The design of the bio-based thermosets has been done by choosing three main components: the humins (HU), a by-product from bio-refineries; the epoxidized linseed oil (ELO), an epoxy derivative of natural poly unsaturated oils; and Capcure 3-800 (CAP), an industrial hardener for epoxy resins. The ternary polymerization strategy was applied with the aim to combine two biobased and antagonist (macro)monomers: the humins and ELO. In this way, the humins contribute the polyfuranic branched chains, giving rigidity to the network while the aliphatic chains of ELO give elasticity and a ductile character. Due to their low miscibility and reactivity, the Capcure was used both as hardener and as compatibilizer. The rationale of this work was the valorization of humins, as biorefinery by-product. For this reason, the design of formulations, of reactivity, etc., were realized considering the humins as the main component, with a proposed structure presented in Figure 1 [5].

Humins are materials with complex composition and behaviour, depending on biorefinery process. The elemental composition was determined by Mulder in 1840 [6] as constituted by ~64–65% C, ~5% H, and ~31–32% O. Other studies have placed the composition between 58–65.5% C and 4.5% H, depending on the starting feedstock [7,8]. Humins are mixtures of macromolecules containing fused and condensed cycles, generally derived from furan (60%) and aliphatic linkers (20%) [9]. Given their limited solubility, alternative strategies have been devised in order to characterize these structures. Titirici and Baccile [10,11] investigated the structures generated by solid state ^13^C NMR as well as through ^13^C labelling, based on reaction conditions and feedstock. Electron microscopy studies, SEM and TEM, have revealed structures made up of spheres with diameters between 0.5 and 5 μm [12,13,14,15]. The Raman studies [14] confirmed the presence of reactive oxygen atoms in the humins macrostructure. The presence of reactive oxygen species is a structural key allowing to transform the humins into thermosetting resins. Through specific derivatization with a fluorinated hydrazine derivative ((trifluoromethyl)phenylhydrazine), the total amount of carbonyl functions present in industrial humins was evaluated by Constant et al. using fluorine NMR to 6.6 wt % [16]. Fluorine NMR presents the advantage of providing a distinct response since the proton and carbon spectra would be too complex for quantitative and qualitative analyses. The assay provided quantitative data on the reactive oxygen species found in humins, also revealing a richness of ketones compared to alcohol and acid functions. This information is particularly useful in determining the chemical reactivity of the humins. Recently, another study concerning humins solubility correlated with IR characterization has been published [17]. Successive attempts of solubilizing/extracting of various fractions from the raw humins were partially successful. Indeed, some smaller and more soluble molecules were isolated from the mixture; however, the IR spectra suffered small modifications. This publication provides a very astute understanding over the general spectral imprint of humins. The study also revealed that contrary to previous proposals the molecular weight of the components might be significantly lower, and the oligomers might be the most prevalent species.

**Figure 2:**
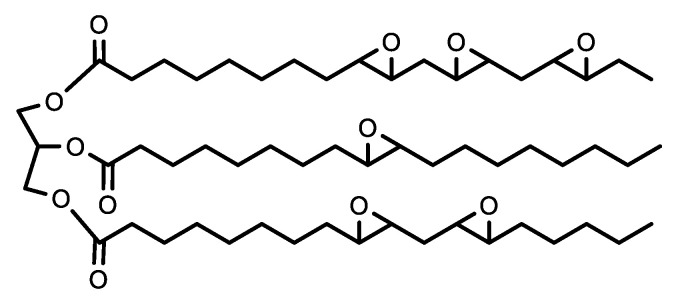
Epoxidized linseed oil (ELO). The triglyceride presents a mixture of oleic, linoleic, and linolenic acid residues. Average number of oxirane cycles per triglyceride = 5.5.

Bio-sourced oils are a renewable feedstock with multiple applications as bio-diesel and in last decades as precursors of polymers [18,19,20,21]. In this case, the polyunsaturated triglycerides allow a facile transition towards epoxidized oils, via direct oxidation of the double bonds or by enzymatic route [22,23]. The epoxidized oils can then be used in various formulations of lubricants [24,25] polyurethanes, [18,26,27] and thermosetting polymers [28,29,30,31]. To develop thermosetting resins, the most commonly used oils are the epoxidized soybean oil (ESO) and the epoxidized linseed oil (ELO) with 3.5 and 5.5 oxirane rings respectively per triglyceride. Both in the case of ESO [24,32,33,34,35] and ELO [29,36,37,38,39], numerous applications were reported. In this study, we used the epoxidized linseed oil (ELO) with the structure given in Figure 2.

**Figure 3:**
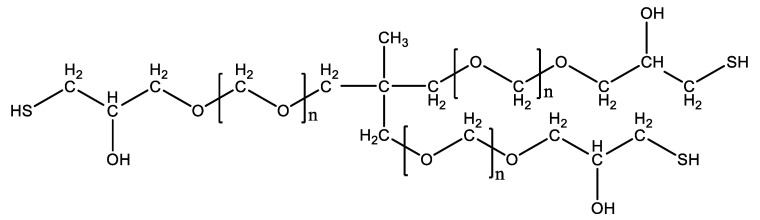
Capcure 3-800 structure.

To promote the crosslinking reaction the Capcure 3-800 (CAP) was used. Its backbone contains polypropoxy segments that improve the compatibility between the polar structure of humins and the nonpolar structure of ELO. The ends of the CAP backbone have both thiol and hydroxyl moieties, opening several possible reactions with ELO and HU components (Figure 3).

In a previous study, we proved for the first time that we can prepare bioresins by incorporating up to 60% weight of humins in formulations with ELO and CAP leading to resins with a bio-sourced content up to 70% [40]. Four formulations were prepared and tested in this study, containing from 30% to 60% HU, and the thermomechanical properties of obtained resins characterized, showing tan δ values from ~10 to 60 °C and *T*_d5%_ values from 250 to 300 °C. The hardness of the resulted materials varied between 64A and 49D, characteristic of elastomers to rigid thermosets. Starting from these preliminary results on formulation design and on thermomechanical properties of produced thermosets, the aim of this study is to gain knowledge on chemical mechanism and structural aspects of HU/ELO/CAP terpolymerization. The formulation with 50% HU was selected for this study, with the aim to keep a maximal humins ratio and also a representative proportion between the three comonomers. To go further within the terpolymerization mechanism, we studied in a first stage the HU/ELO, HU/CAP, and ELO/CAP binary copolymerization by in situ FTIR spectroscopy and by reacting the formulations during selected time and analyzing them by liquid NMR. Thereafter, the terpolymerization formulation was investigated by rheometry and by the same protocols of FTIR and NMR analyses.

## 2. Materials and Methods

### 2.1. Materials

The epoxidized linseed oil (ELO) was obtained from Valtris Ltd. as a viscous-liquid at room temperature, having a viscosity of about 1200 mPa∙s. ELO has a molecular weight of about 980 g∙mol^−1^ and contains about 5.5 epoxy groups, on average, per triglyceride.

Humins (HU) were provided by Avantium Chemicals as produced at the Avantium pilot plant in Geleen (Netherlands). These humins oligomers have molecular masses that generally range from 270 to 650 g∙mol^−1^ [41].

Capcure^®^ 3-800 (CAP) is a mercaptan-terminated product used as a liquid curing agent with unique rapid-cure characteristics for epoxy resins at ambient temperatures provided by Gabriel Performance Products.

### 2.2. Samples Preparation

To prepare a resin with the maximum amount of humins we used in this study a ratio HU/ ELO/CAP of 50/37.5/12.5 (wt %). The general protocol for the preparation of resins containing humins, Capcure, and ELO is as follows. ELO and CAP were mixed together at room temperature in the desired proportion. Then, the mixture containing ELO and CAP was mixed with the humins preheated at 80 °C. The formulation was thoroughly mixed until a homogeneous phase was obtained. Then, the resin mixture was poured into molds at 80 °C. The curing process takes 4 h followed by post-curing 130 °C for another 3 h.

The samples for rheometry were prepared as follows. The reaction mixture was made following the presented protocol and placed in the rheometer at 30 °C. This mixture was heated from 50 to 200 °C at 0.2 °C∙min^−1^.

The IR formulations were prepared in the above stated fashion. Then, the reactions were conducted directly on the heating plate of the instrument, at 80 °C for 4 h then at 130 °C for 4 h (post curing).

In the case of the NMR samples this protocol was adapted as follows. An amount of humins was introduced in the tube. The corresponding amounts of CAP and ELO were calculated and premixed. The humins were then dissolved in deuterated DMSO. The sample was well homogenized prior to add the other components. After, the sample was again homogenized using a vortex and ultrasounds. All samples were made using a proportion of 10% (*w/w*) resin precursors to the DMSO. All samples were kept, during the lengthy experiment, in an oven at a constant temperature of 80 °C, in order to respect the standard reaction conditions. They were removed only during the NMR experiments and the time was calculated accordingly.

### 2.3. Experimental Techniques

Rheometry measurements were performed using an Anton Paar MCR–302 rheometer using disposable plate–plate geometries (25 mm diameter and 1 mm gap). The measurements were carried out at a scan rate of 0.2 °C∙min^−1^ over a temperature range 50 °C to 200 °C. Complex viscosity, storage modulus (G’) and loss modulus (G”) were measured by oscillatory shear experiments with an angular frequency of 10 rad∙s^−1^ and a deformation of 0.2%. The temperature of crossover of the values of storage (G’) and loss (G”) moduli was considered as the temperature of gelation (*T*_gel_).

FT-IR spectroscopy was used to investigate the structural changes occurred during the curing. A Thermo Scientific Nicolet iS50 FT-IR spectrophotometer was employed in attenuated total reflectance (ATR) mode with a PIKE GladiATR diamond crystal. The spectrum of air was recorded as background, all the measurements being provided at 32 scans with a resolution of 4 cm^−1^. The starting components HU, ELO, CAP, the HU/ELO/CAP mixtures and thermoset material obtained following the protocol given in Section 2.2 (curing at 80 °C during 4 h and post curing at 130 °C during 3 h) have been analyzed and compared.

High resolution NMR spectra were recorded in DMSO d6, at 25 °C (temperature control BCU 6.0, BVT 3000), on a Bruker Avance DRX 500 spectrometer operating at 500.13 MHz for ^1^H and 125.76 MHz for ^13^C. 1D and 2D NMR spectra were run with a direct probe head: 5 mm PADUL ^13^C-^1^H Z-GRD. Spectrum calibration was performed by using DMSO d6 signal as internal reference (2.5 ppm for ^1^H NMR, 39.5 ppm for ^13^C NMR). Chemical shifts (δ) are expressed in parts per million (ppm) and coupling constants (J) in Hertz. All NMR experiments were carried out using pulse sequences supplied by the spectrometer manufacturer (Bruker–TOPSPIN 2.1).

## 3. Results and Interpretations

### 3.1. Hypothesis on HU/ELO/CAP Copolymerization Mechanism

**Scheme 1:**
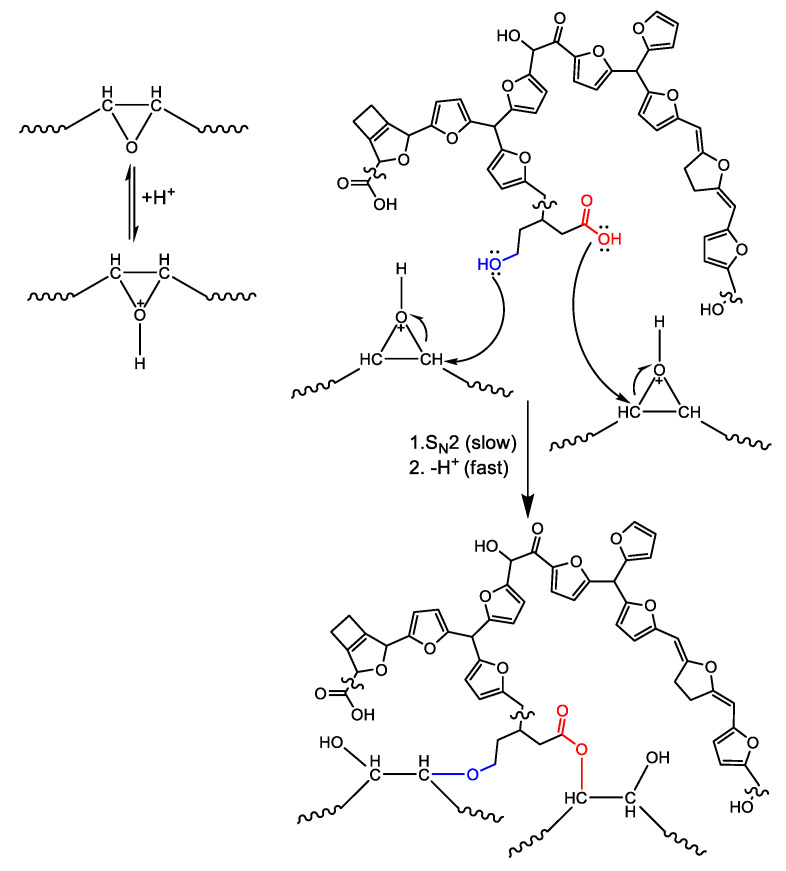
Proposed reaction mechanism for the interaction between humins and epoxides function from ELO.

As humins are complex chemical compounds, the approach considered in this study is contrary to the standard chemical approach. Instead of targeting specific reaction by interaction of specific species or function, we propose here the use of unselective pathways reactions, by multiple chemical connectivity implying multiple moieties. As previously mentioned, the humins present a polydisperse nature, containing reactive functions comprising alcohols, acids, aldehydes, and ketones [5]. From the standpoint of an epoxide, such as ELO, of interest are the alcohol and acid functionalities. Given the fact that both alcohols and acids are weak nucleophiles the first step of the reaction is the protonation of the oxirane ring to the oxonium ion. A nucleophilic attack is performed by both alcohol and acid functionalities on the carbon of the oxonium ion. In this step, the oxygen carbon bond is broken and the ring is opened. The products, an ether in the case of alcohols and an ester in the case of acids are formed by the elimination of a proton. This mechanism is presented in Scheme 1.

**Scheme 2:**
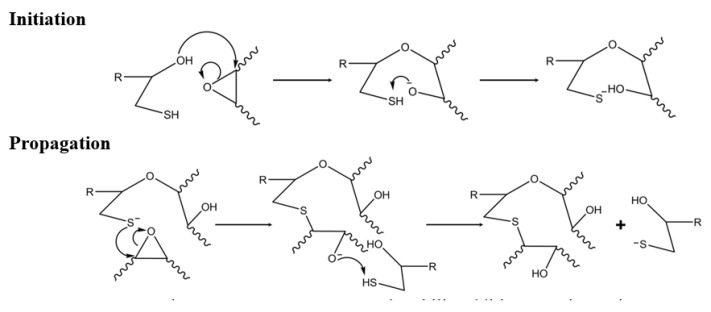
Capcure–ELO nucleophilic addition to oxirane ring.

From the standpoint of Capcure and ELO the reaction follows a standard initiation/propagation polymerization mechanism. Generally, thiol-based epoxy curing agents react under basic conditions and require the presence of a tertiary amine as initiator. The tertiary amine removes the proton from the thiol and the resulting sulfur anion attacks the oxirane moiety through nucleophilic addition. In the particular case of Capcure, the amine initiator is not required, the hydroxyl moiety bonded to the α-carbon atom from the thiol carbon atom acts as the initiator. The hydroxyl function performs a nucleophilic attack on the epoxy ring. The oxygen from the oxirane moiety removes then the proton from the thiol, thus ending the initiation phase. The resulting thiol anion is a strong nucleophile and readily attacks the epoxy moieties. Just like in the case of the amine-initiated process, the propagation is achieved through the removal of protons from thiol containing molecules by the resulting negatively charged oxygen atom (Scheme 2).

Our experiments showed that in the specified conditions no measurable chemical reaction can be perceived between Capcure and humins. However, this does not exclude the possibility of reactions between intermediaries of the Capcure-ELO reaction and humins. The poor miscibility between humins and ELO is solved by the Capcure playing also the role of compatibilizer agent. The polypropoxylated chains belonging to Capcure act as a phase transition agent, while presenting a good reactivity towards ELO. The intermediary opened ring epoxide may readily react with the alcohol and acid moieties from humins’ structure.

### 3.2. Study of HU/ELO/CAP Reactivity and Gelation by Rheometry

**Figure 4:**
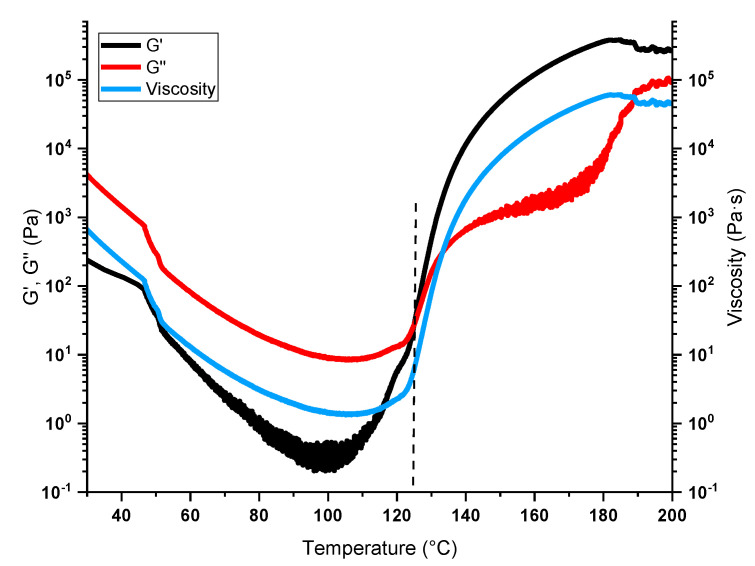
Rheometry study of HU/ELO/CAP during copolymerization. Evolution of moduli and viscosity.

As we can observe in Figure 4 by the results of the rheometry analysis the reaction mixture is characterized by high values of G’ and G” moduli, and also by a high value of the viscosity at around 750 Pa∙s. Increasing the temperature, the viscosity drops drastically, with three decades, reaching the value of ~1.4 Pa∙s at temperature above 100 °C. The mixture starts to react, when heated at 0.2 °C/min^−1^, at around 110 °C. The hardening of resin, given by the gel point of the system, occurs at around 125 °C, being taken as crossover between G’ and G’’. We can observe by the shape of the curves and by the fast increase of the viscosity that the reaction occurs fast, being completed at around 180 °C.

### 3.3. Infrared Spectroscopies Study on HU/ELO/CAP Copolymerization System

**Figure 5:**
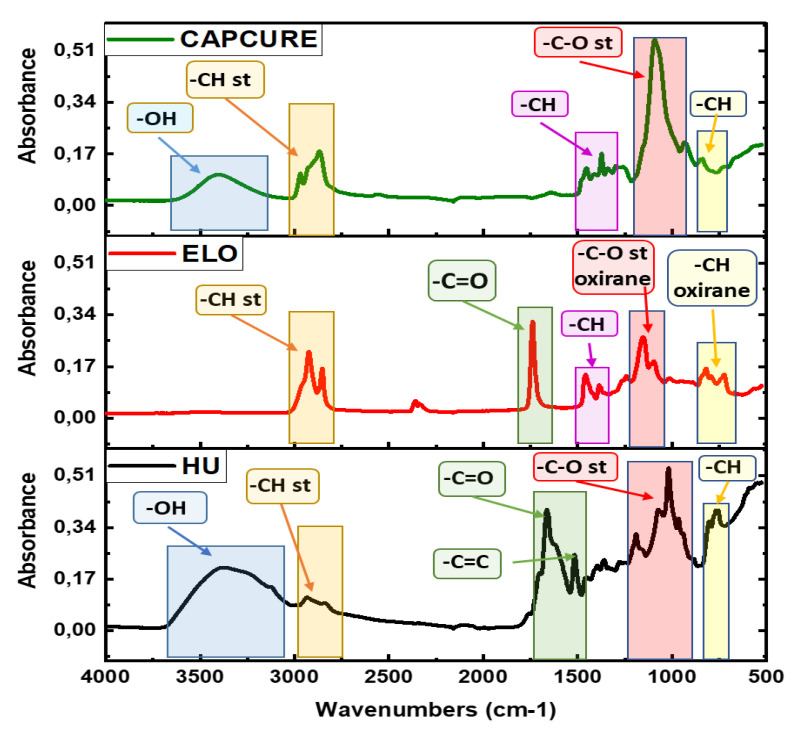
FTIR spectra of the starting materials.

In order to validate the hypothesis presented for the copolymerization mechanism, infrared spectroscopy was employed under the exact copolymerization conditions. The spectra of the starting materials, humins, Capcure, and ELO are presented in Figure 5.

**Figure 6:**
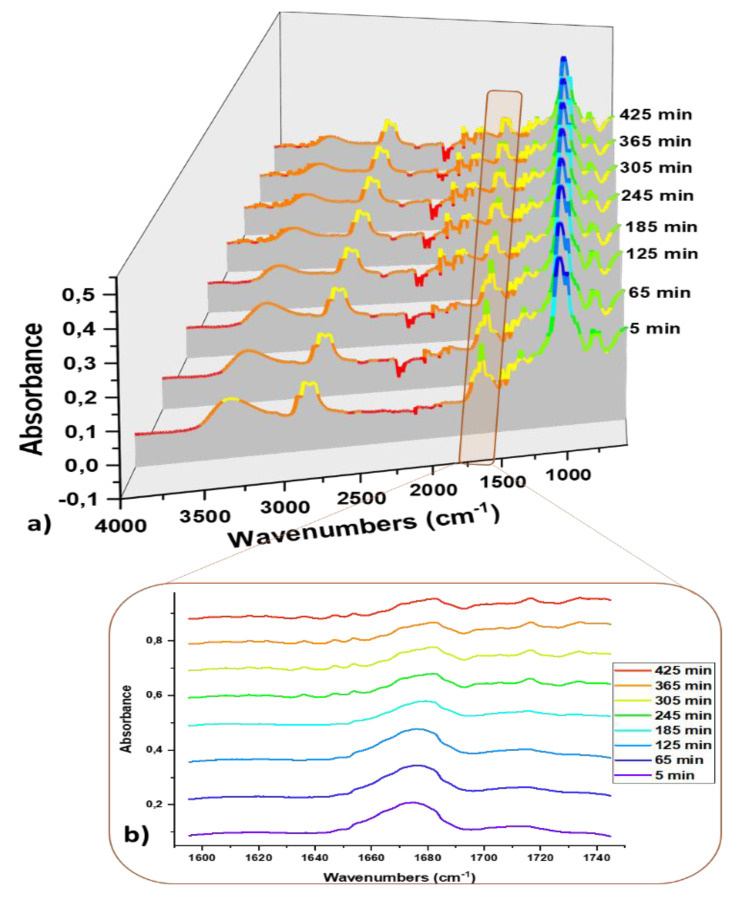
(**a**) and (**b**): FTIR spectra presenting the evolution of the HU/ELO/CAP copolymerization reaction over time (**a**) and detail of the carbonyl area (**b**).

The reaction was studied in situ at a temperature program mirroring the experimental conditions as described in Section 2.2. Spectra were collected at fixed intervals of time to monitor the modifications in the signal. These results are presented in Figure 6a and summarized in Table S1.

By analyzing the IR spectra three conclusions can be drawn. Firstly, the copolymerization took place, fact sustained by the multiple modifications in the spectra. Secondly, the chemical structures of the starting materials present multiple bands representing the same type of chemical bonds with a large amount of overlap between the bands, with many of them being present in the starting materials, in the intermediaries, and in the final product. Therefore, one can draw only qualitative information from the FTIR experiment. Thirdly, the most relevant modifications in the IR spectra are found in the carbonyl area, between 1720 and 1660 cm^−1^. While the attribution of individual bands is impossible due to the multitude of carbonyl moieties, found particularly in the humins, a trend is displayed. In the initial spectra, an envelope of all carbonyl moieties is observed (Figure 6b), representing an average of all the bands. However, as the chemical reaction takes place, several smaller signals become more and more apparent. These bands correspond to specific aldehyde, ester, and α, β- unsaturated ketones. This segregation of signals is a strong indicator of the passage from the structure specific to humins, towards a more organized structure of a resin. The limits of the sensibility of this method being clear, a more sensitive method—NMR—was employed to provide supplementary structural data.

### 3.4. NMR Spectroscopy Studies

#### 3.4.1. Characterization of the Components of Copolymerization System ELO

At 25 °C, 1D and 2D NMR analyses allowed us to identify the structure of ELO (Figure S1a–f). The obtained results are summarized in Table 1 and are in good agreement with previous reported data [42].

Briefly, the resonance frequency of CH_2_–e_E_ were attributed by corroborating the presence of correlation in COSY experiment between CH_2_–f_E_ (δ_H_ = 2.21–2.31 ppm) and CH_2_–e_E_ (δ_H_ = 1.47–1.55 ppm). CH_2_- h_E_ are more deshielded due to the presence of two epoxides groups in α and α’ positions. This fact is confirmed in COSY and TOCSY experiments by the presence of correlations between methylene proton h_E_ (δ_H_ between 1.56 ppm and 1.88 ppm) and CH–g_E_ proton (δ_H_ between 2.84 ppm and 3.12 ppm). 2D NMR also allow us to detect the presence of methyne resonance peak (δ_H_ = 5.54 ppm, δ_C_ = 126.57 ppm) indicating the presence of some residual double bonds in ELO sample [43].

#### 3.4.2. Capcure

Capcure is a reaction product of pentaerythritol, propoxylated, and 1-chloro-2,3-epoxypropane with hydrogen sulphide. At 25°C, 1D and 2D NMR experiments (Figure S2a–f) permitted us to identify the different elements of this compound and in particular his reactive part: 3-mercapto-2 propanoyl residue. The results are presented in Table 2. Briefly, we can see in TOCSY experiment a correlation between the thiol proton SH-1_c_ (δ_H_ = 2.08 ppm) and the methylene proton 2_c_ in α position (δ_H_ = 2.47 ppm, δ_H_ = 2.61 ppm), the CH-3_c_ proton in β position (δ_H_ = 3.60 ppm), the hydroxyl proton 4_c_ (δ_H_ = 4.91 ppm) and the methylene proton 5_c_ in γ position (δ_H_ = 3.58 ppm). NMR experiments also show the presence of other resonance frequencies together with those of the main product. Different conformers of the main product in solution, non-functionalized compounds, by-product, or rearrangement product are hard to distinguish. In order to know how many reactive groups are per molecule we integrated the signals of hydrogen sulphur (δ_H_ = 2.09 ppm – triplet) and all the ^1^H signals corresponding to methyl proton (δ_H_ = 1.042 ppm – m). The ratio gives a result between 1/5 (big doublet centered at 1.04 ppm) and 1/7 (all the ^1^H signal corresponding to methyl proton). Normally, if the entire site is functionalized, we should have a ratio of 4/15. This result indicates that we have approximatively a functionalization ranging between 53% and 75% that means around 2 and 3 reactive groups (3-mercapto-2 propanoyl residue) per molecule.

#### 3.4.3. Humins

**Figure 7:**
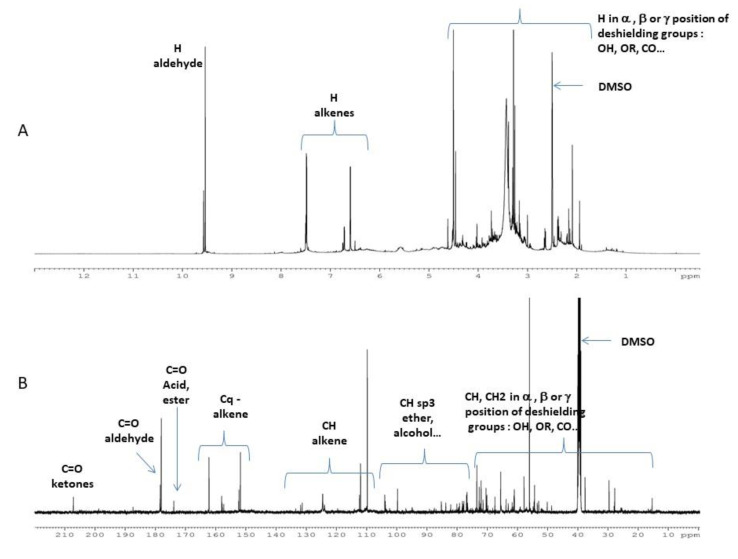
(**A**) ^1^H NMR Spectrum of HU (DMSO d6); (**B**) ^13^C NMR Spectrum of HU (DMSO d6).

Humins are complex by product of biorefining process aimed at the valorization of lignocellulosic biomass [44]. The ^1^H and ^13^C NMR spectra and the corresponding peaks and area assignments are shown in Figure 7 and Figure S3a–f. As we can see in the NMR spectra, the humins cover a wide range of frequency domain from 9.8 to 0.9 ppm for proton and from 210 to 10 ppm for carbon. This result confirms the fact that humins are a mixture containing different molecular structures characterized by the presence of carbonyl like ketones (δ_C_ = 210 ppm), aldehydes (δ_C_ = 178.1 ppm, δ_H_ = 9.5 ppm), acids or esters δ_C_ = 173.9 ppm). We can also notice the presence of alkenes δ_C_ from 162.3 to 109.78 ppm and δ_H_ from 7.5 to 6.5 ppm). These functions can be part of a ring like furan or of an alkene segment linking the furanic rings. The presence of a high field sp^3^ CH (between 104 and 70 ppm) and CH_2_ (between 72 and 50 ppm) suggests the existence of strong electron withdrawing groups like oxygen in α and/or α’ positions (O–C–O or C–OH, C–O–C). This fact is not surprising if we consider the carbohydrate origin of humins.

#### 3.4.4. Copolymerization Process

To study the terpolymerization reaction of humins with Capcure and ELO, the three products were firstly individually solubilized in DMSO-d_6_ at room temperature and the resulting solution was heated at 80 °C for 90 h (Figure S4a–b, Figure S5a–b and Figure S6a–b). Thereafter, samples collection of the reaction mixtures prepared in the same conditions (80 °C for 90 and supplementary 446 h) were analyzed by 1D and 2D NMR spectroscopies (Figure S10a–f, Figure S11a–f and Figure S12a–f). Until 398 h, of reaction all the samples were liquid, and no precipitate appears upon cooling from 80 °C to room temperature. After 398 h, the samples become viscous at 25 °C and no more liquid high-resolution NMR experiment could be done.

**Figure 8:**
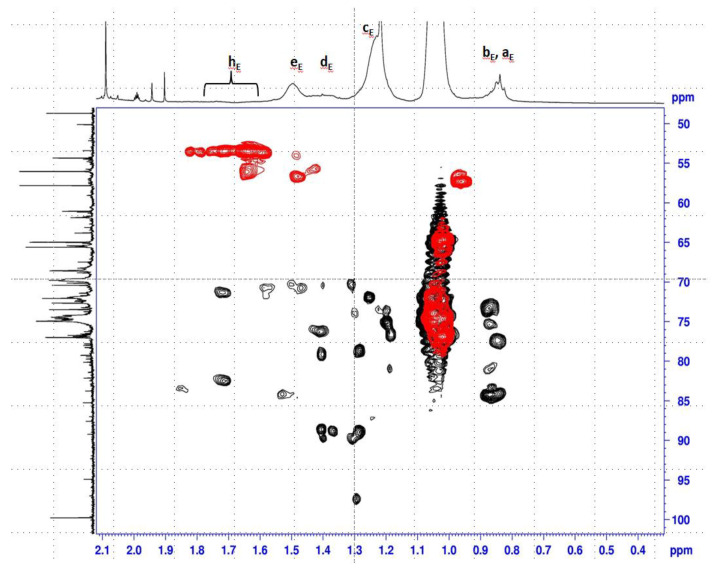
2D HMBC NMR experiments: red HU/ELO/CAP at t = 0, black HU/ELO/CAP after 398 H at 80 °C.

Considering the ELO structure, major changes arise in NMR spectra as the copolymerization process occurs. As we can see in ^1^H (Figure S11a) and in ^13^C (Figure S11b) spectra, the resonance frequencies of signals corresponding to methyl b_E_, methylene d_E_, methylene h_E_, and methyne g_E_ shifts, decrease in intensity, and/or become broader. We can also note that, the resonance peaks belonging to methylene e_E_, f_E_, j_E_, methyne k_E_, and carbonyl i_E_ remain and/or become broader. These results highlight the opening of epoxides groups during the copolymerization process and the fact that the glycerol part of ELO is conserved. 2D HMBC experiment shown in Figure 8 indicates that the opening of epoxide ring leads to new correlation peak between proton h_E_, e_E,_ d_E_ c_E,_ b_E_, and carbon resonance peaks (CH) ranging from 73.2 to 97.52 ppm.

In order to go deeper inside the copolymerization process, we mixed 2 by 2 the start comonomers. Three reaction mixtures (ELO + HU, CAP + HU, ELO + CAP) were solubilized in DMSO d6 in the same proportion as in initial terpolymerization mixture (ELO + CAP + HU) at room temperature and the resulting solutions were heated at 80 °C during 398 h. Sample collections were analyzed through 1D and 2D NMR experiments at 298K (Figure S7a–f, Figure S8a–f and Figure S9a–f)All the samples were liquid and no precipitate appears upon cooling to room temperature, indicating in a first observation that no copolymerization occurred in the analyzed bi-component mixtures.

#### 3.4.5. ELO Reactivity with the HU

**Figure 9:**
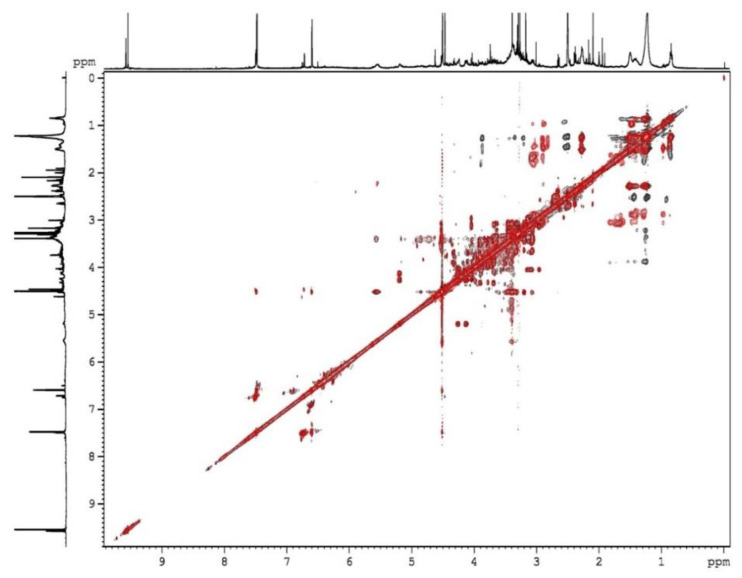
2D TOCSY NMR experiments: red: HU + ELO at t = 0, black HU + ELO after 90 h at 80 °C.

**Figure 10:**
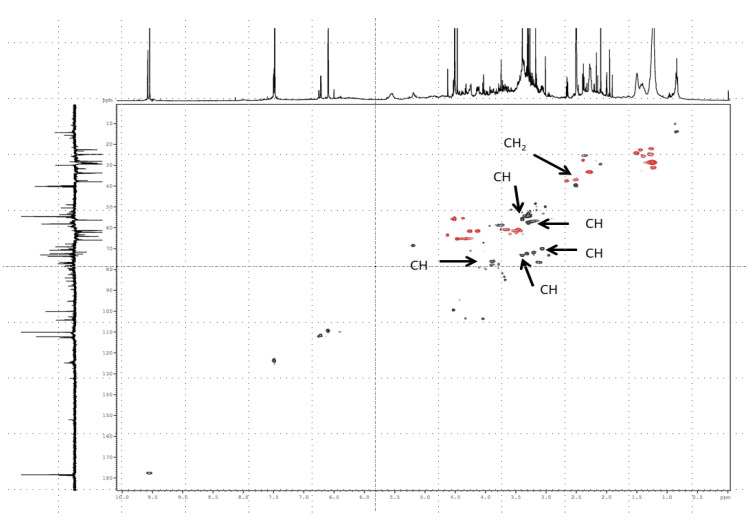
2D HSQC NMR experiments: HU + ELO after 90 h at 80 °C; Arrows indicate the chemical shifts of the carbon that we identified by the new correlation in TOCSY.

**Figure 11:**
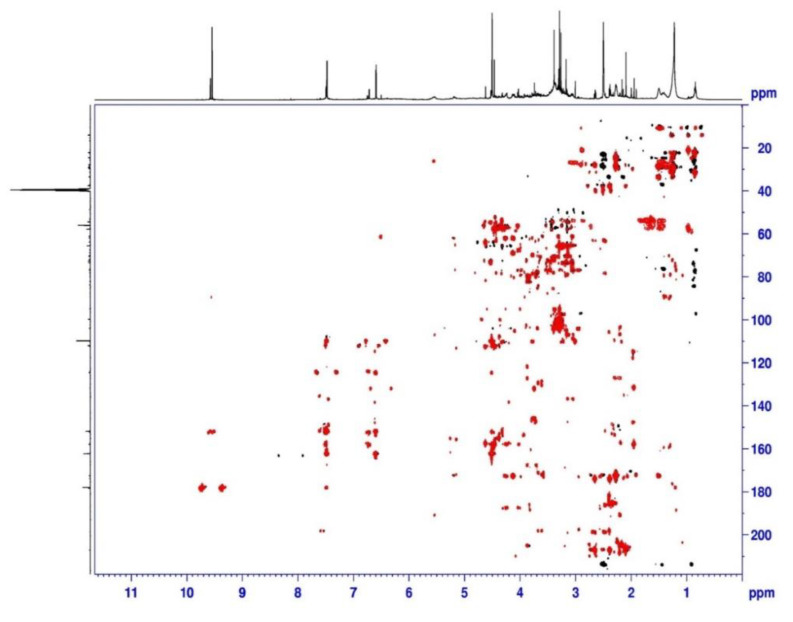
2D HMBC NMR experiments: red HU + ELO at t = 0, black HU + ELO after 90 h at 80 °C.

Figure S9a,b represent the ^1^H and ^13^C NMR data obtained for the mixture of HU and ELO at t = 0 and after 90 h at 80 °C in DMSO. We can firstly observe that the glycerol part of ELO is conserved. We can also detect that changes occurs in the ELO’s structure, particularly the decrease in intensity and shift to height or lower field of the resonance peaks of the epoxide ring (CHg_E_) and those in α or β positions (CH_2_d_E_, CH_2_h_E_, CH_2_c_E_, and CH_3_b_E_). These changes are also noted in 2D NMR experiments: in TOCSY (Figure 9) we can observe new correlation spots between proton in αor β position of epoxide ring (CH_2_d_E_, CH_2_h_E_, CH_2_c_E_, and CH_3_b_E_) and more deshielded protons identified in HSQC (Figure 10) as methylene proton (δ_H_ = 2.49 ppm, δ_C_ = 37 ppm) and methyne proton (δ_H_ = 3.20 ppm, δ_C_ = 57 ppm or/and 71 ppm), (δ_H_ = 3.34 ppm, δ_C_ = 54 ppm or/and 72 ppm), (δ_H_ = 3.87 ppm, δ_C_ = 76–77 ppm)). In HMBC (Figure 11), new correlations appear between CH_3_b_E_ and CH resonance peaks at 68.1–74.4–77.79–84.82 ppm, CH_2_d_E_ and CH resonance peaks at 76.28 ppm. Also, HMBC let us identify the formation of carbonyl function through the correlation between CH_3_b_E,_ CH_2_d_E_, CH_2_ (δ_H_ = 2.48 ppm, δ_C_ = 37.1 ppm—non identified), and ^13^C resonance at 213.77 ppm.

These results are indicating the ring opening of epoxy groups occurs when ELO is mixed and heated at 80 °C with the humins sample. In the literature, many nucleophilic groups [45] were used to open the epoxide ring. Through 1D and 2D NMR spectra the resulting products are characterized by the disappearance of the resonance peak of methyne belonging to epoxide ring and the appearance of new resonance peaks, more deshielded (δ_H_ = 3.1 ppm – 4.6 ppm, δ_C_ = 70.5 ppm – 84.4 ppm), corresponding to secondary alcohol and methyne ether residue [42,46]. Zandvoort et al. [5] indicate that humins by-product are polydisperse sample composed of soluble monomers and/or low molecular weight macromonomers together with some insoluble polymers structure. The opening of the epoxide seems to be due to functional groups present in HU: i.e., –OH, –C=O, CH=O, –COOH. This hypothesis is confirmed by the new signals observed in ^1^H and ^13^C NMR experiments. The observed carbonyl belongs to an ester-type moiety and could result from the reaction of ELO with –COOH moieties found in the humins.

#### 3.4.6. Capcure Reactivity with the HU

Figure S8a,b display the ^1^H and ^13^C NMR data obtained for the mixture of humins and Capcure at t = 0 and after 90 h at 80 °C in DMSO. No changes are observed indicating that in the conditions of our experiment the Capcure did not react with the humins sample.

#### 3.4.7. ELO Reactivity with the Capcure

**Figure 12:**
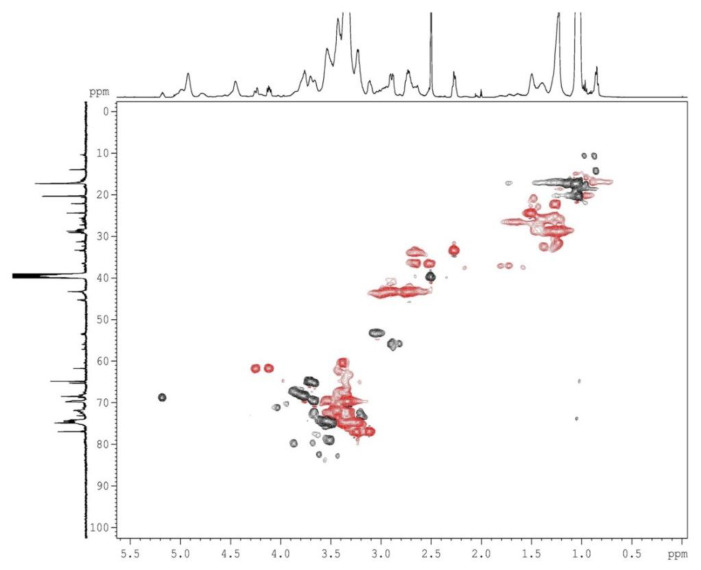
2D HSQC NMR experiments red: CAP+ ELO at t = 0, black CAP + ELO at t = 90 h at 80 °C.

**Figure 13:**
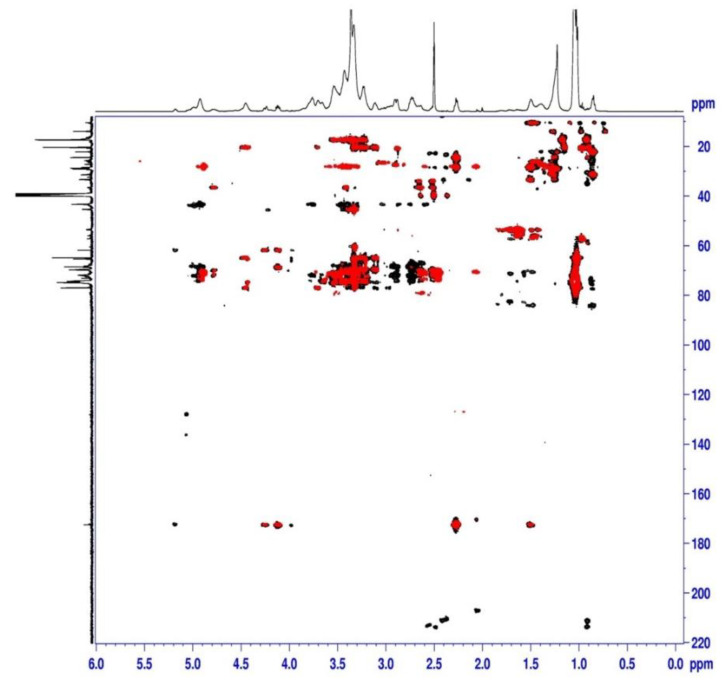
2D HMBC NMR experiments red CAP+ ELO at t = 0, black CAP + ELO at t = 90 h at 80 °C.

Figure S7a,b illustrate the ^1^H and ^13^C NMR spectra for the mixture of ELO and Capcure at t = 0 and after 90 h at 80 °C in DMSO. As shown in Figure S5a–b, we can notice that the glycerol part of ELO is conserved when heated at 80 °C. We can also highlight that changes occurs for ELO in particularly the resonance peaks of the epoxide ring (CHg_E_) and those in α or β positions (CH_2_d_E_, CH_2_h_E_, CH_2_c_E_, and CH_3_b_E_) that decrease in intensity and are shifted to higher or lower field. The HSQC and HMBC 2D NMR experiments (Figure 12 and Figure 13) permit us to identify new correlations between CH_3_b_E_ and CH resonance peaks at 73, 74.5, 75.6, and 83.95 ppm; the CH2 resonance at 75.52–77.39 ppm, CH_2_d_E_ and CH2 resonance peaks at 70.58 ppm; CH resonance peaks at 84.11 ppm, CH_2_h_E_ and CH resonance peaks at 70.99–82.25 ppm. These results are indicating the opening of the epoxide rings in presence of CAP at 80 °C. Potentially the opening is possible through OH and/or SH reactive groups of CAP. In our case, we can observe the disappearance of the resonance of thiol proton. On the other hand, we cannot see, qualitatively, changes in intensity of alcohol proton belonging to CAP. Together with the consideration that the nucleophilicity of sulphur is higher that the oxygen one [46,47,48], these facts are in favor of the preferential opening of epoxide ring by thiol group.

## 4. Conclusions

In this work, the pathways of terpolymerization between humins, Capcure, and ELO were investigated. Our study showed that, in solution, ELO manifests reactivity towards humins. Moreover, in a binary mixture, Capcure does not manifest reactivity towards the humins. NMR studies have confirmed the reaction mechanisms proposed both in the case of humins/ELO mixtures as well as in the case of Capcure/ELO. The entirety of the terpolymerization could not be completely assessed. The complexity of the spectra did not allow for the assessment of the reactivity presented by the intermediaries of the Capcure–ELO reaction towards humins. The same experiment was performed via rheology and IR spectroscopy. The employment of these methods in real time through the course of the polymerization provided supplementary information both about the chemical modifications through the formation of the material, as well as about the physical parameters of the polymerization.

Since the use of technical grade starting materials (humins) raises new challenges in terms of the characterization of the chemical copolymerization reaction, this study provides suggestions on the strategy and approach for this type of materials synthesis, as well as for appropriate means of analysis. As the push for eco-friendly materials becomes more urgent than ever, flexible reactions—applied to by-products as starting materials—will become more enticing and pervasive.

## Figures and Tables

**Table 1:** ^1^H and ^13^C NMR (DMSO d6) Assignments of ELO.

Signal	^13^C δ ppm	^1^H δ ppm	Structure
a_E_	13.75–13.93	0.81–0.88	CH_3_–(CH_2_)_3_-
b_E_	10.23–10.55	0.93–0.99	CH_3_–CH_2_ -epoxide
c_E_	21.94–22.17 28.14–29.15 31–31.4	1.18–1.33	(CH_2_) β group (epoxide, carbonyl)
d_E_	20.51–20.74 25.51–26.15 27–27.43	1.34–1.47	(CH_2_) α group (epoxide, carbonyl)
e_E_	24.23–24.51	1.47–1.55	(CH_2_) β group (carbonyl)
f_E_	33.20–33.50	2.21–2.31	–CH_2_–C=O
g_E_	57.22–53.15	2.84–3.12	CH epoxide
h_E_	26.26–26.63	1.56–1.88	(CH_2_) α, α’ epoxide
i_E_	172.01–172.57	/	C=O
j_E_	61.75	4.29–4.77	–CH_2_–O
k_E_	68.73	5.14–5.21	O–CH–(CH_2_–O)_2_

**Table 2:** ^1^H and ^13^C NMR (DMSO d6) assignments of Capcure; R1 = CH_2_–O–CH_2_–CH(CH_3_) –O–CH_2_–CH(OH)–CH_2_–SH.

Signal	δ ppm ^1^H	δ ppm ^13^C	Structure
1_c_	2.08 (t)	/	SH
2_c_	2.47 (m); 2.61 (m)	28.07	CH_2_–SH
3_c_	3.85 (m)	70.9	CH–OH
4_c_	4.91	/	OH
5_c_	3.58 (m)	77.06–74.73 * 73.7–69.92 * 69.88–69.38 *	HO–CH–CH_2_–O
6_c_	3.68 (m); 3.52(m)	64.96; 74.47	CH–CH_3_
7_c_	1.02 (m); 1.04 (m)	20.35; 17.24	CH_3_–CH
8_c_	3.23; 3.33	77.06–73.73 * 73.7–69.92 * 69.88–69.38 *	CH_3_–CH–CH_2_–O
9_c_	3.342	77.06–74.73 * 73.7–69.92 * 69.88–69.38*	(R1)_3_–C–CH_2_–O
10_c_	/	45.27	(R1)_3_–C–R1

## Supplementary Materials

The following are available online at https://www.mdpi.com/2073-4360/12/7/1583/s1, Figure S1a–f: 1D and 2D NMR data for ELO; Figure S2a–f: 1D and 2D NMR data for Capcure; Figure S3a–f: 1D and 2D NMR data for Humins; Figure S4a–b: 1D NMR data for Humins after 90 H at 353 K; Figure S5a–b: 1D NMR data for ELO after 90 H at 353 K; Figure S6a–f: 1D NMR data for Capcure after 90 H at 353 K; Figure S7a–f: 1D NMR data for Capcure + ELO after 90 H at 353 K; Figure S7a–f: 1D NMR data for Capcure + ELO after 90 H at 353 K; Figure S8a–f: 1D NMR data for Capcure + Humins after 90 H at 353 K; Figure S9a–f: 1D NMR data for ELO + Humins after 90 H at 353 K; Figure S10a–f: 1D NMR data for ELO + Humins + Capcure; Figure S11a–f: 1D NMR data for ELO +Humins+ Capcure after 90 H at 353 K; Figure S12a–f: 1D NMR data for ELO + Humins + Capcure after 238 H at 353 K; Table S1: IR attributions of the signals found in the spectra.
