# Supplementary material for: Structural Insights of Humins/Epoxidized Linseed Oil/ Hardener Terpolymerization
Source: Polymers (Basel). 2020 Jul 16;12(7):1583. doi: 10.3390/polym12071583 (PMC7408238; doi:10.3390/polym12071583)
Supplement: Supplementary file 1 [file polymers-12-01583-s001.pdf]

Supporting information

Structural insights of terpolymerization of a biorefinery by-product with a vegetable oil derived epoxide

Erol Licsandru, Marc Gaysinski, Alice Mija*

Université Côte d'Azur, Institut de Chimie de Nice, UMR CNRS 7272, 06108 Nice Cedex 02, France
*Correspondence author: Alice.MIJA@univ-cotedazur.fr

Table of contents

Table S1: IR attributions of the signals found in the spectra.	P. 2
NMR spectroscopy – experimental part	P. 3
**Figures S1a –f.** 1D and 2D NMR data for ELO	P. 4-9
**Figures S2a –f.** 1D and 2D NMR data for Capcure	P. 10-15
**Figures S3a –f.** 1D and 2D NMR data for Humins	P. 16-21
**Figures S4a –b.** 1D NMR data for Humins after 90 H at 353 K	P. 22-23
**Figures S5a –b.** 1D NMR data for ELO after 90 H at 353 K	P. 24-25
**Figures S6a –f.** 1D NMR data for Capcure after 90 H at 353 K	P. 26-31
**Figures S7a –f.** 1D NMR data for Capcure + ELO after 90 H at 353 K	P. 32-37
**Figures S8a –f.** 1D NMR data for Capcure +Humins after 90 H at 353 K	P. 38-43
**Figures S9a –f.** 1D NMR data for ELO +Humins after 90 H at 353 K	P. 44-49
**Figures S10a –f.** 1D NMR data for ELO +Humins+ Capcure	P. 50-55
**Figures S11a –f.** 1D NMR data for ELO +Humins+ Capcure after 90 H at 353 K	P. 56-61
**Figures S12a –f.** 1D NMR data for ELO +Humins+ Capcure after 238 H at 353 K	P. 62-67

Table 1. IR attributions of the signals found in the spectra.

Wavenumbers (cm^{-1})	Assignments	Found in
3400	O-H stretching	Resin, HU
2930; 2845	C-H stretching	Resin, HU, ELO, CAP
1740	C=O stretching aldehyde	resin
1735	C=O stretching ester	resin, ELO
1685	C=O stretching α-β unsaturated	resin, HU
1660	C=O stretching α-β unsaturated	HU
1625	C=C stretching furan ring conjugated with C=O	resin, HU
1510	C=C stretching furan ring	resin, HU
1460	CH_2 deformation	resin, HU
1445	CH_2 deformation	ELO, CAP
1420	O-H bending (in plane)	CAP
1375	CH_2 deformation	Resin, HU, ELO, CAP
1350	CH_2 deformation	HU
1265	C-O stretching (sy) substituted furan	resin
1250	C-O stretching alcohols	CAP, HU
1240	C-O stretching oxirane	ELO
1180	C-O stretching furan ring (sy)	HU
1140	C-O stretching α from ester group	ELO
1100	C-O stretching (C_3H_7-O)	CAP
1084	C-O stretching ester group	ELO
1069	C-O stretching (as) substituted furan split	resin
1045	C-O stretching (as) furan split	HU
1020	C-O stretching (as) substituted furan split	resin
1010	C-O stretching (as) furan split	HU
960	=C-H &=CH_2 bending furan	resin, HU
900	C-H bending	CAP
850	=C-H &=CH_2 bending (out of plane bending)	resin
830	C-H bending O-C-H	ELO, CAP
750	C-H bending furan	resin, HU
715	C-H bending	ELO

NMR spectroscopy - *Experimental part*

^{1}H spectra were acquired using: 10.3 KHz spectral width (SW), 64K complex data point, acquisition time (aq) of 3.17s, relaxation delay (D1) of 1s, number of scan (ns) of 32 and a 30° flip angle pulse width.
^{13}C{^{1}H} spectra were acquired using : 37.5 KHz for SW, 64K complex data point , 0.87s for aq , D1 of 2s, ns of 4000 and 30° flip angle pulse width. ^{1}H decoupling was achieved using WALTZ 16 pulse sequence. Prior to Fourier transformation, the fids were multiplied by an exponential line broadening function of 1Hz.
DEPT135 and DEPT90 spectrum were acquired using: 37.5 KHz for SW, 64K complex data point, 0.87s for aq, D1 of 2s, ns of 2000 and 90° flip angle pulse width. ^{1}H decoupling was achieved using WALTZ 16 pulse sequence. Prior to Fourier transformation, the fids were multiplied by an exponential line broadening function of 1Hz.
gs-COSY spectra were obtained with a spectral width of 6 KHz in both dimensions, 2K complex data point in F2, 256 t1 increments (8 scans by increment) in F1, 0.17s for aq, D1 of 2s. Prior to Fourier transformation the data were zero filled in F1.
gs-HSQC[16] phase sensitive (echo - antiecho mode) was obtained with a spectral width of 6 KHz and 1K complex data point in F2 and a spectral width of 20.8KHz and 256 t1 increment (32 scans by increment) in F1. Others main parameters are : 0.114s for aq, 1.5s for D1. Prior to Fourier transformation a QSINE window function (SSB =2) was applied in both dimension and the data were zero filled and linear predicted (NC=32) to 1K data points in F1
gs-HMBC was acquired with a spectral width of 6KHz and 1K complex data point in F2 and a spectral width of 30.18KHz and 256 t1 increment (64 scans by increment) in F1. Others main parameters are : 0.34s for aq, 1.5s for D1, 8Hz for J(X-H) long range coupling and 145 Hz for ^{1}j (X-H). Prior to Fourier transformation a SINE window function (SSB =0) was applied in both dimension and the data were zero filled and linear predicted (NC=32) to 1K data points in F1
gs-TOCSY[2,3,4] Phase sensitive (States – TPPI mode) experiments using MLEV 17 pulse sequence for spin lock were with spectral width of 6 KHz in both dimensions, 2K complex data point in F2, 256 t1 increments (20 scans by increment) in F1, 0.17s for aq and D1 of 2s. MLEV 17 pulse sequence for spin lock was set to 100ms. Prior to Fourier transformation a QSINE window function (SSB =2) was applied in both dimension and the data were zero filled and linear predicted (NC=32) to 1K data points in F1.

I - ELO

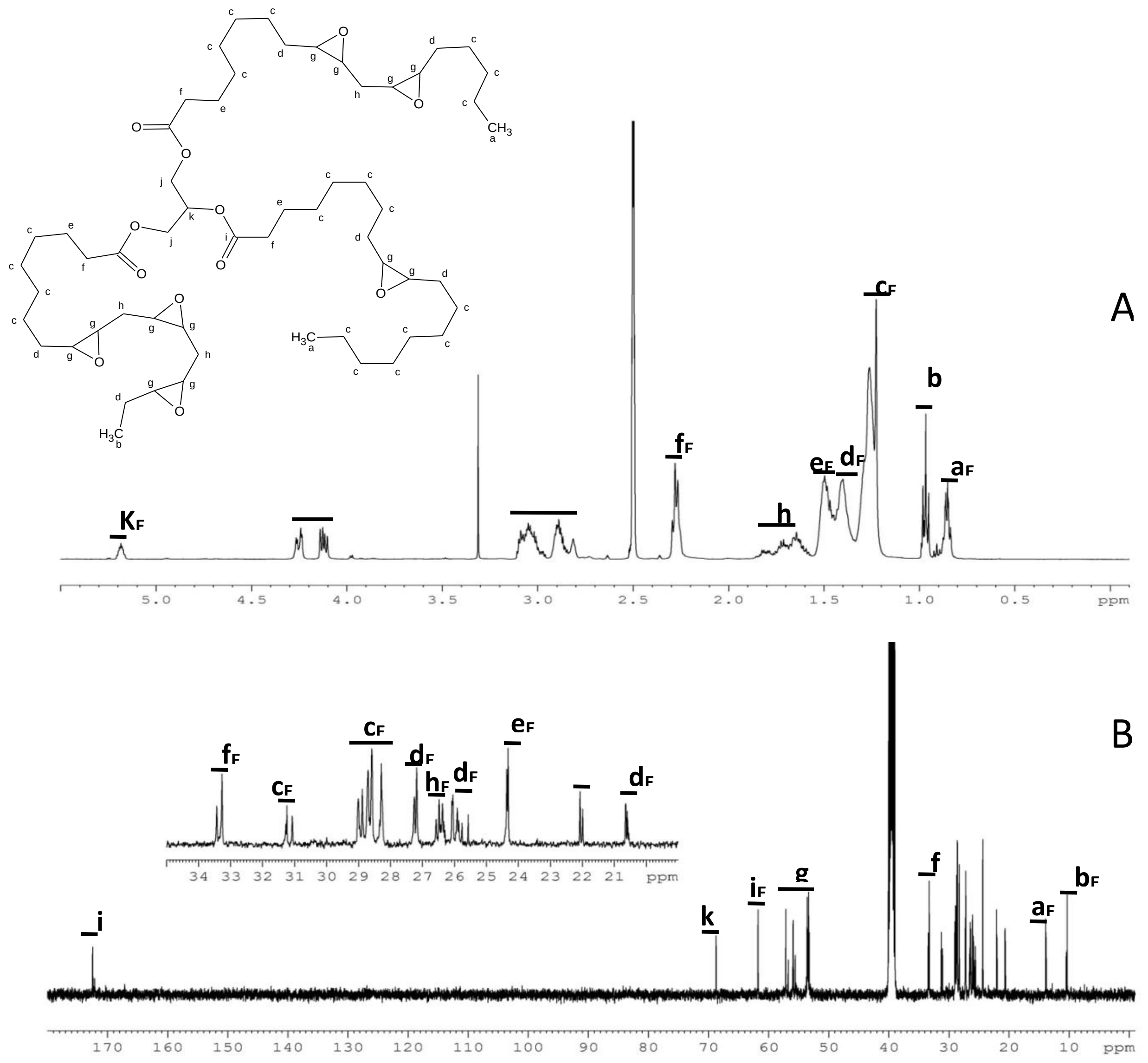

Figure S1a – ^{1}H-NMR and ^{13}C NMR spectra for ELO (DMSO d6)

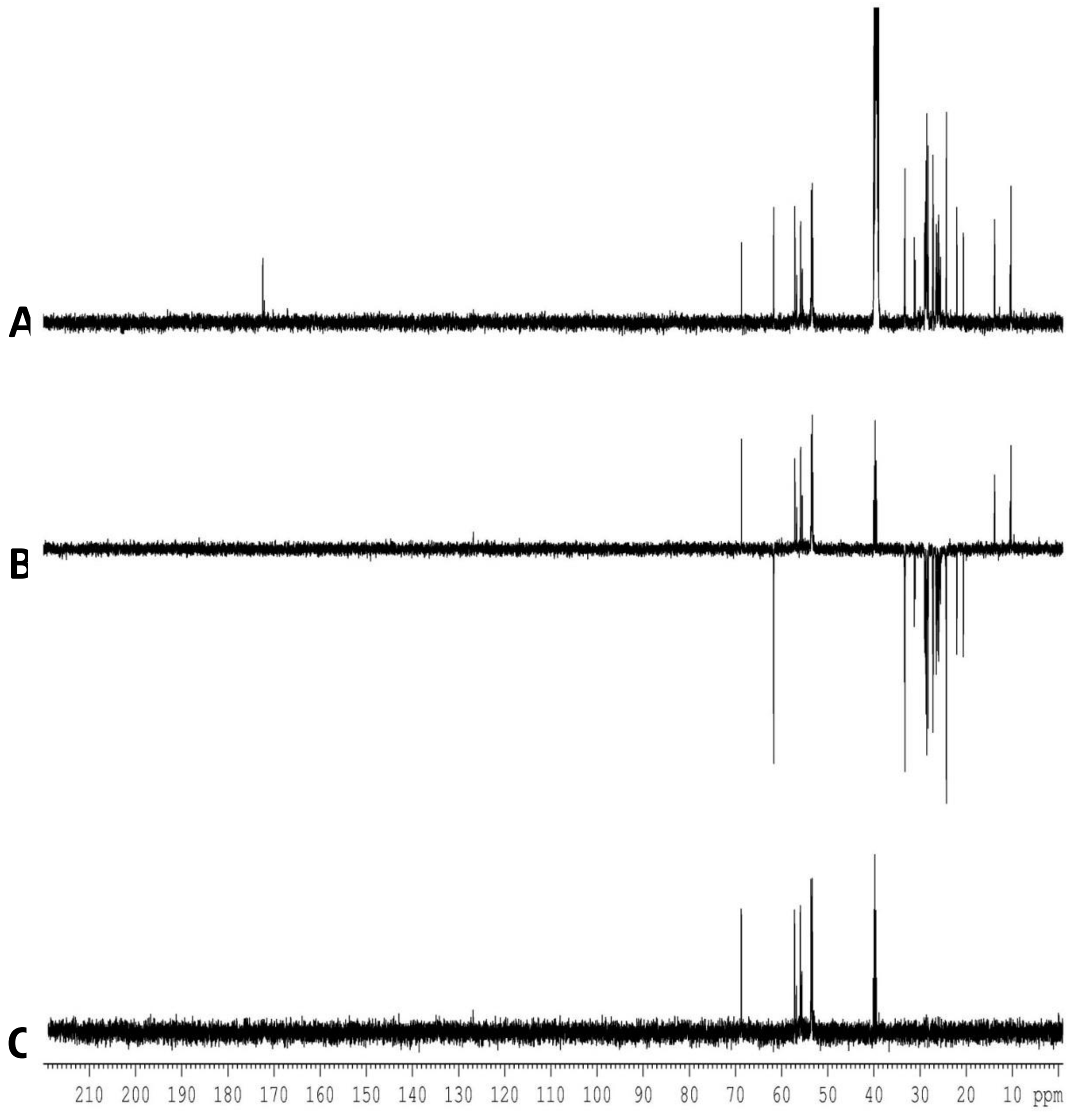

Figure S1b – ^{13}C-NMR spectrum for ELO - A : C13CPD - B : DEPT135 : C : DEPT90

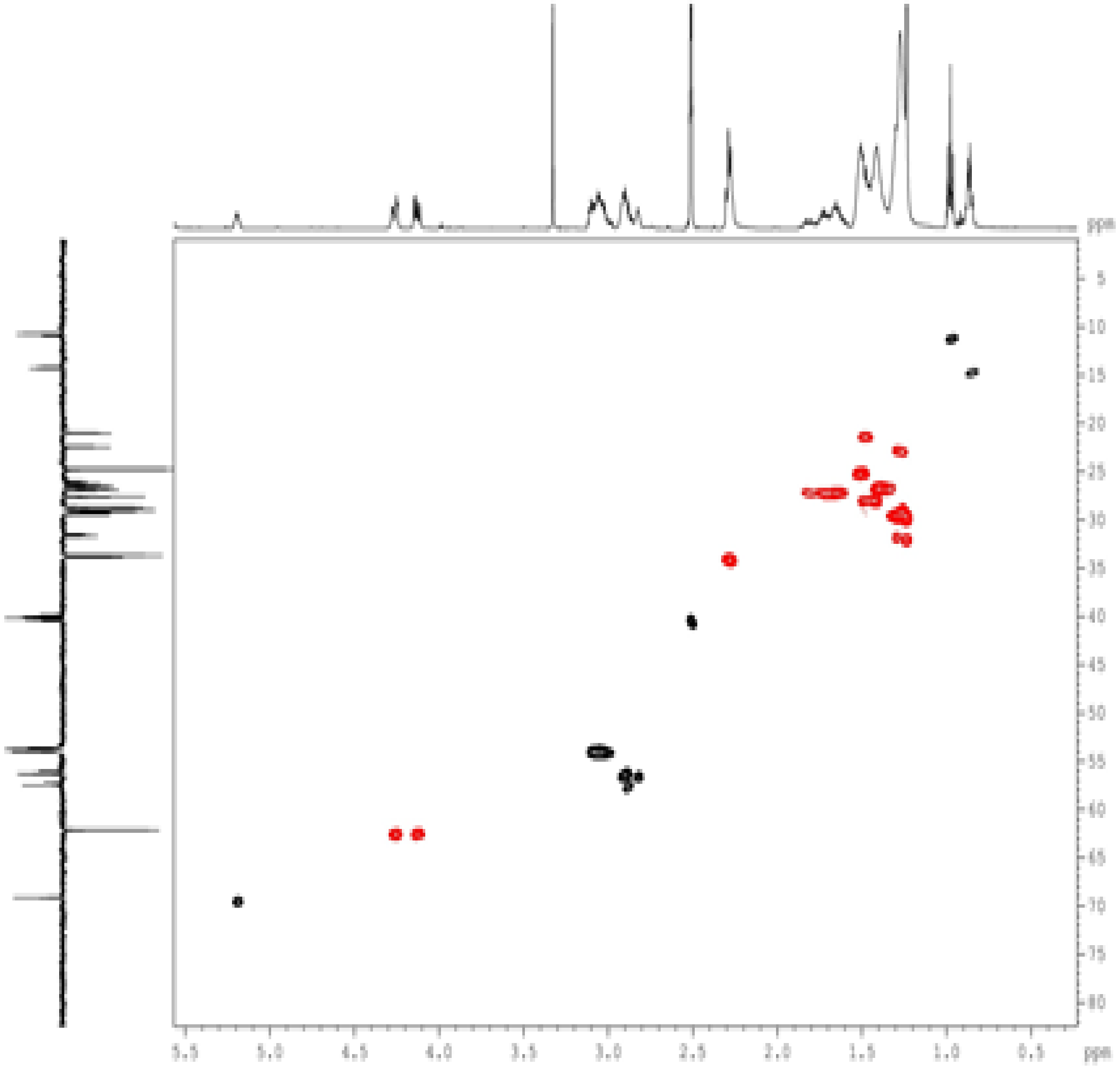

Figure S1c – HSQC spectrum for ELO

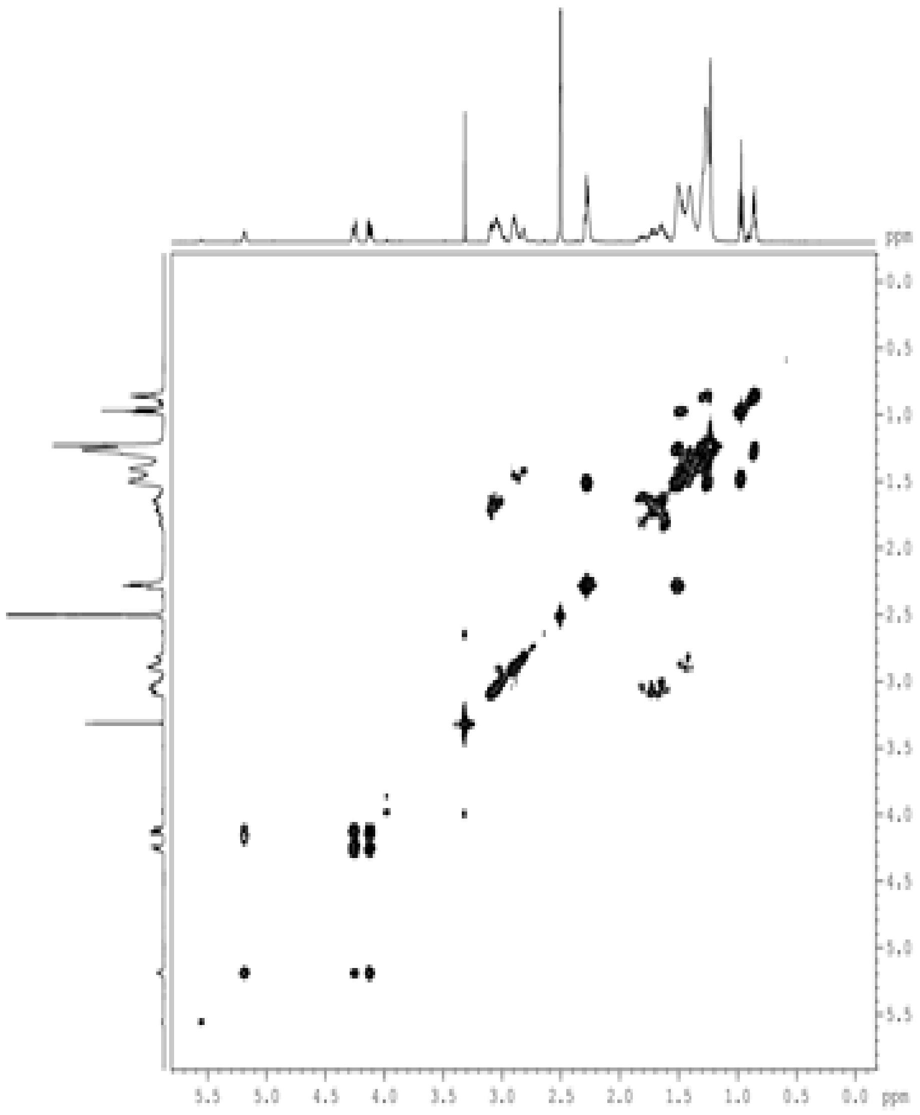

Figure S1d – COSY spectrum for ELO

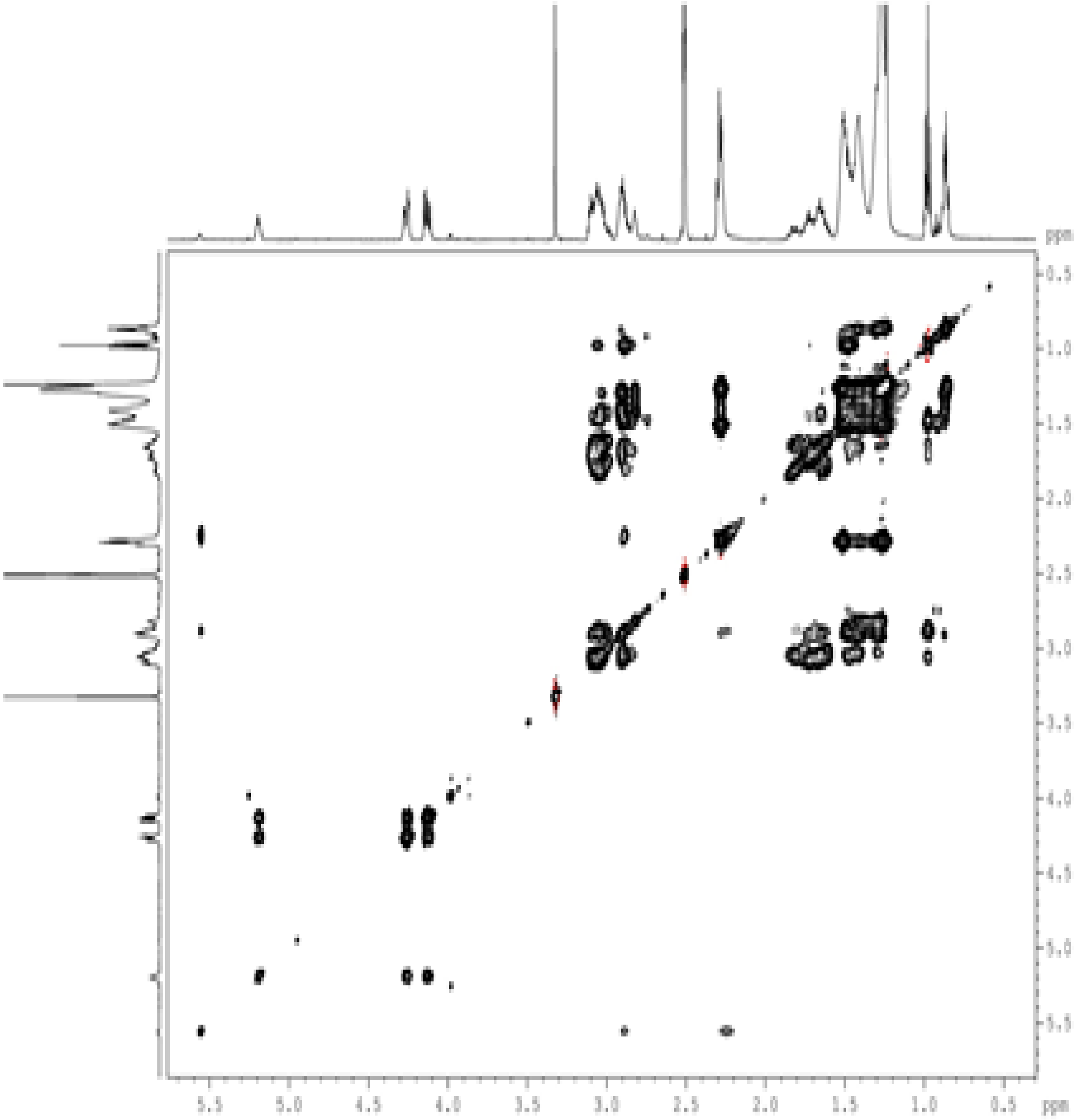

Figure S1e – TOCSY spectrum for ELO

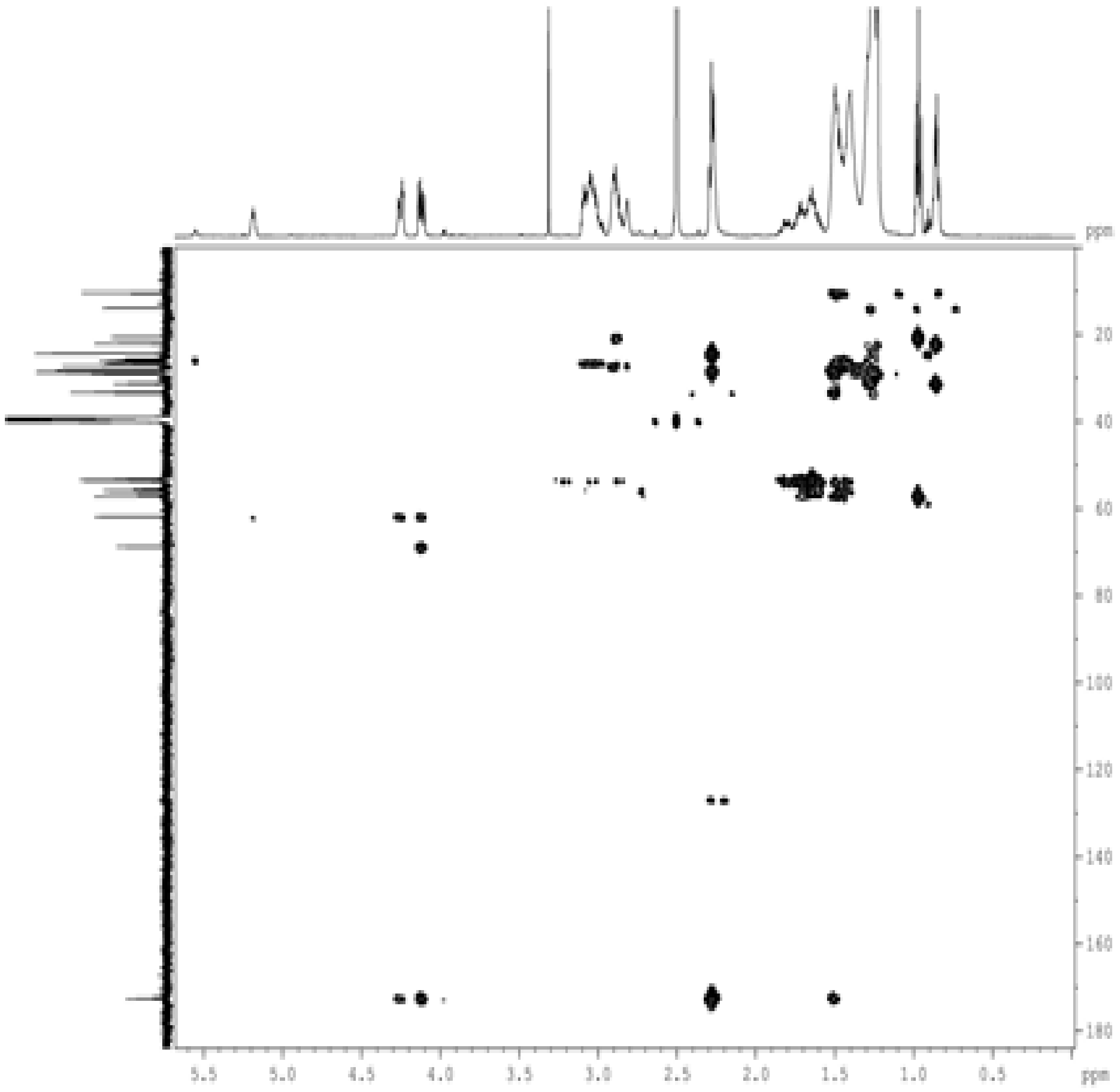

Figure S1f – HMBC spectrum for ELO

II – Capcure

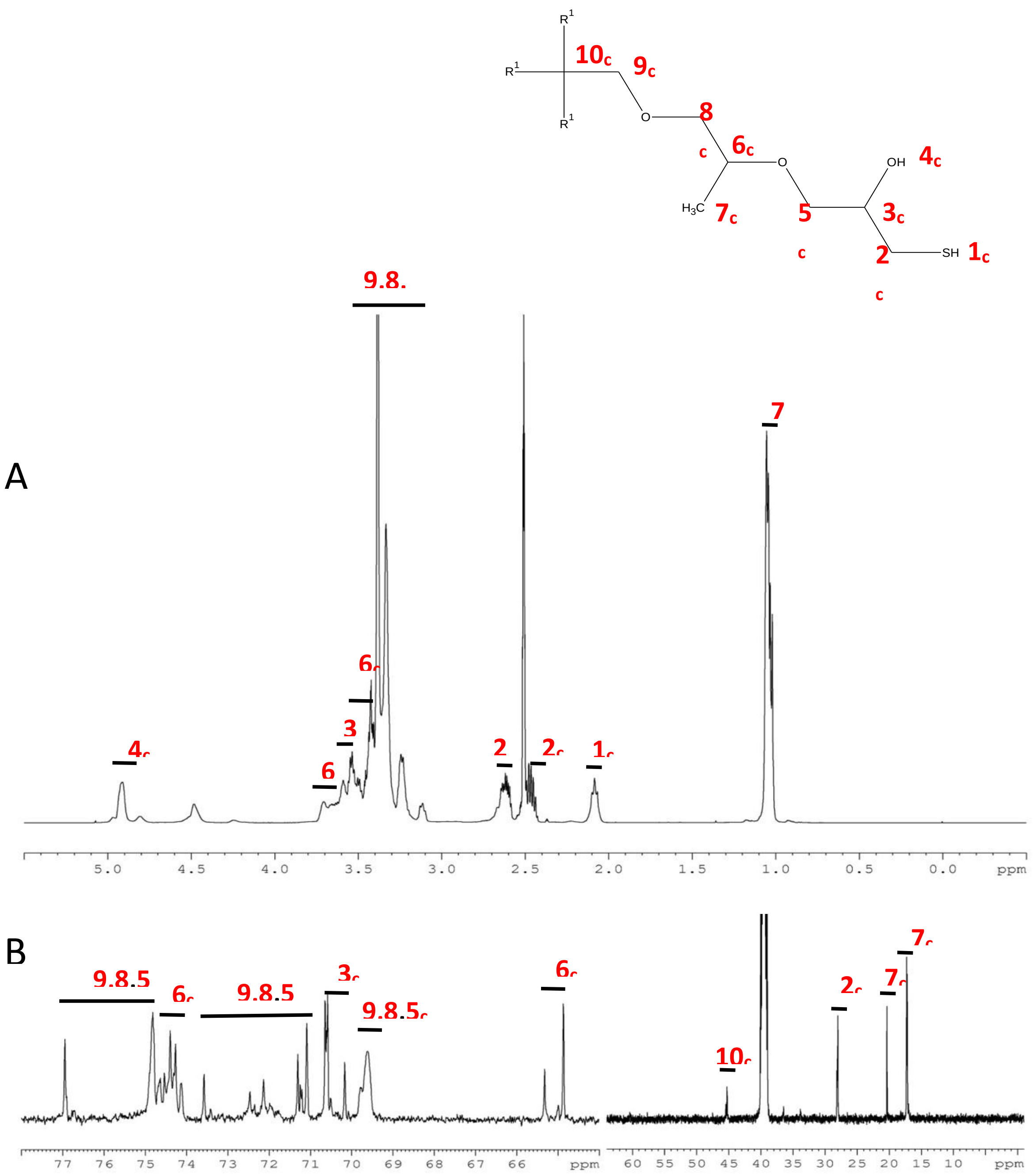

Figure S2a – A) ^{1}H-NMR and B) ^{13}C NMR spectra for Capcure (DMSO d6) - R1 = CH_2-O-CH_2-CH(CH_3)-O-CH_2-CH(OH)-CH_2-SH

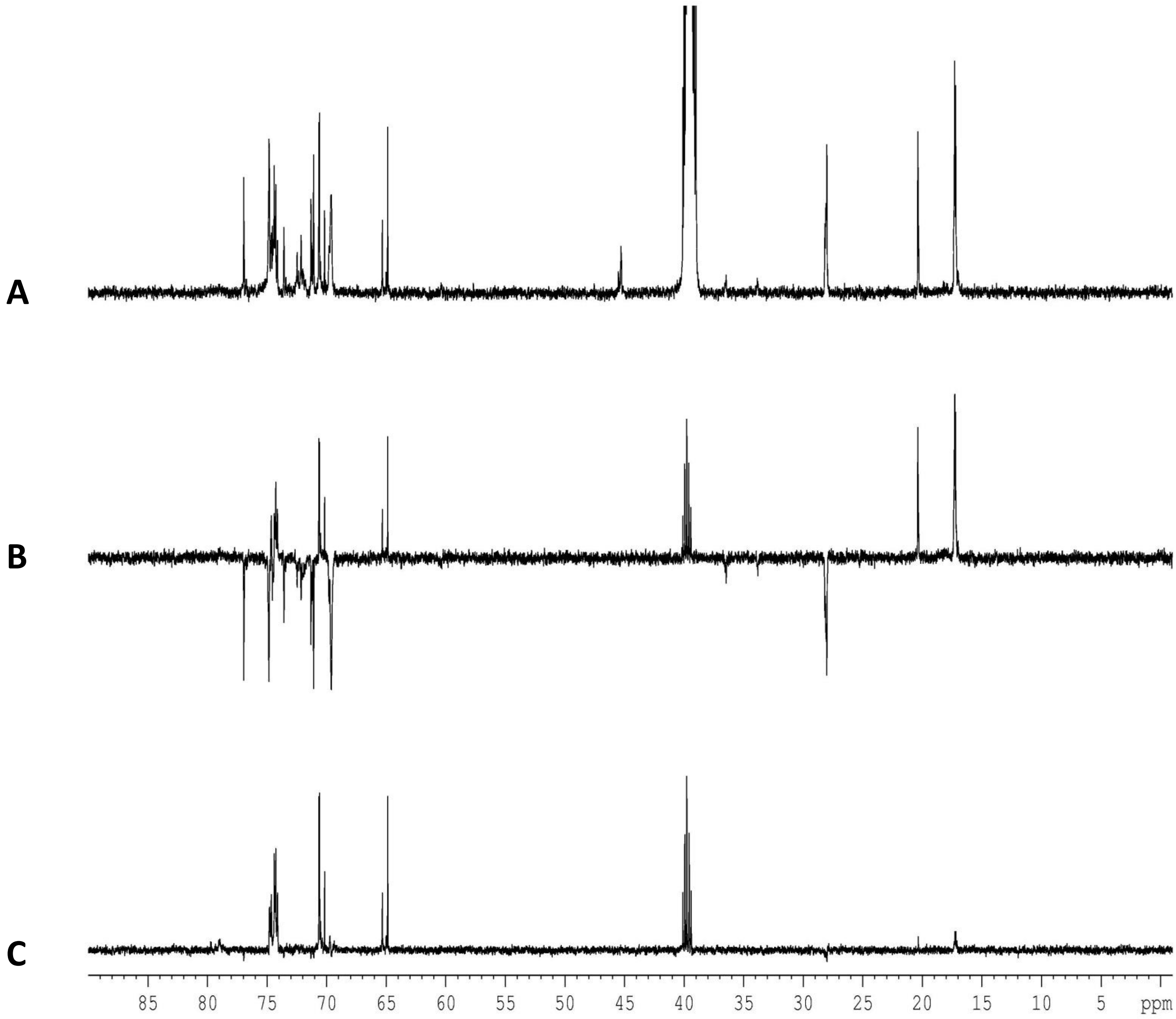

Figure S2b – ^{13}C-NMR spectrum for Capcure - A : C13CPD - B : DEPT135 : C : DEPT90

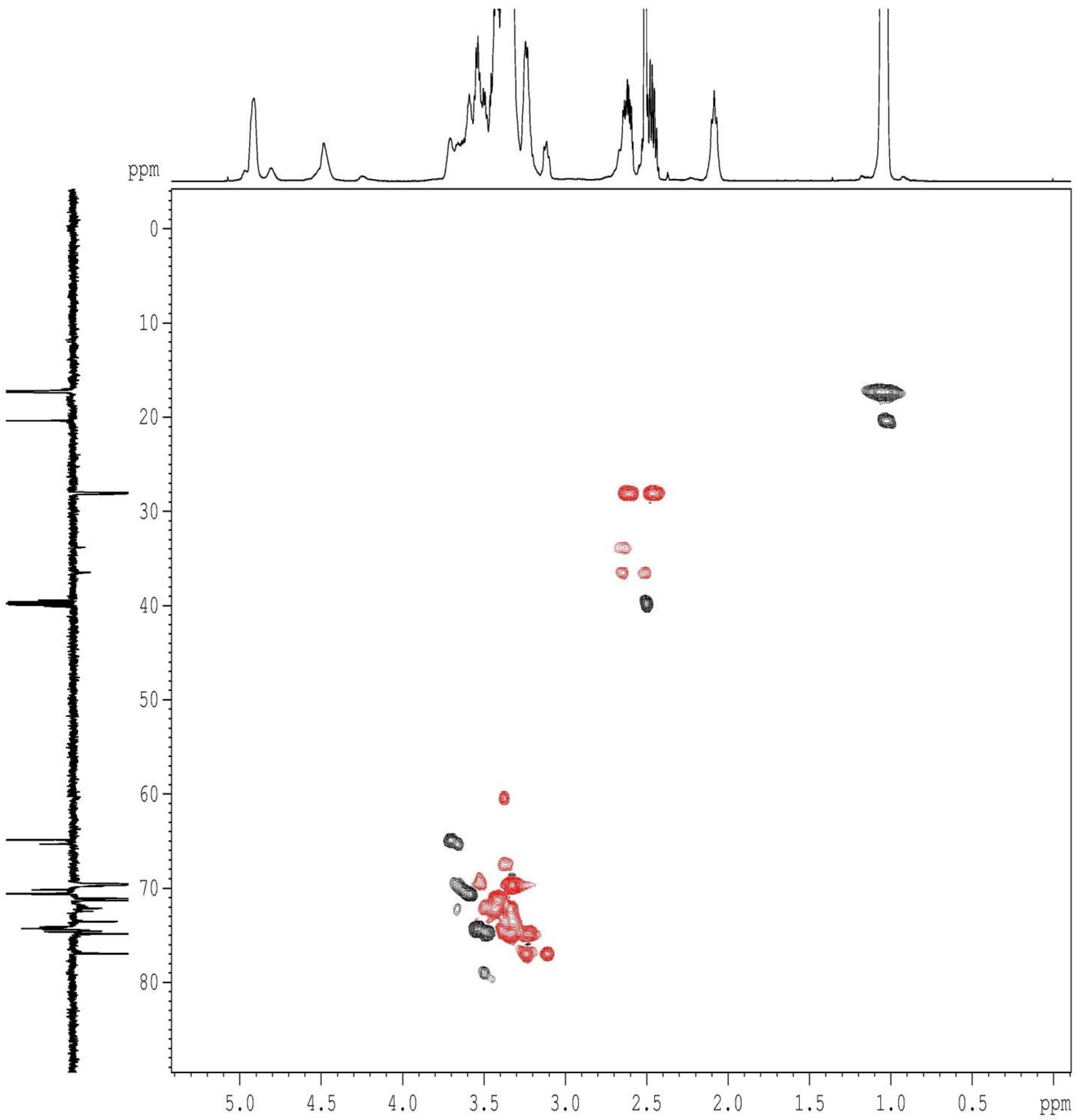

Figure S2c – HSQC spectrum for Capcure

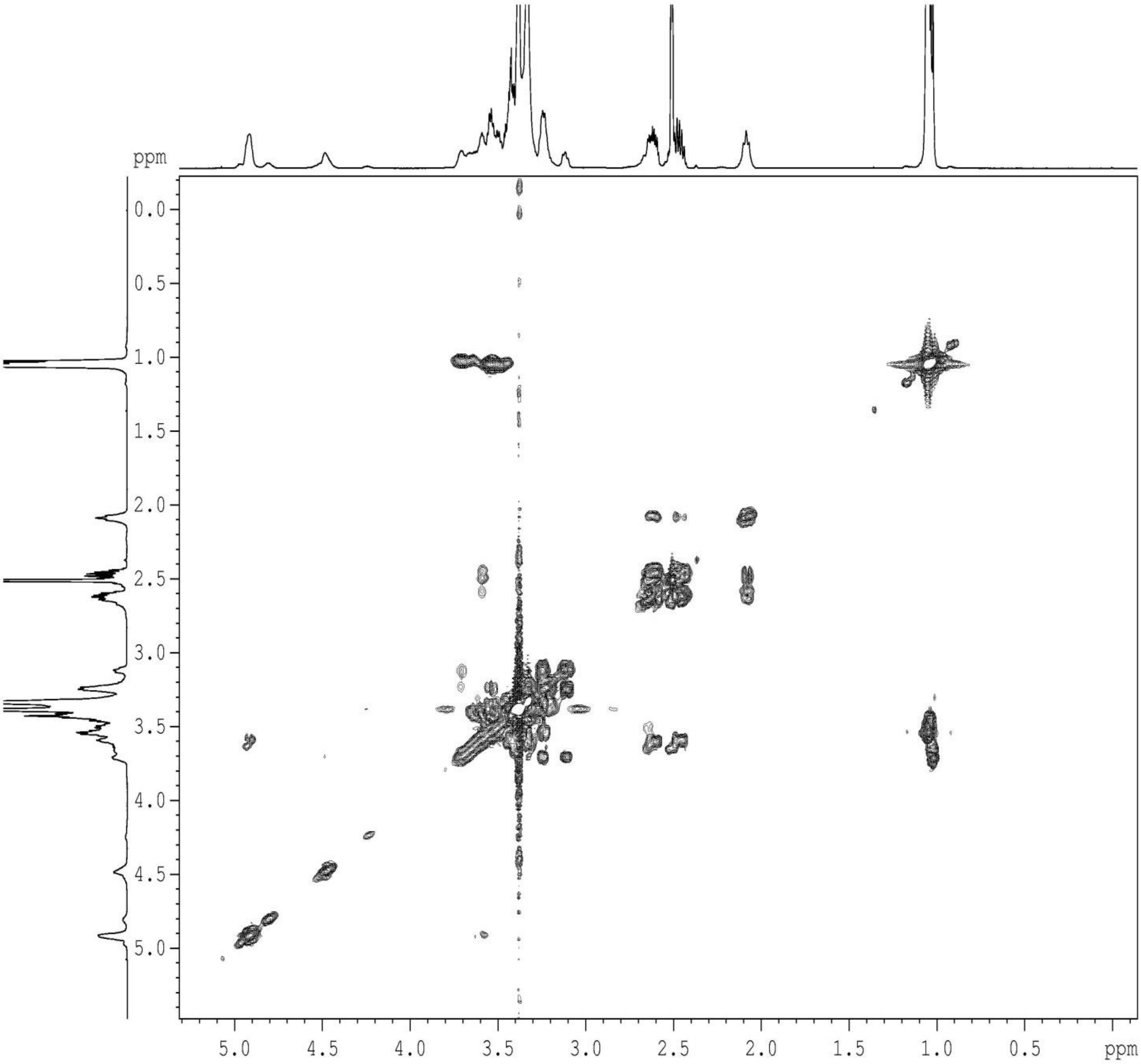

Figure S2d – COSY spectrum for Capcure

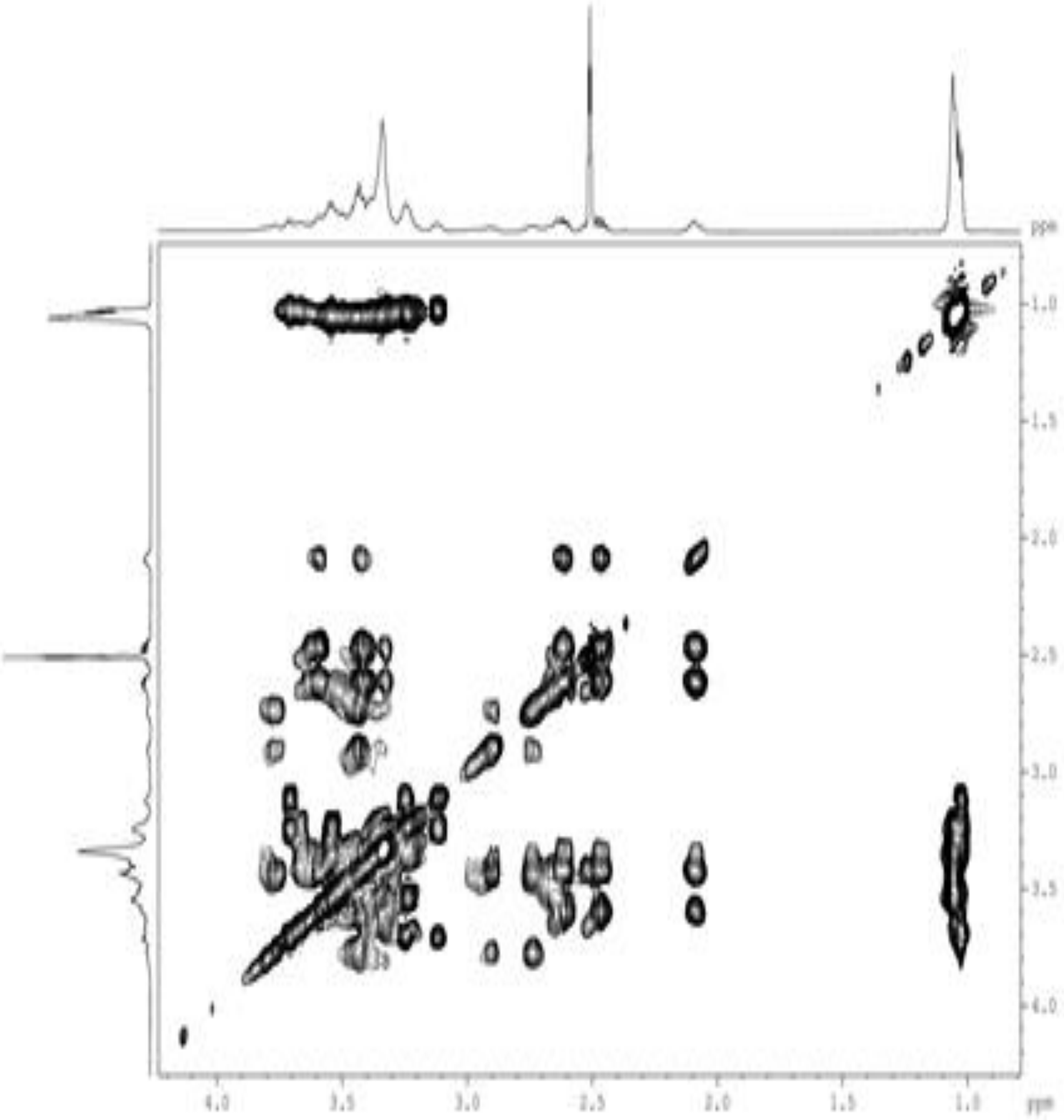

Figure S2e – TOCSY spectrum for Capcure

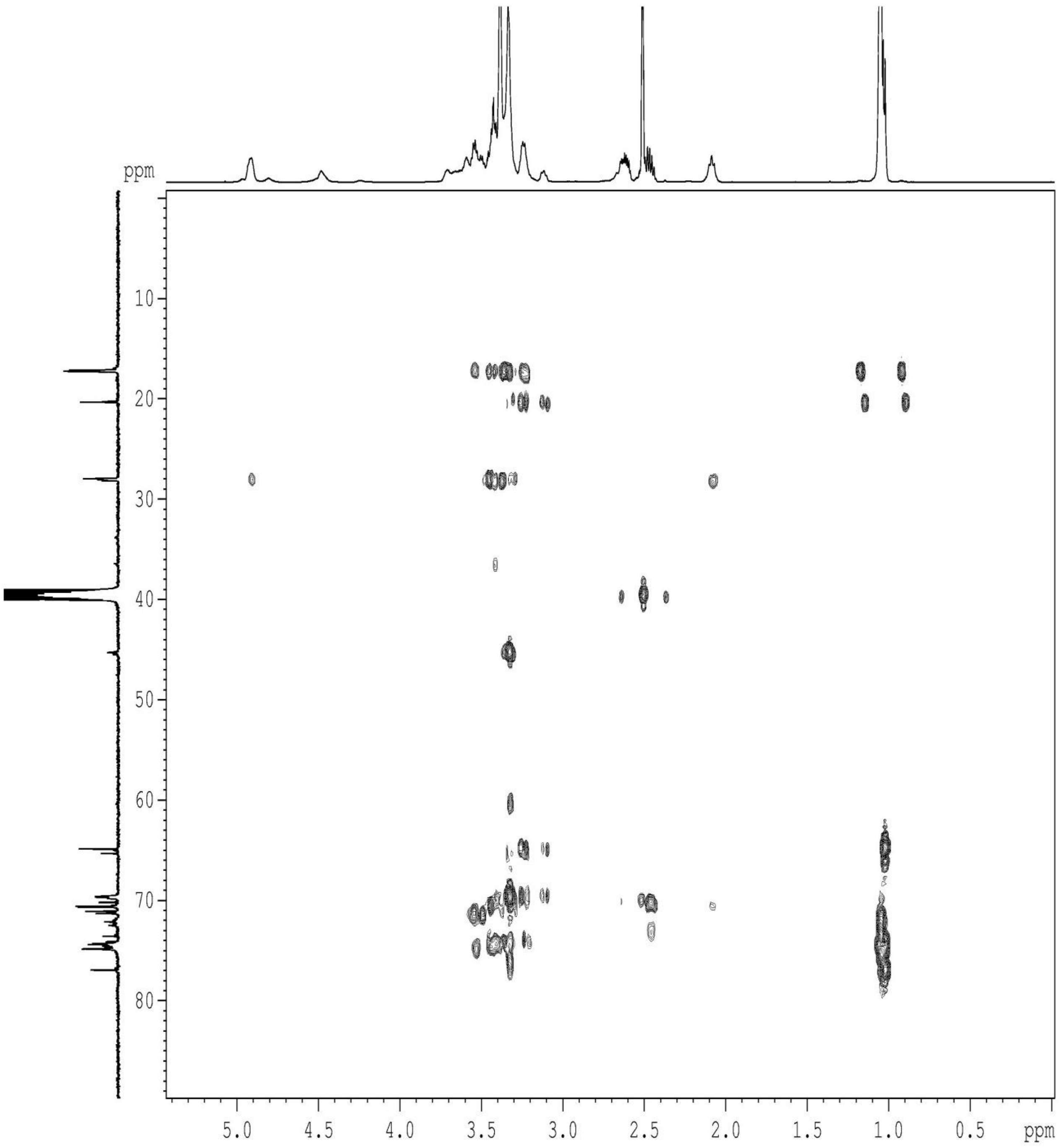

Figure S2d – HMBC spectrum for Capcure

III – Humins

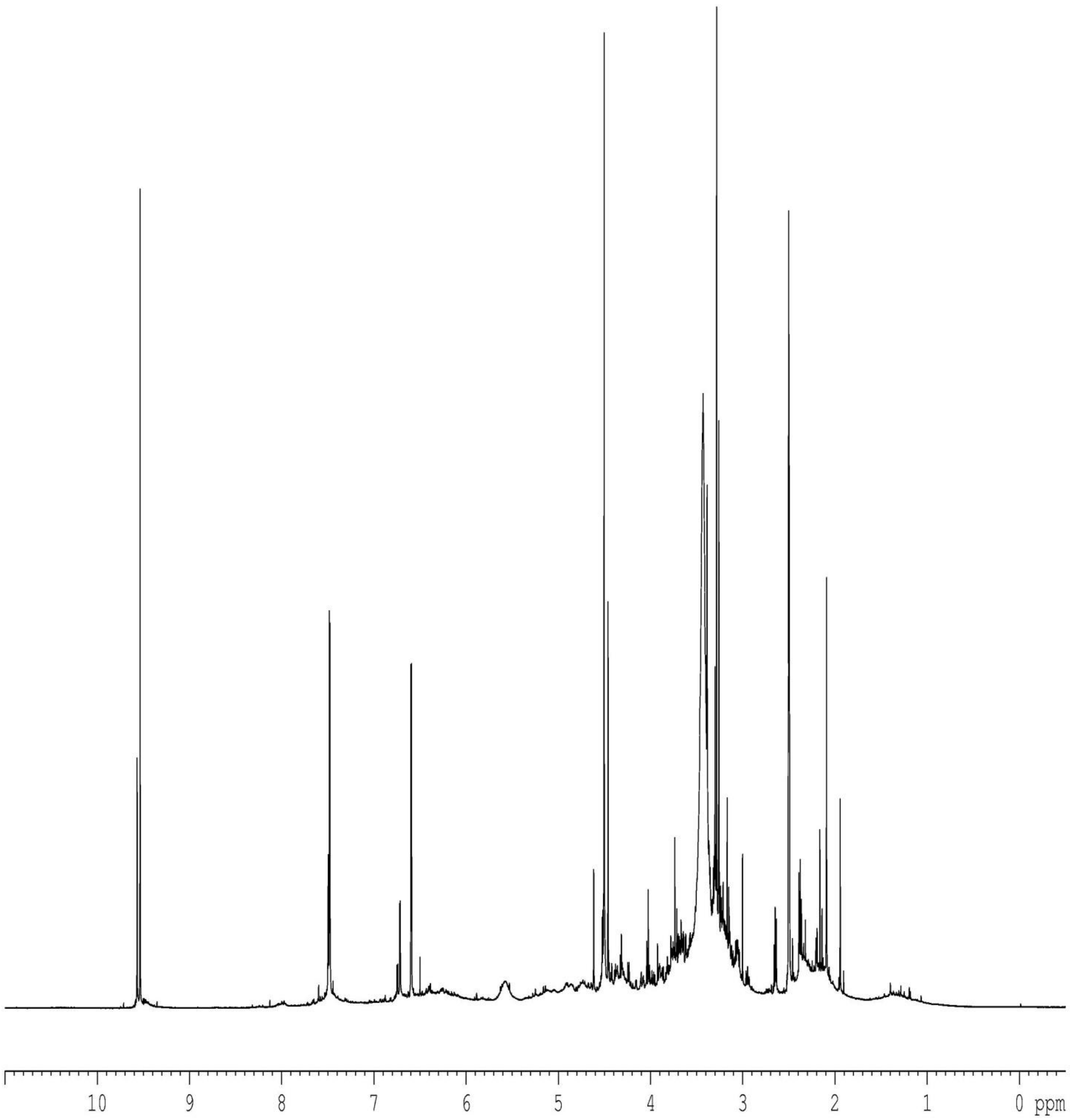

Figure S3a – ^{1}H-NMR spectrum for Humins

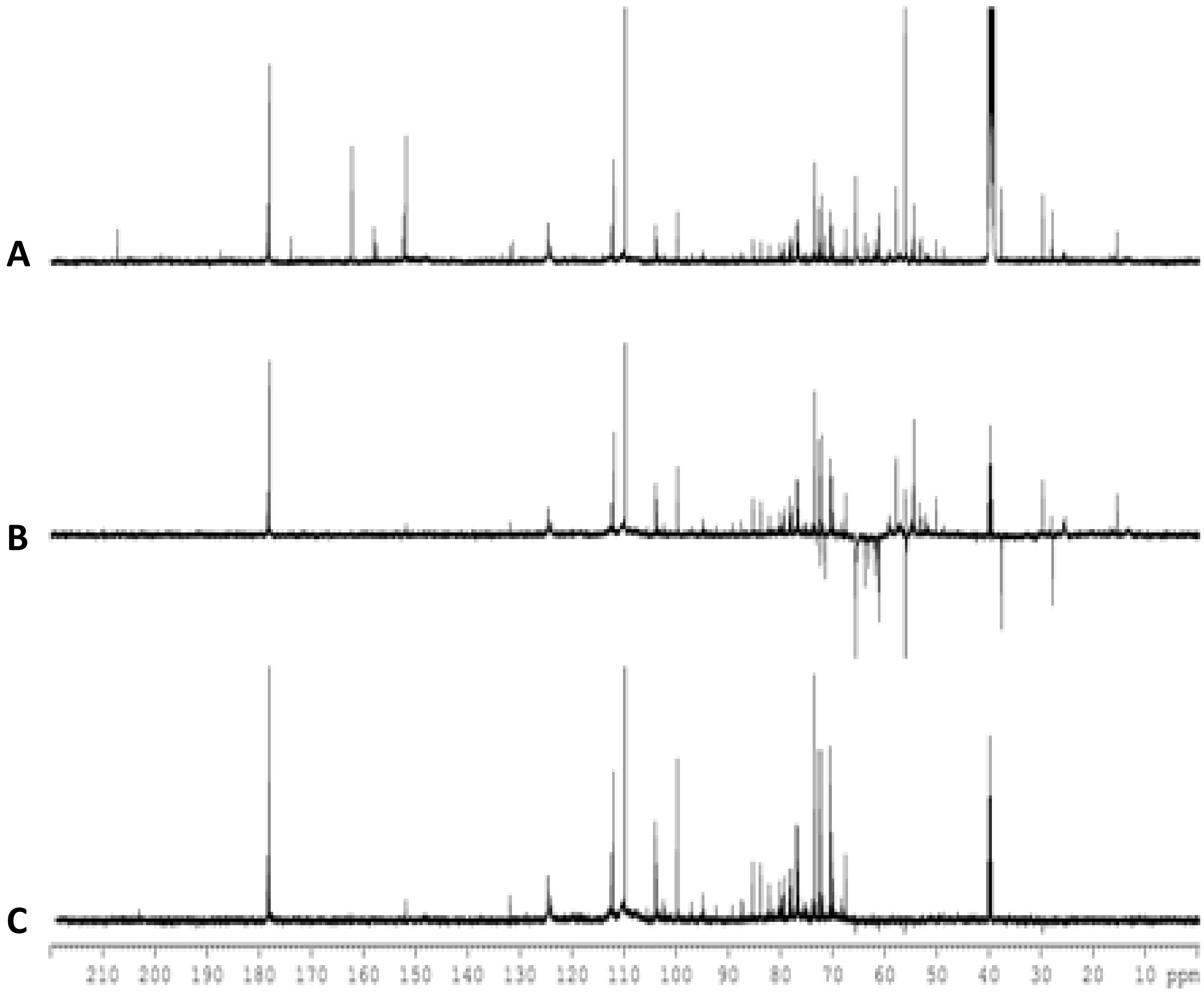

Figure S3b – ^{13}C-NMR spectrum for Humins - A : C13CPD - B : DEPT135 : C : DEPT90

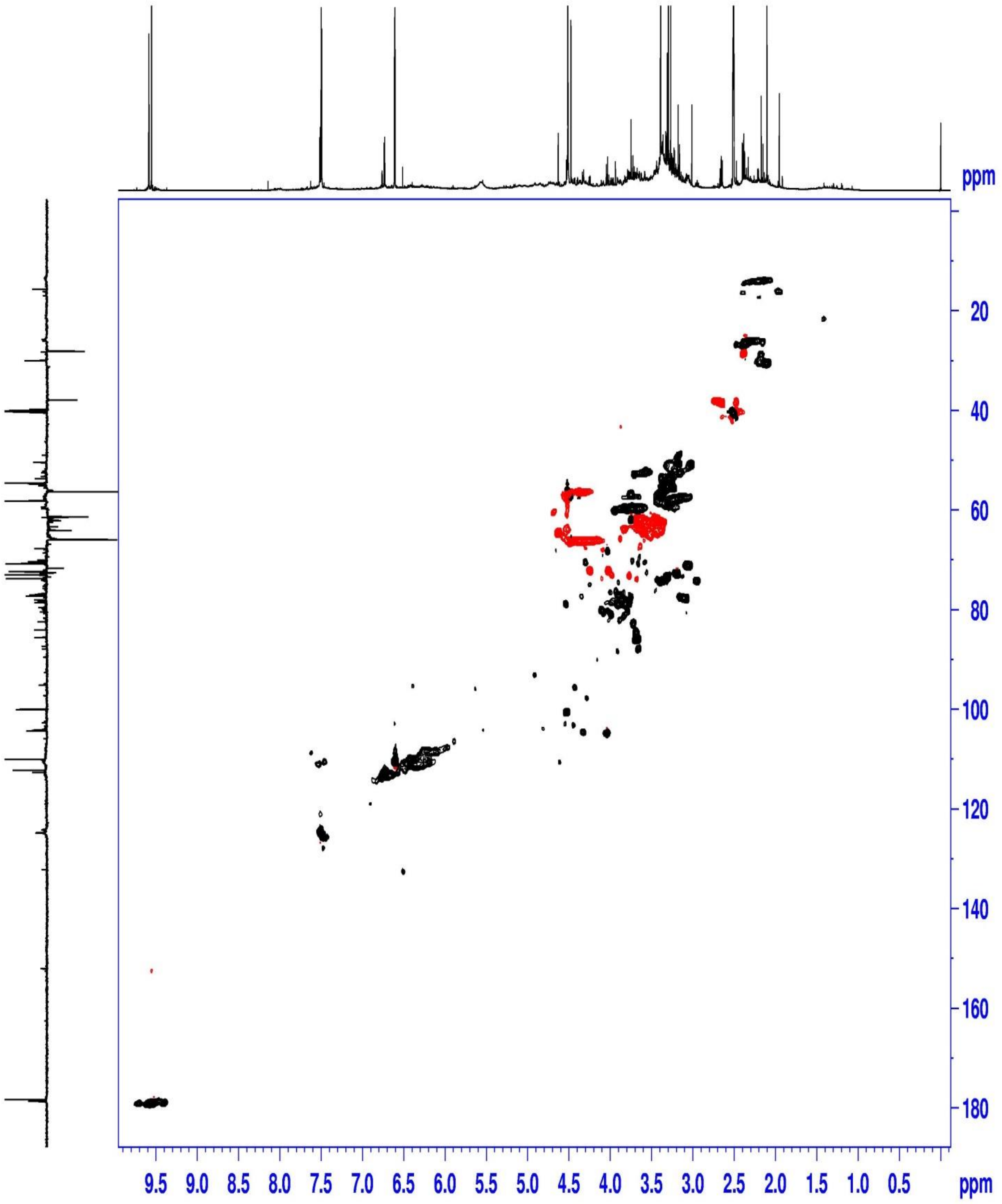

Figure S3c – HSQC spectrum for Humins

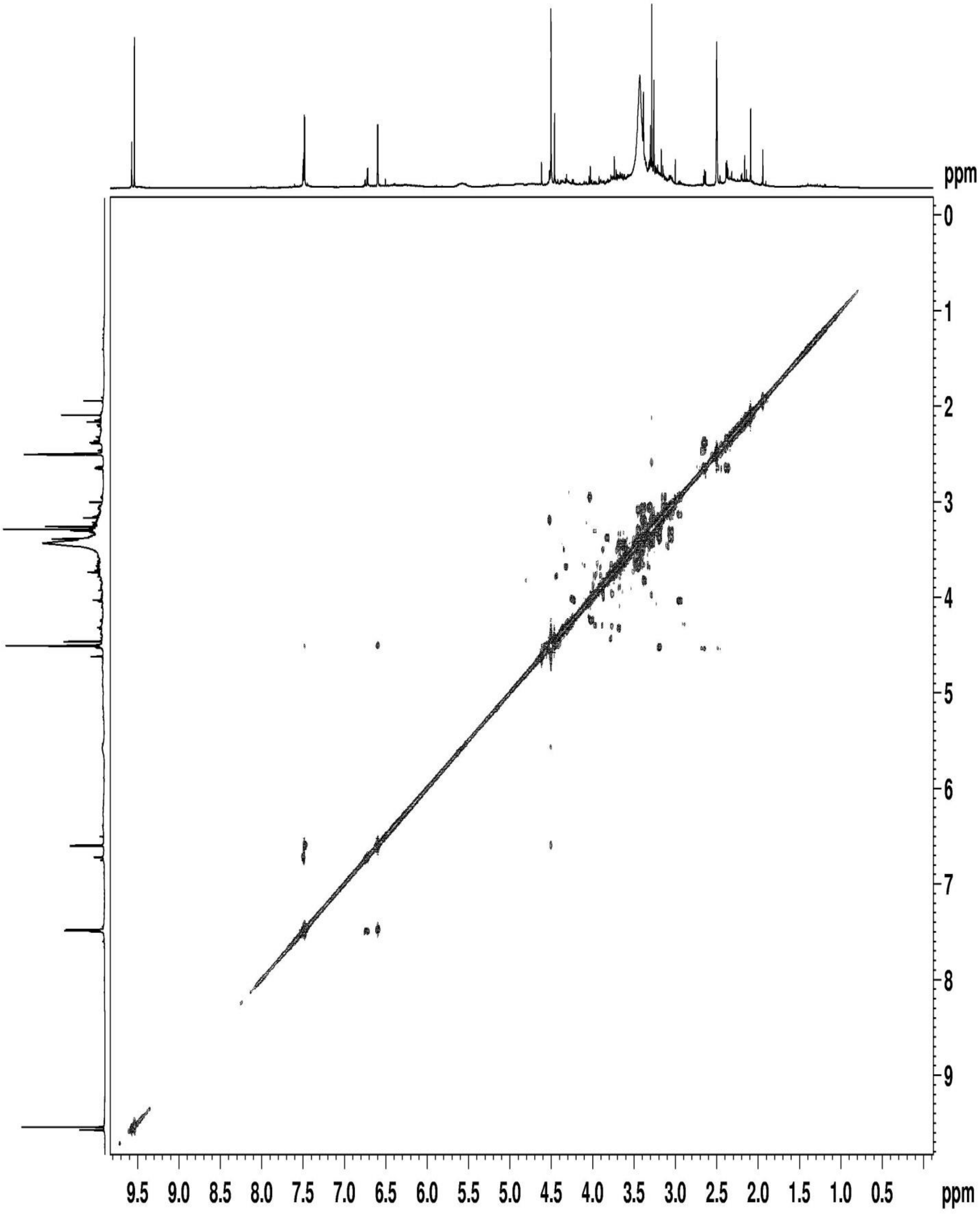

Figure S3d – COSY spectrum for Humins

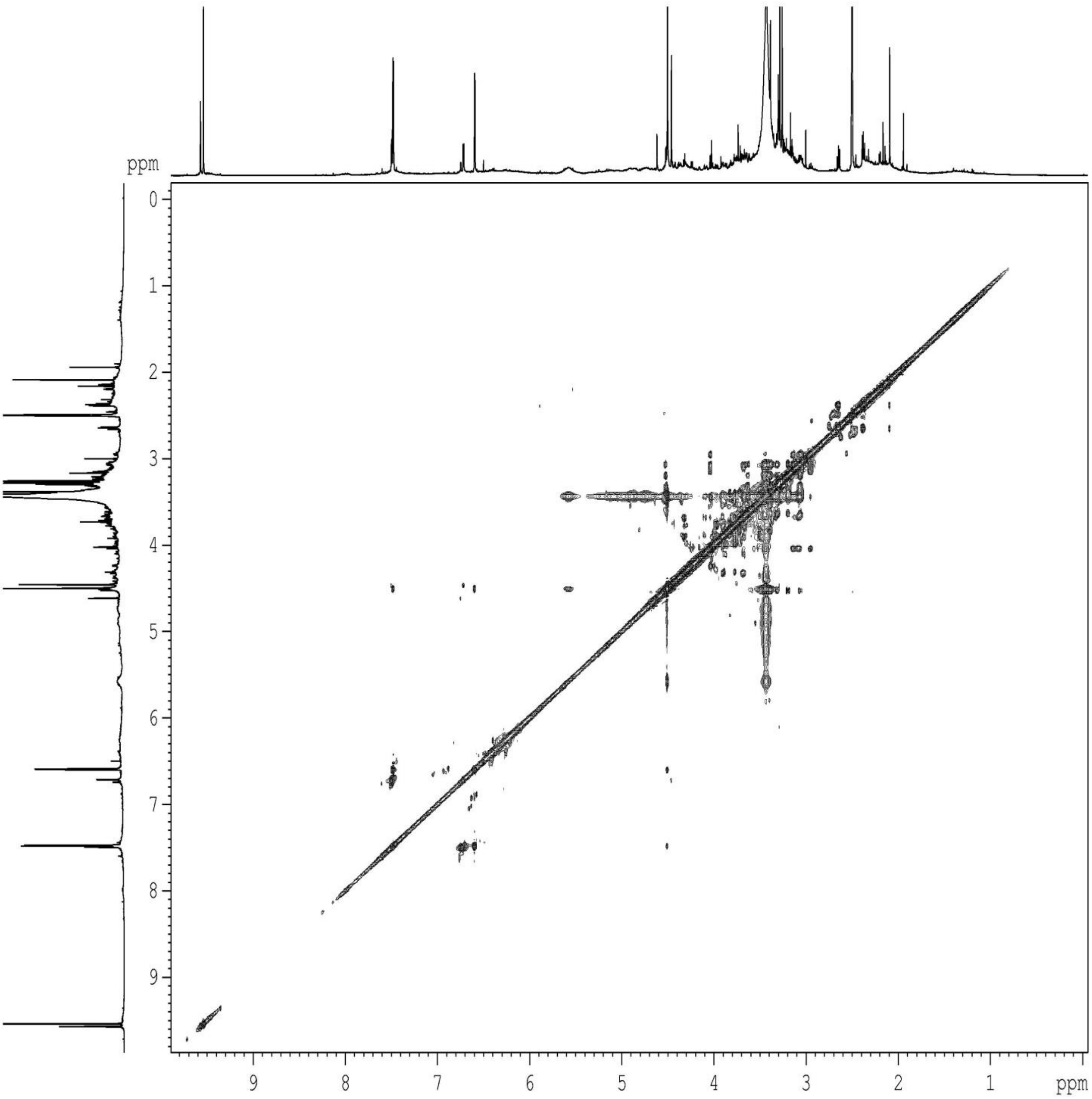

Figure S3e – TOCSY spectrum for Humins

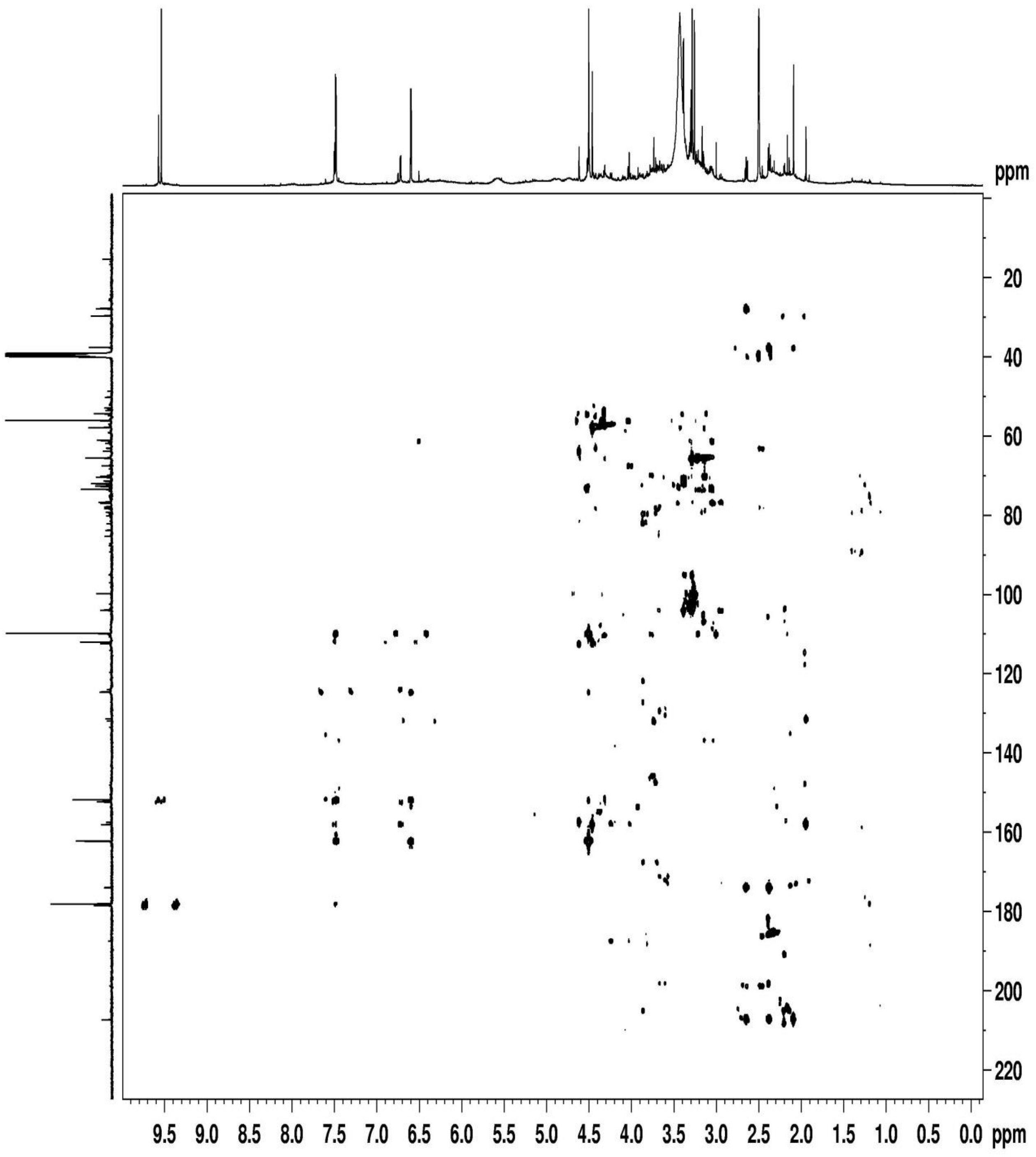

Figure S3f – HMBC spectrum for Humins

IV – Humins during 90 H at 353 K

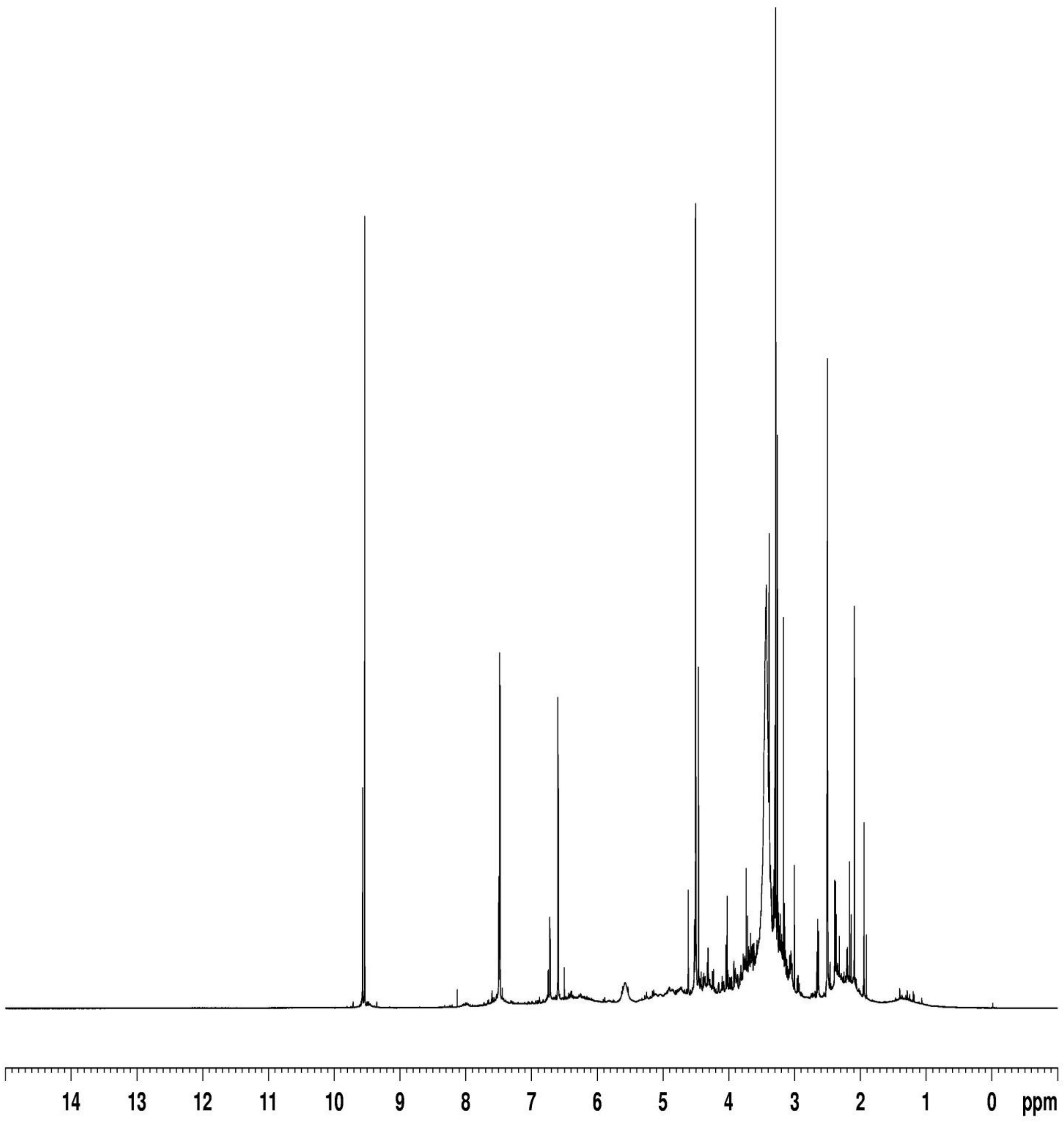

Figure S4a – ^{1}H-NMR spectrum for Humins - 90H at 353K

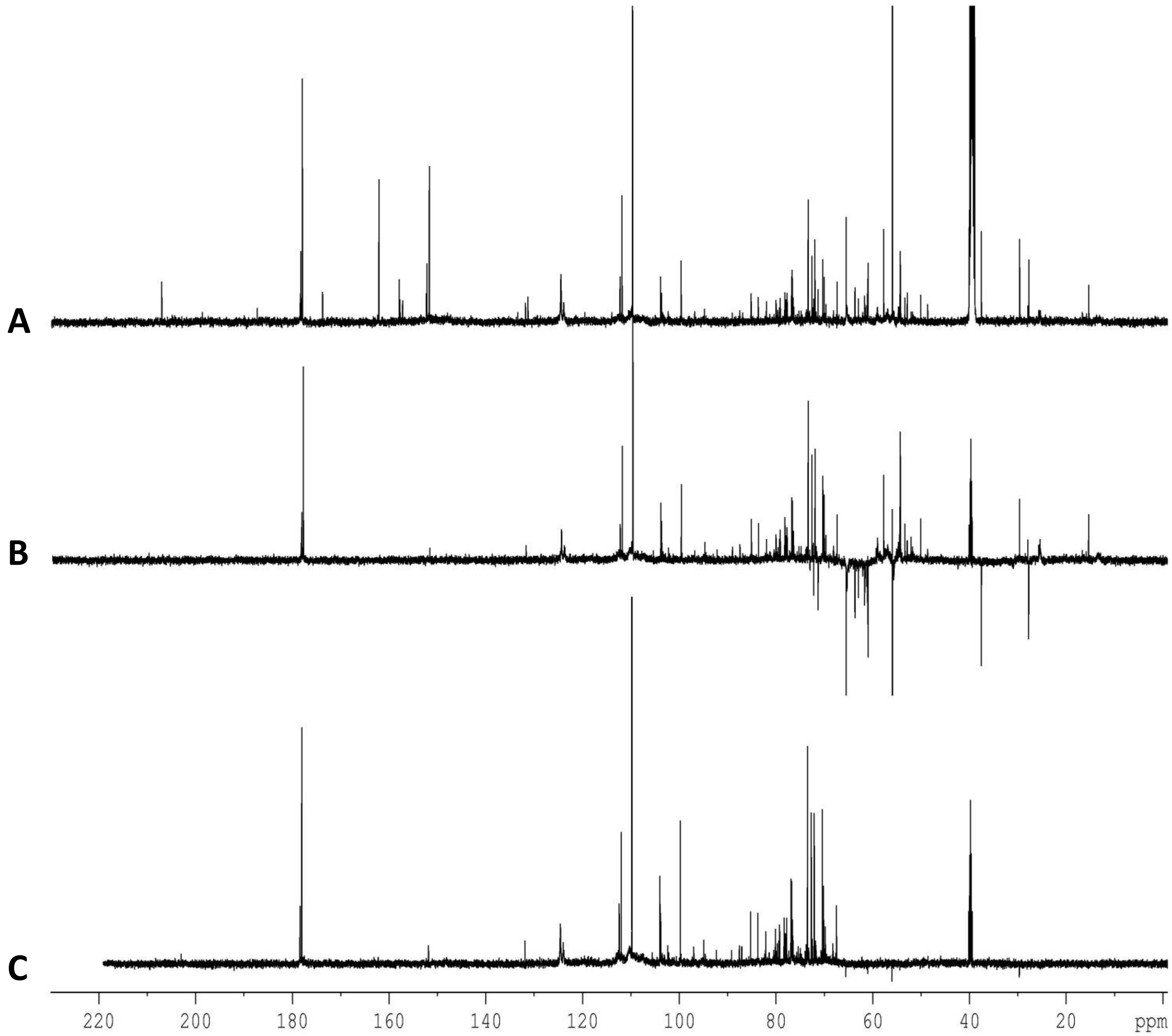

Figure S4b – ^{13}C-NMR spectrum for Humins after 90 H at 353 K - A : C13CPD - B : DEPT135 : C : DEPT90

V – ELO after 90 H at 353 K

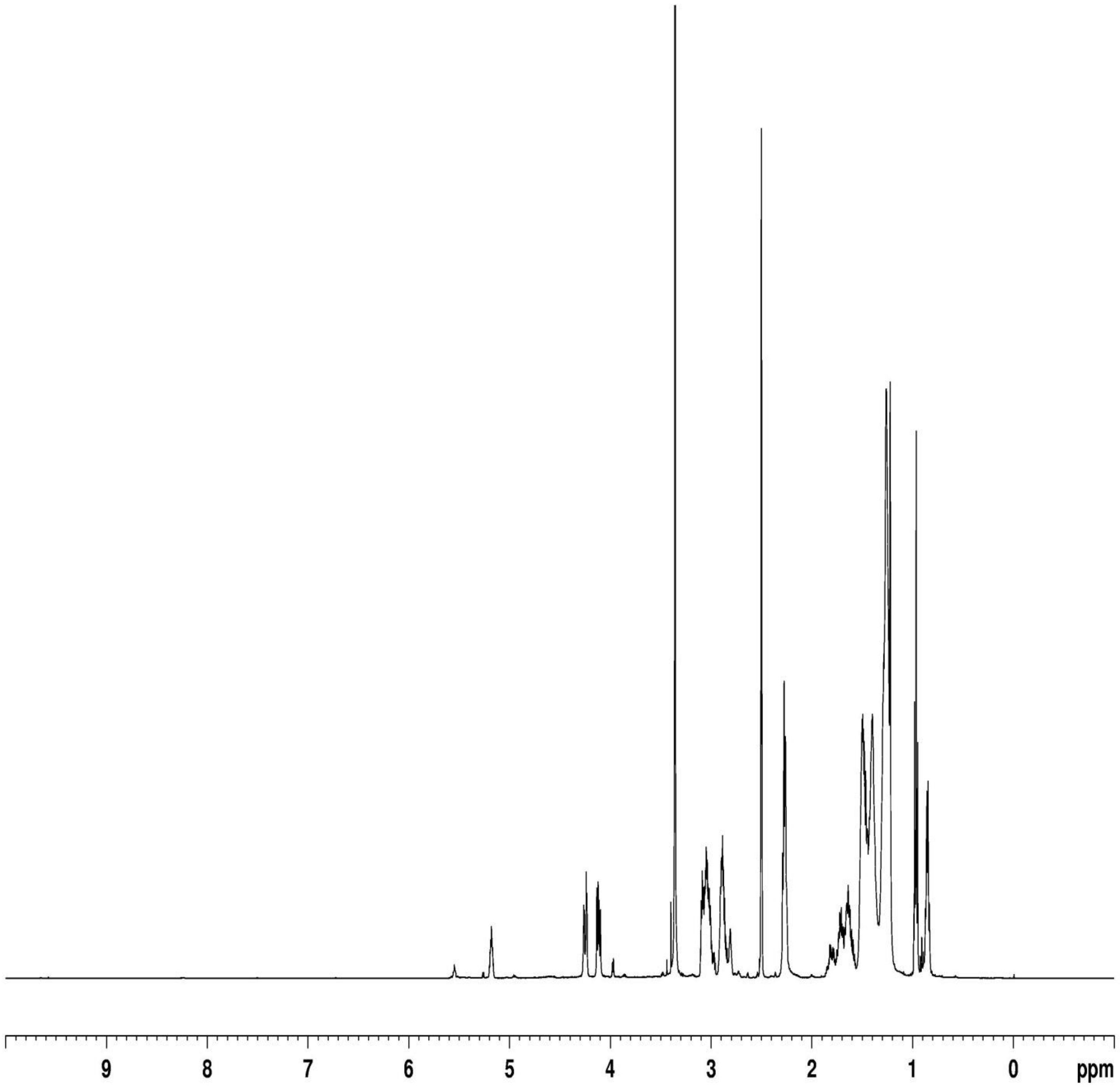

Figure S5a – ^{1}H-NMR spectrum for ELO - 90H at 353K

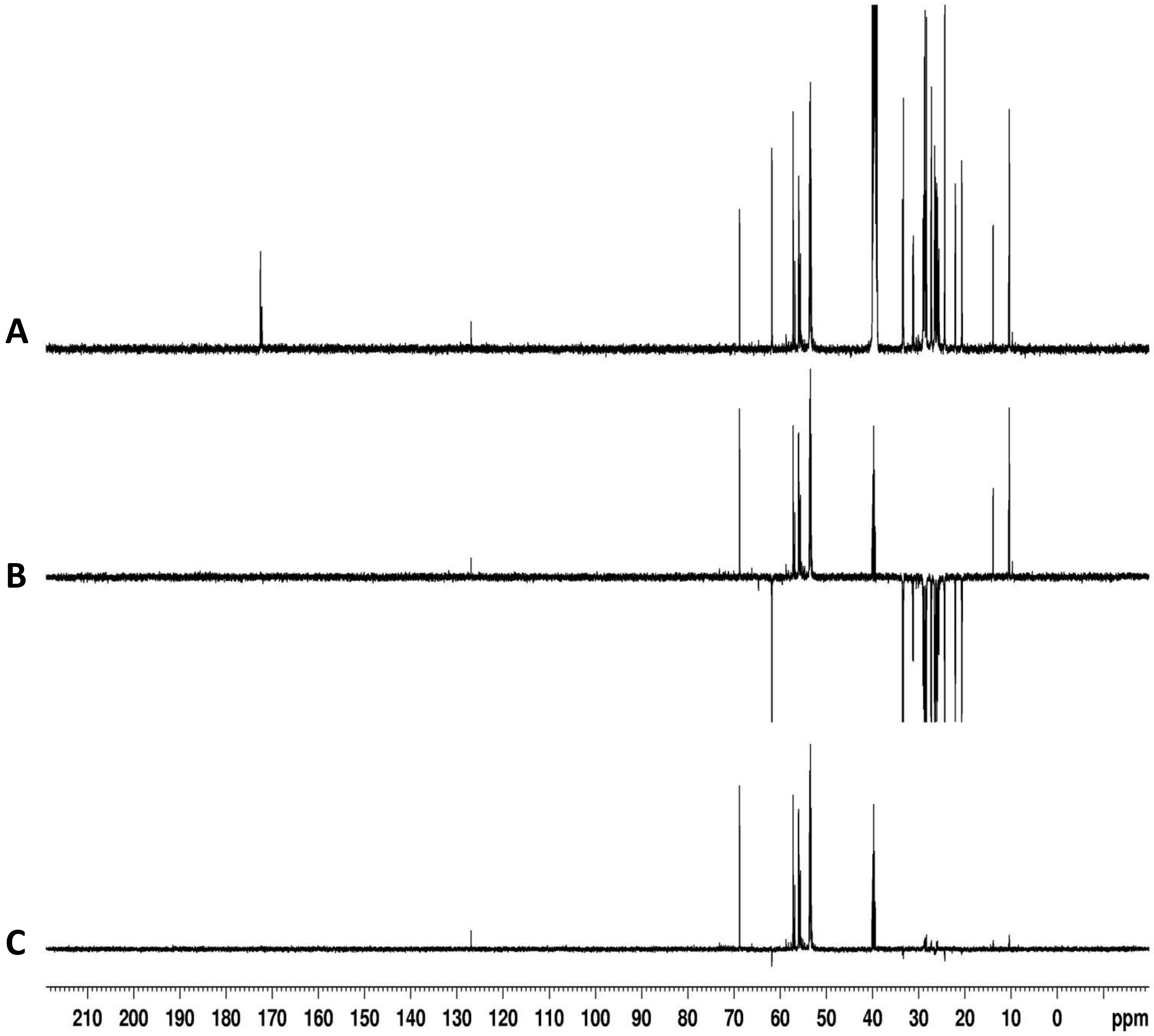

Figure S5b – ^{13}C-NMR spectrum for ELO after 90 H at 353 K - A : C13CPD - B : DEPT135 : C : DEPT90

VI – Capcure after 90 H at 353 K

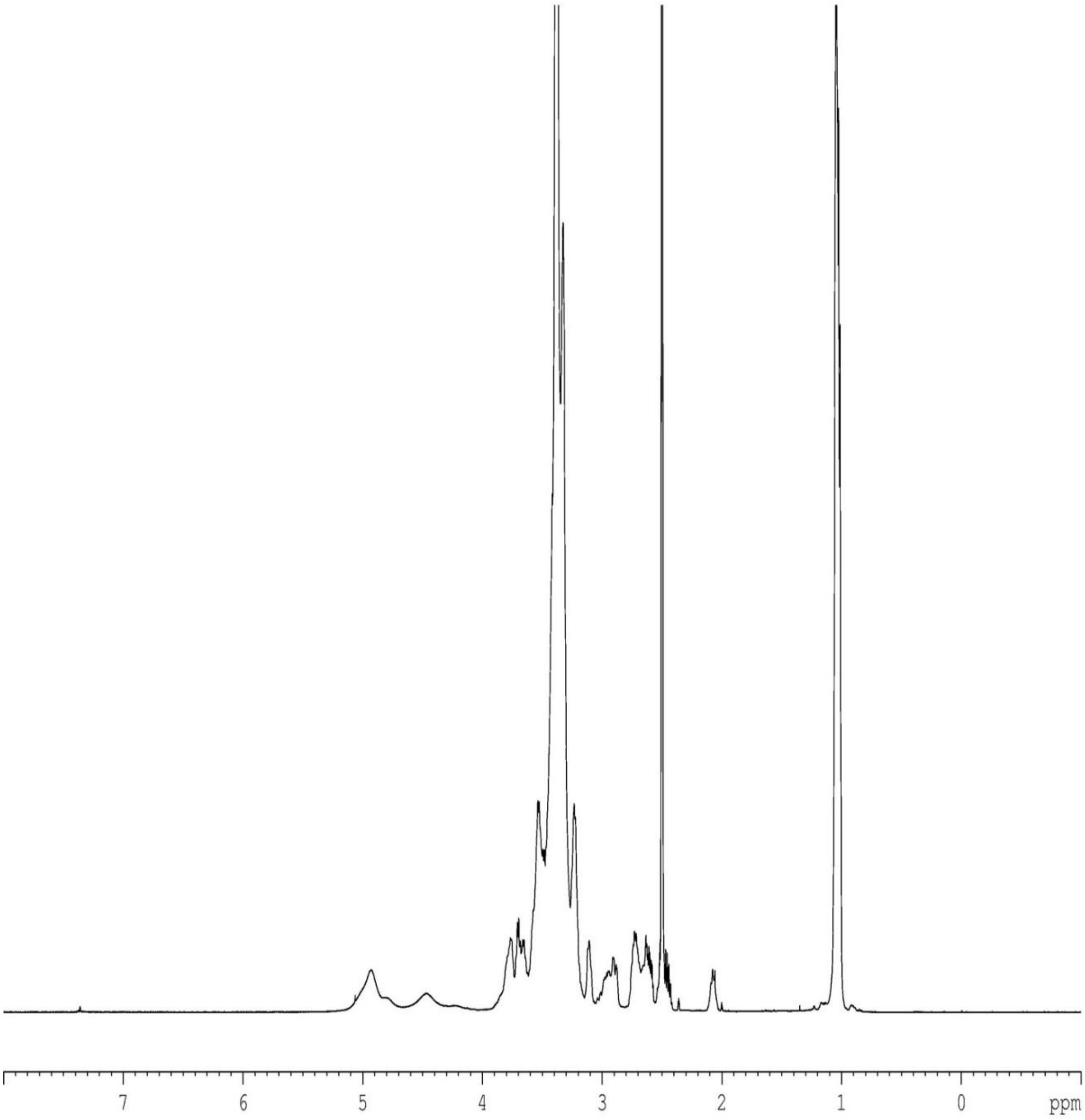

Figure S6a – ^{1}H-NMR spectrum for Capcure after 90 H at 353 K

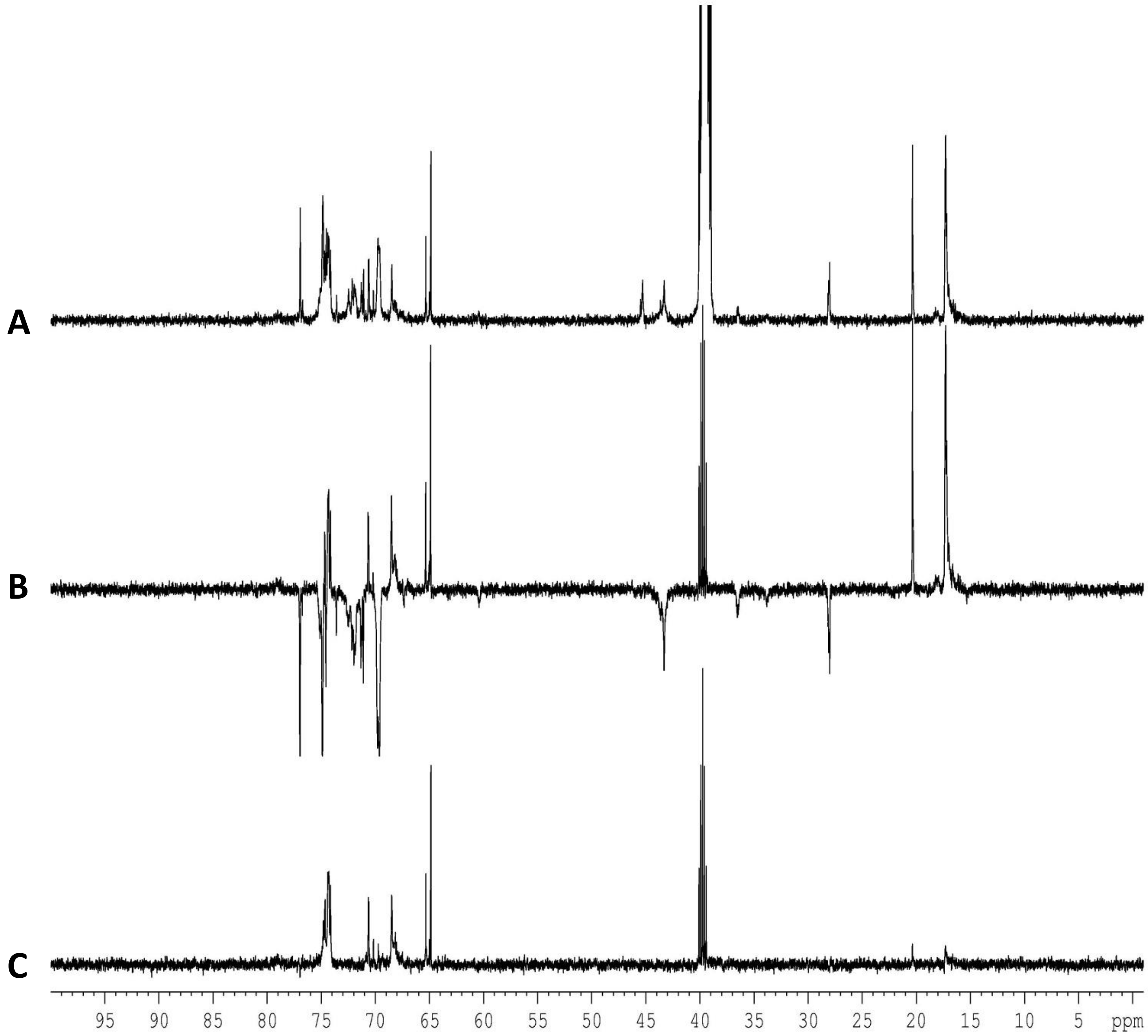

Figure S6b – ^{13}C-NMR spectrum for Capcure after 90 H at 353 K - A : C13CPD - B : DEPT135 : C : DEPT90

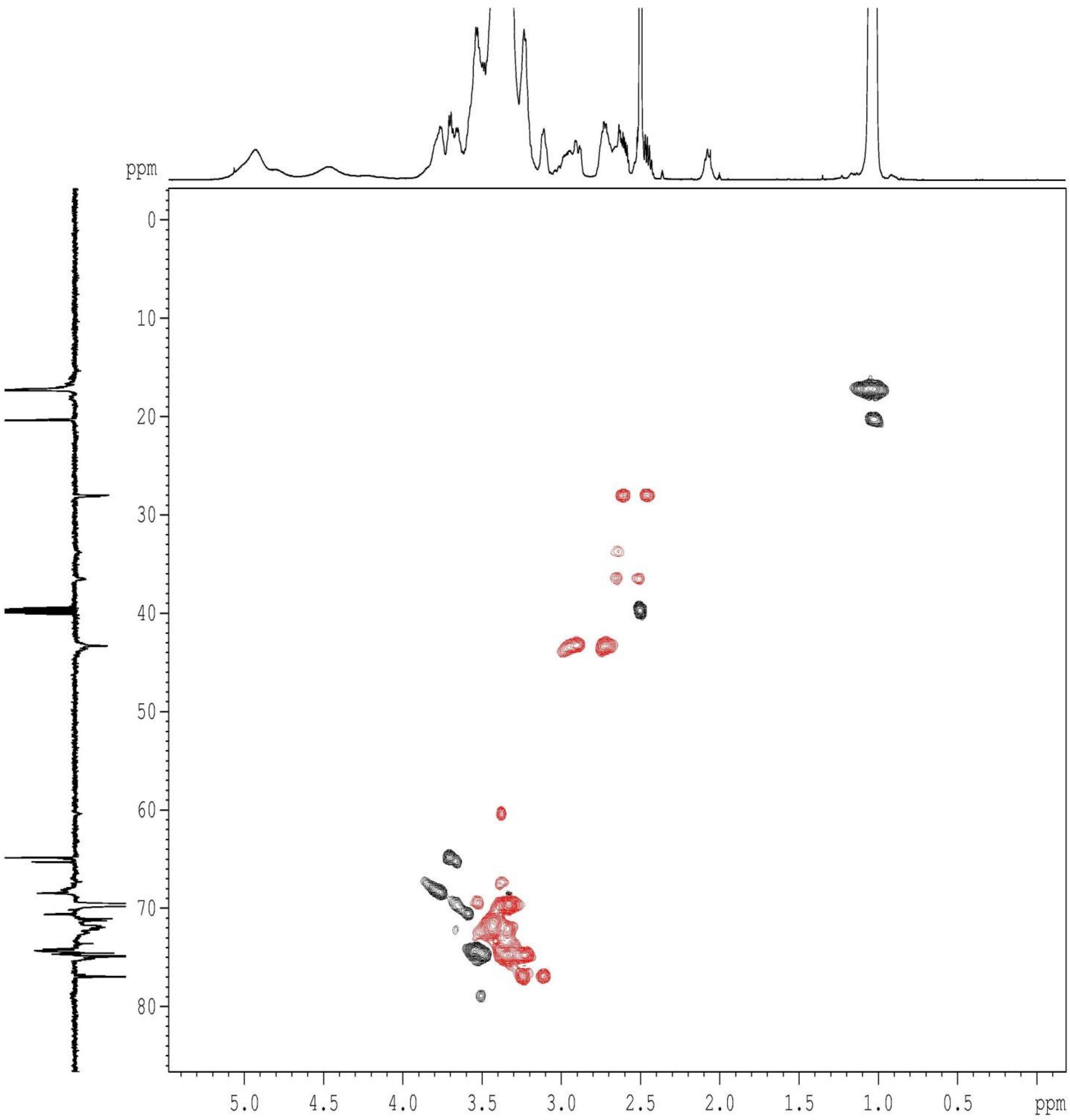

Figure S6c – HSQC spectrum for Capcure after 90 H at 353 K

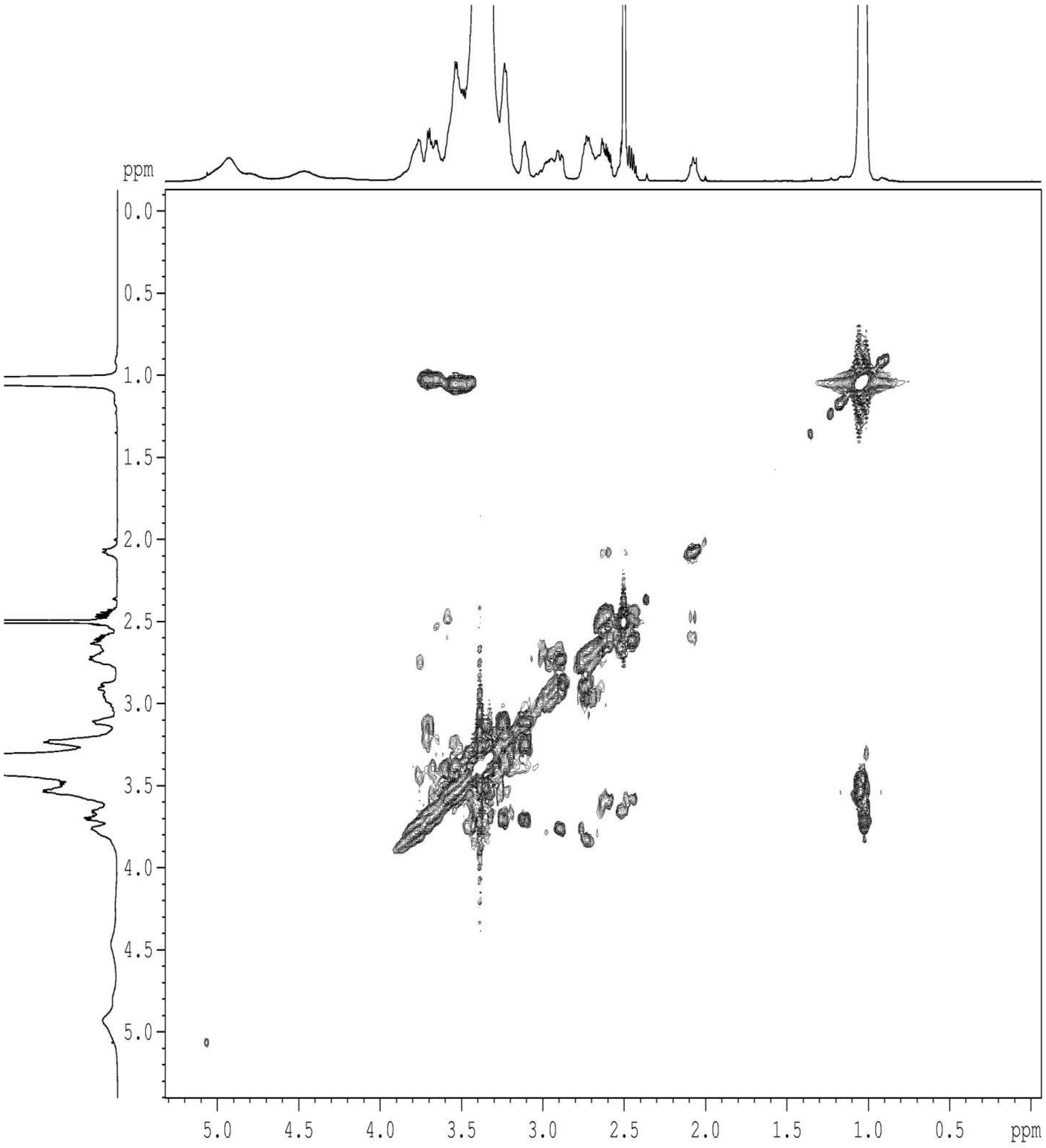

Figure S6d – COSY spectrum for Capcure - 90H at 353K

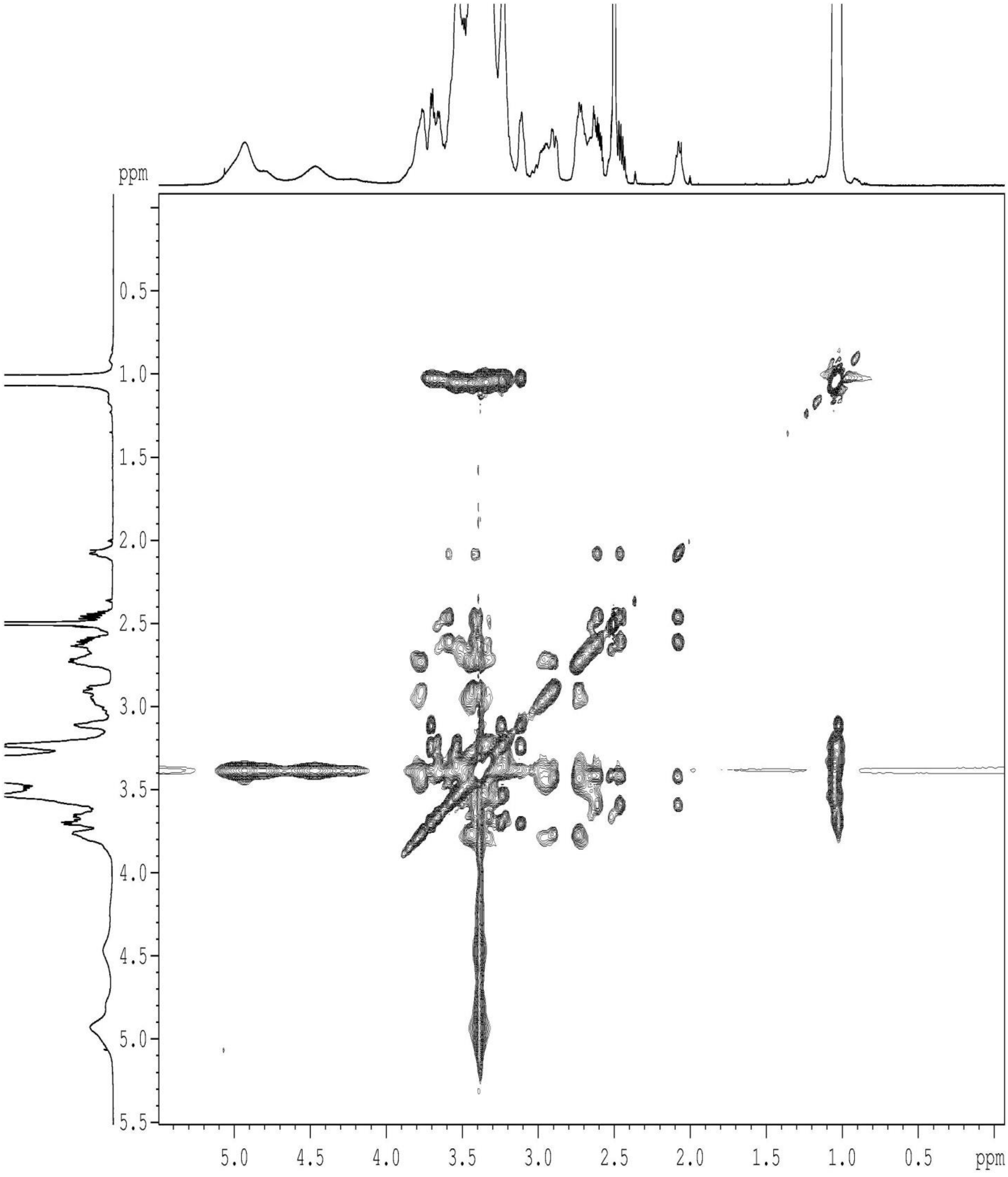

Figure S6e – TOCSY spectrum for Capcure after 90 H at 353 K

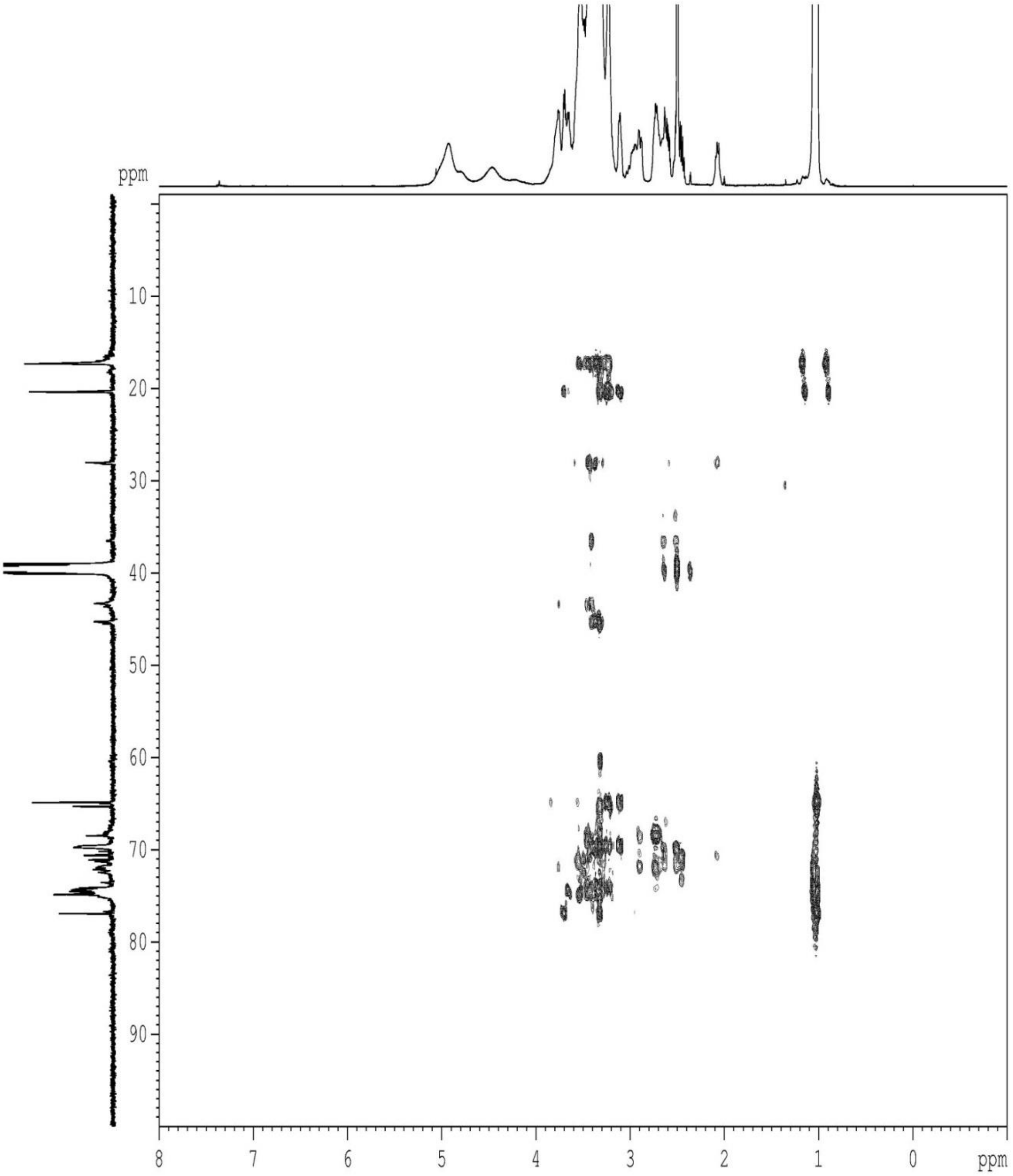

Figure S6f – HMBC spectrum for Capcure after 90 H at 353 K

VII - Capcure + ELO after 90 H at 353 K

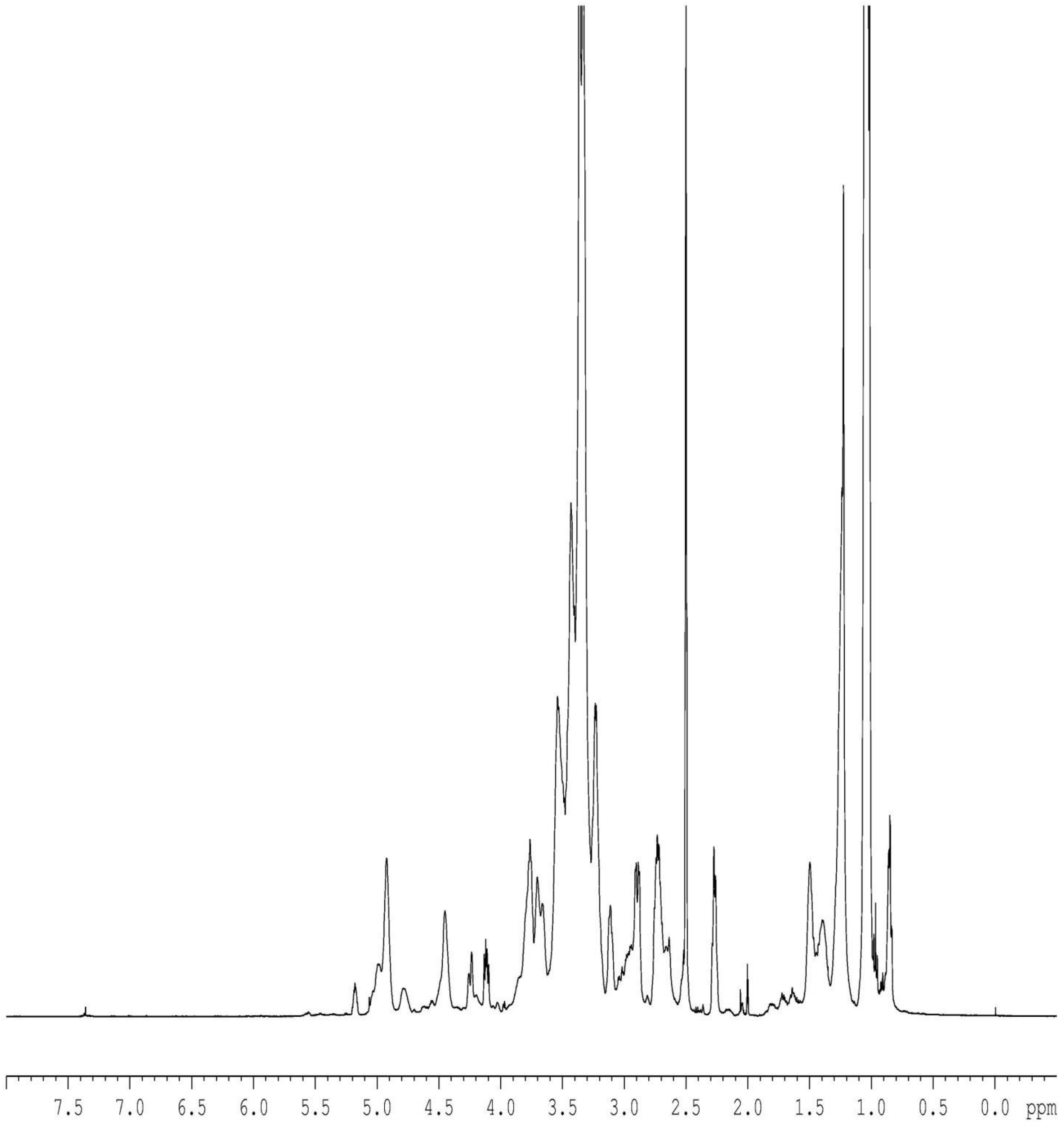

Figure S7a – ^{1}H-NMR spectrum for Capcure + ELO after 90 H at 353 K

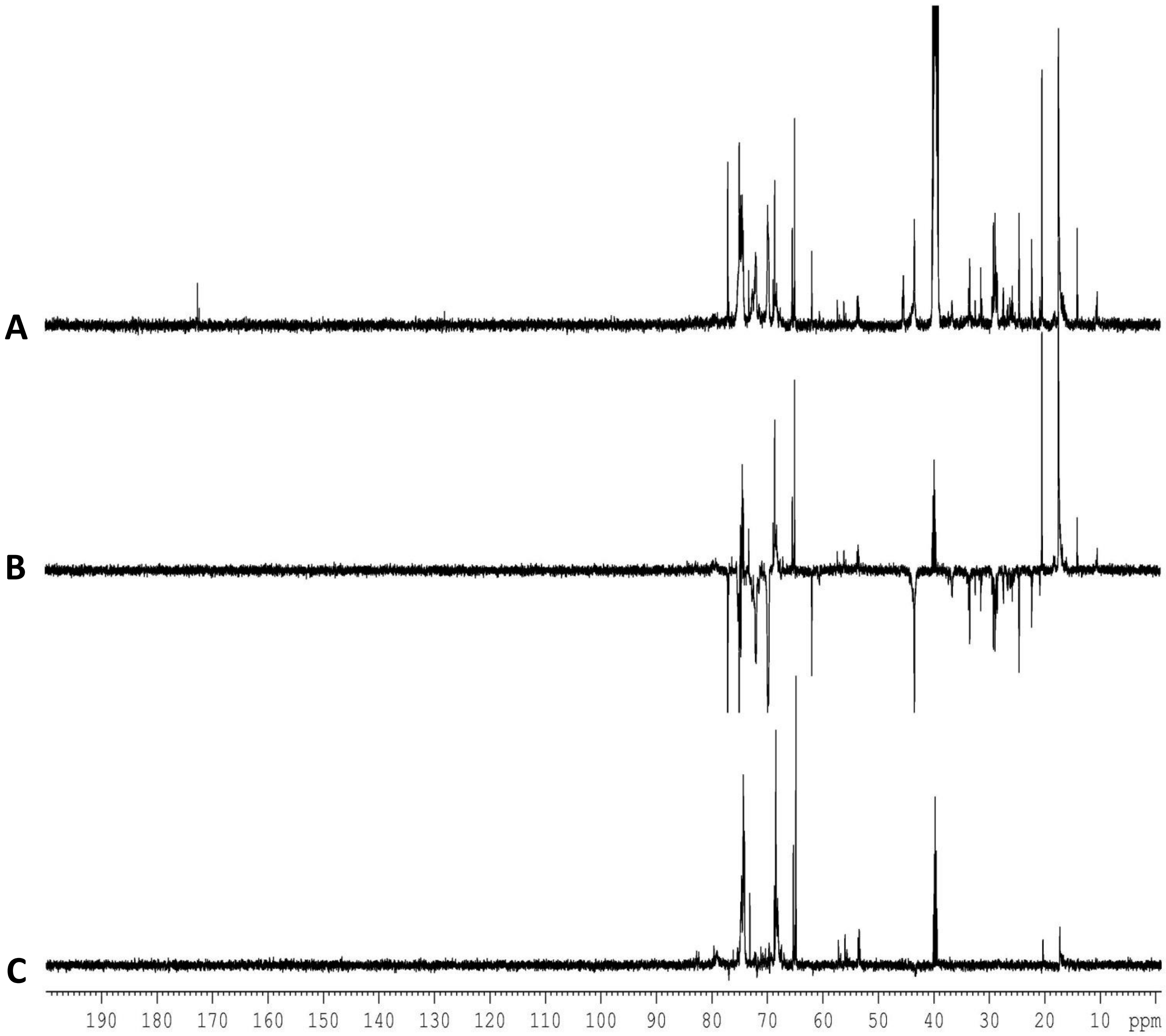

Figure S7b – ^{13}C-NMR spectrum for Capcure + ELO after 90 H at 353 K - A : C13CPD - B : DEPT135 : C : DEPT90

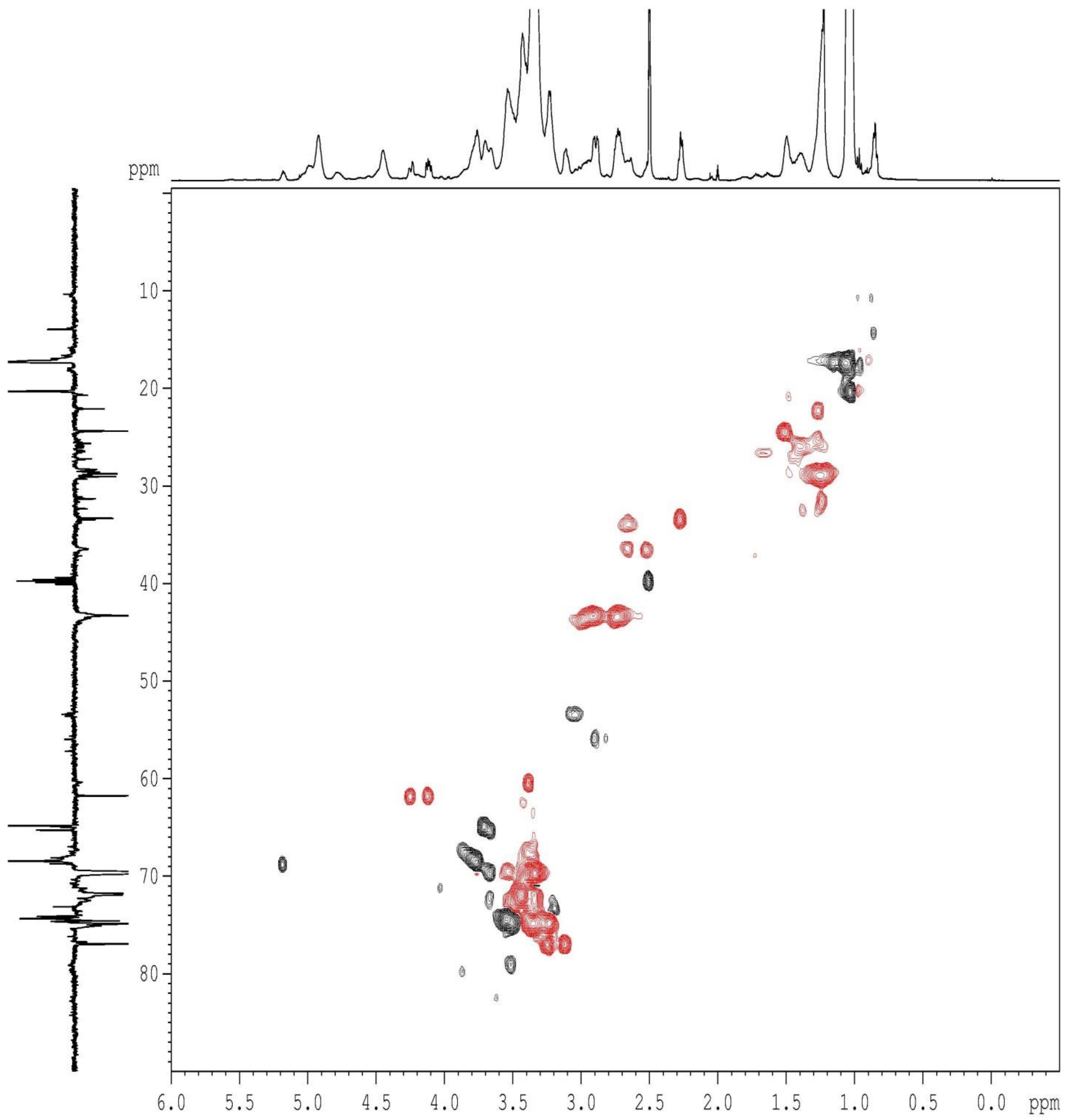

Figure S7c – HSQC spectrum for Capcure + ELO after 90 H at 353 K

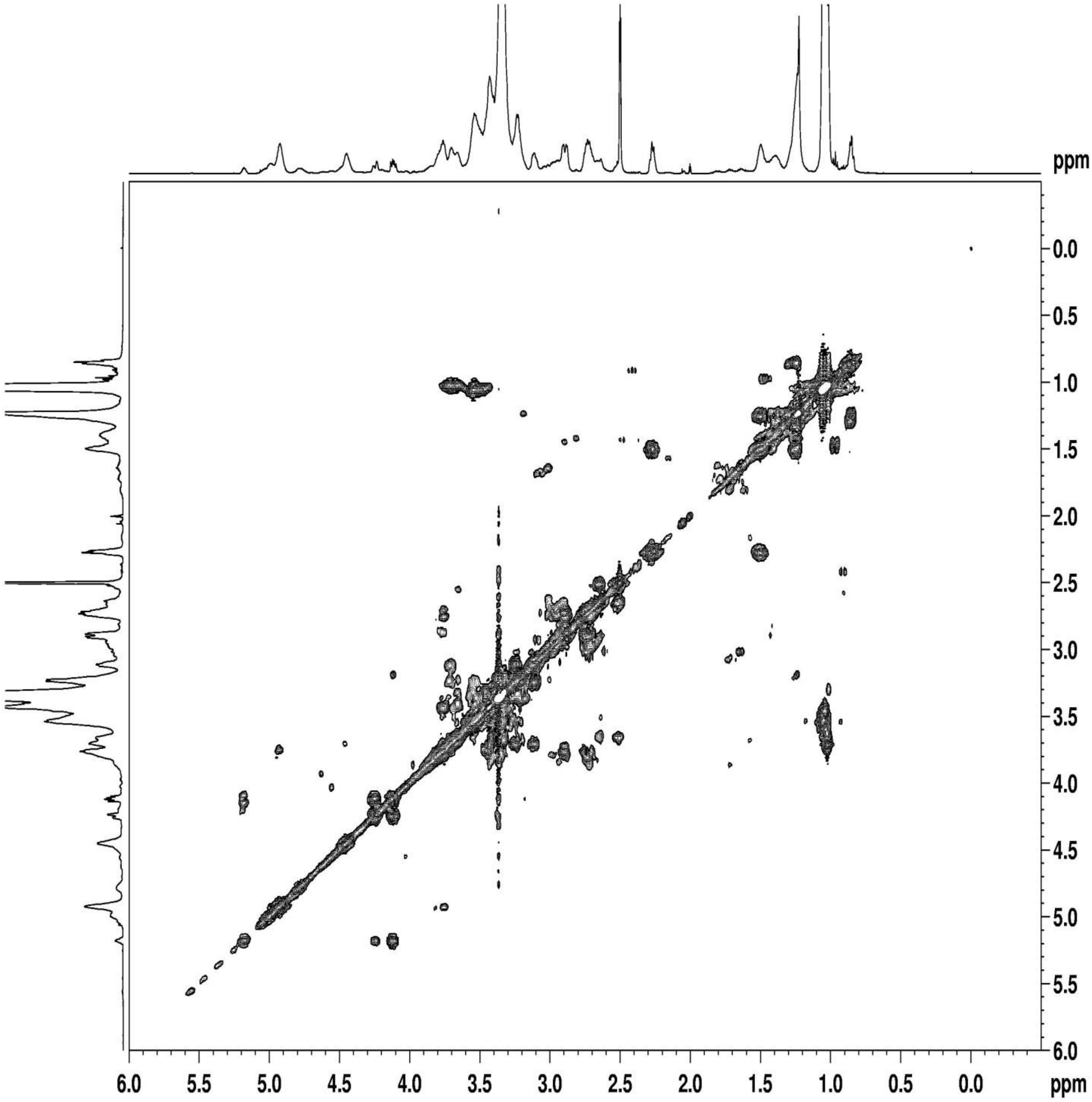

Figure S7d – COSY spectrum for Capcure + ELO after 90 H at 353 K

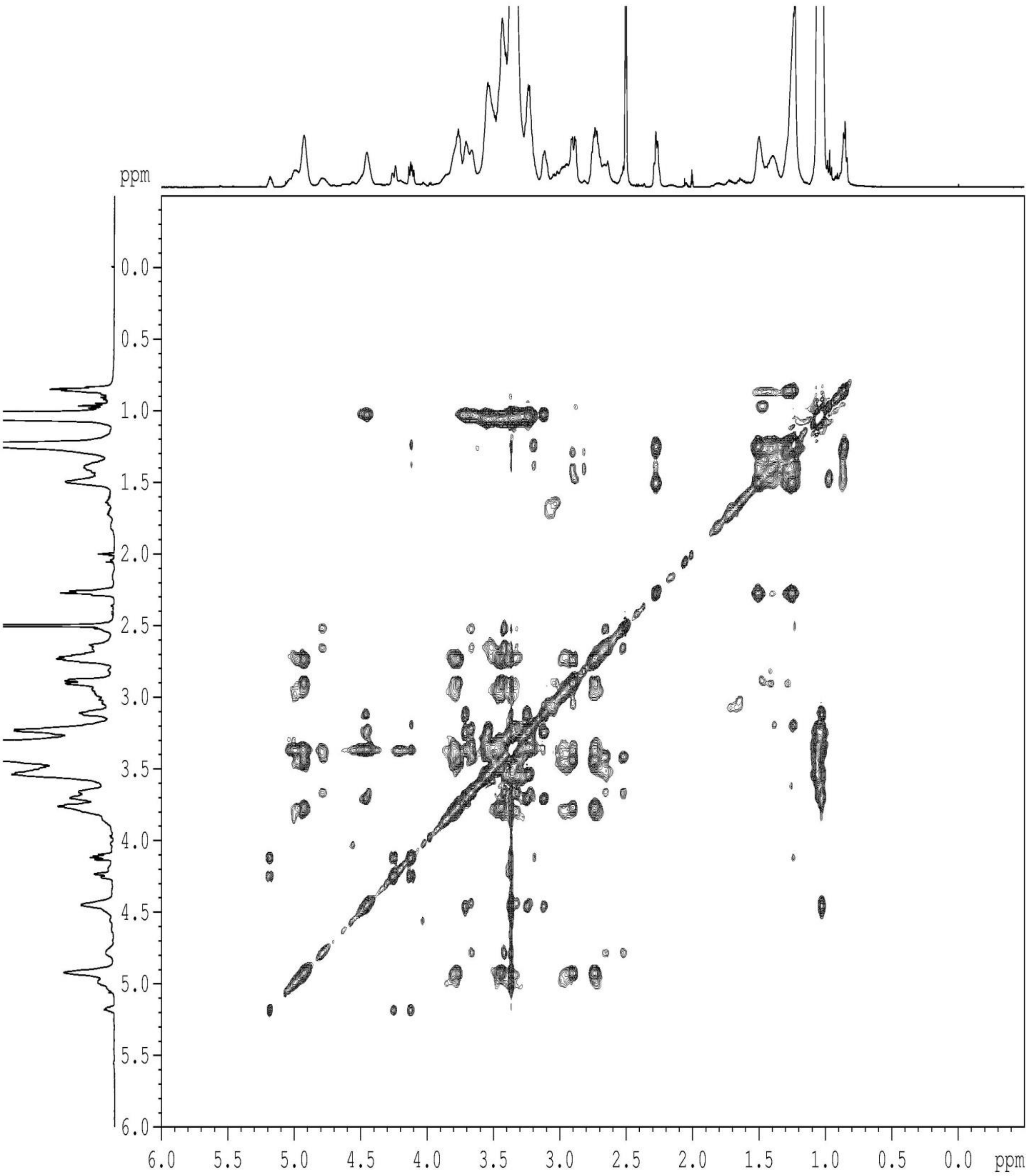

Figure S7e – TOCSY spectrum for Capcure + ELO after 90 H at 353 K

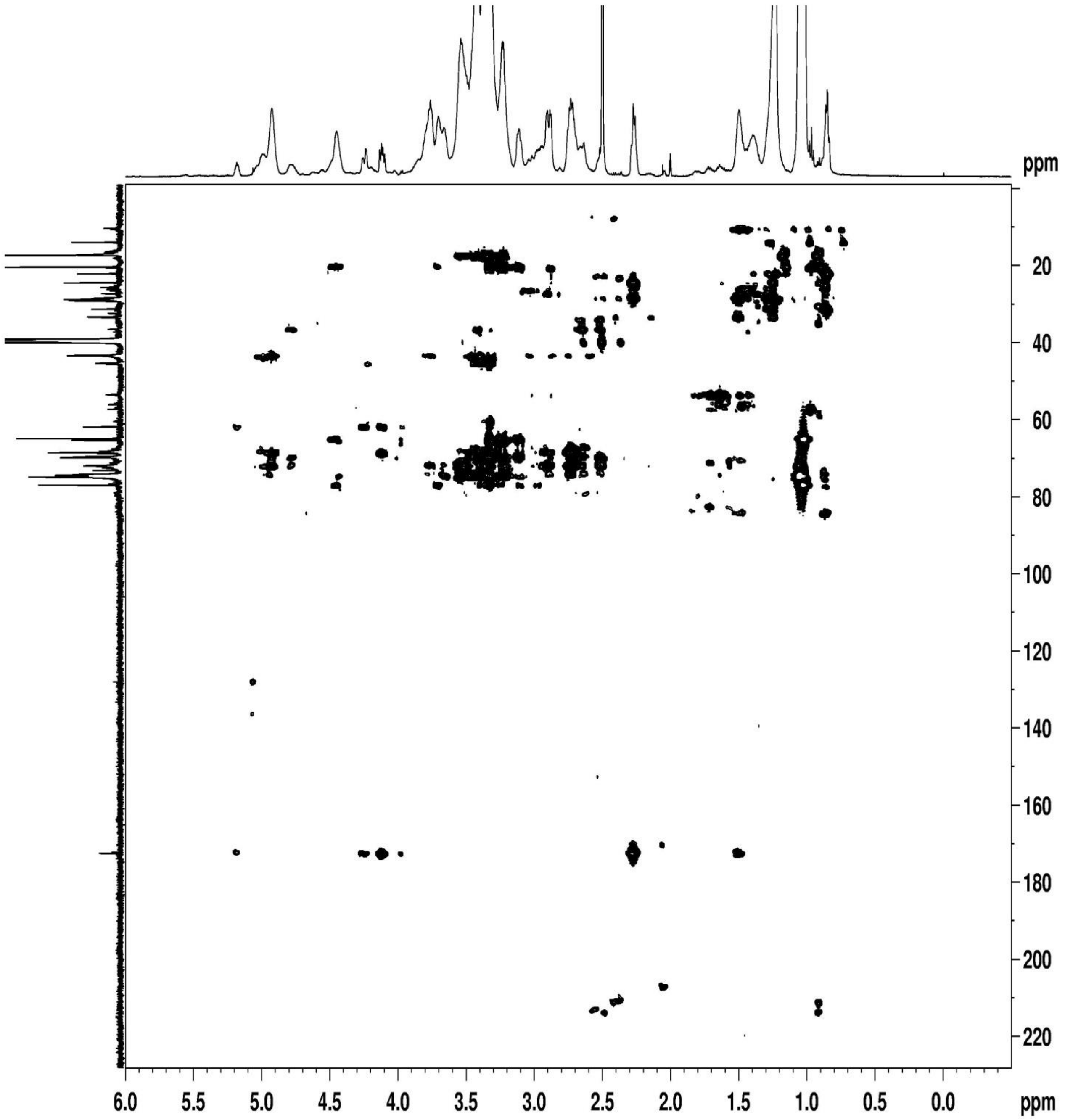

Figure S7f – HMBC spectrum for Capcure + ELO after 90 H at 353 K

VIII - Capcure + Humins after 90 H at 353 K

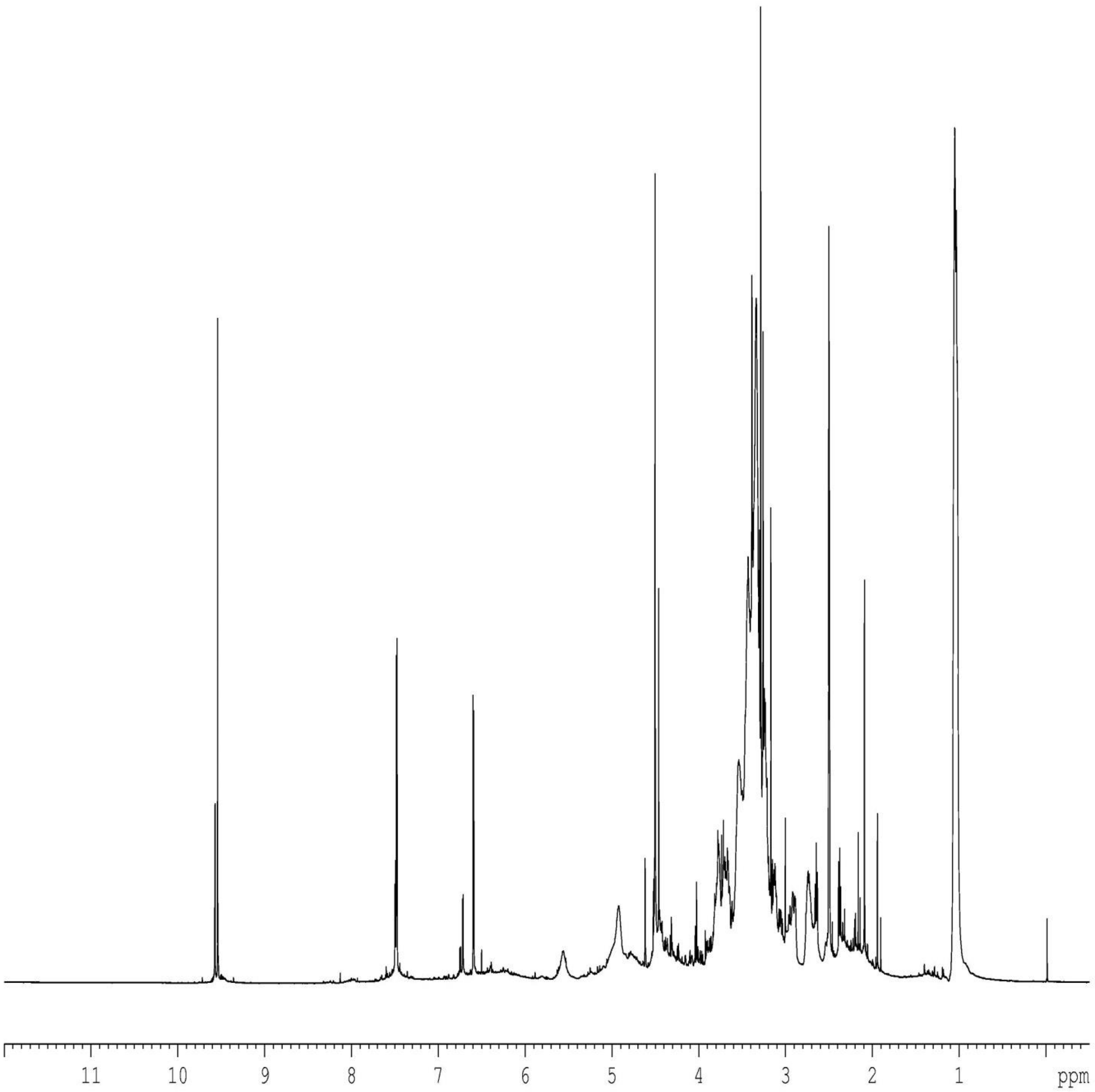

Figure S8a – ^{1}H-NMR spectrum for Capcure + Humins after 90 H at 353 K

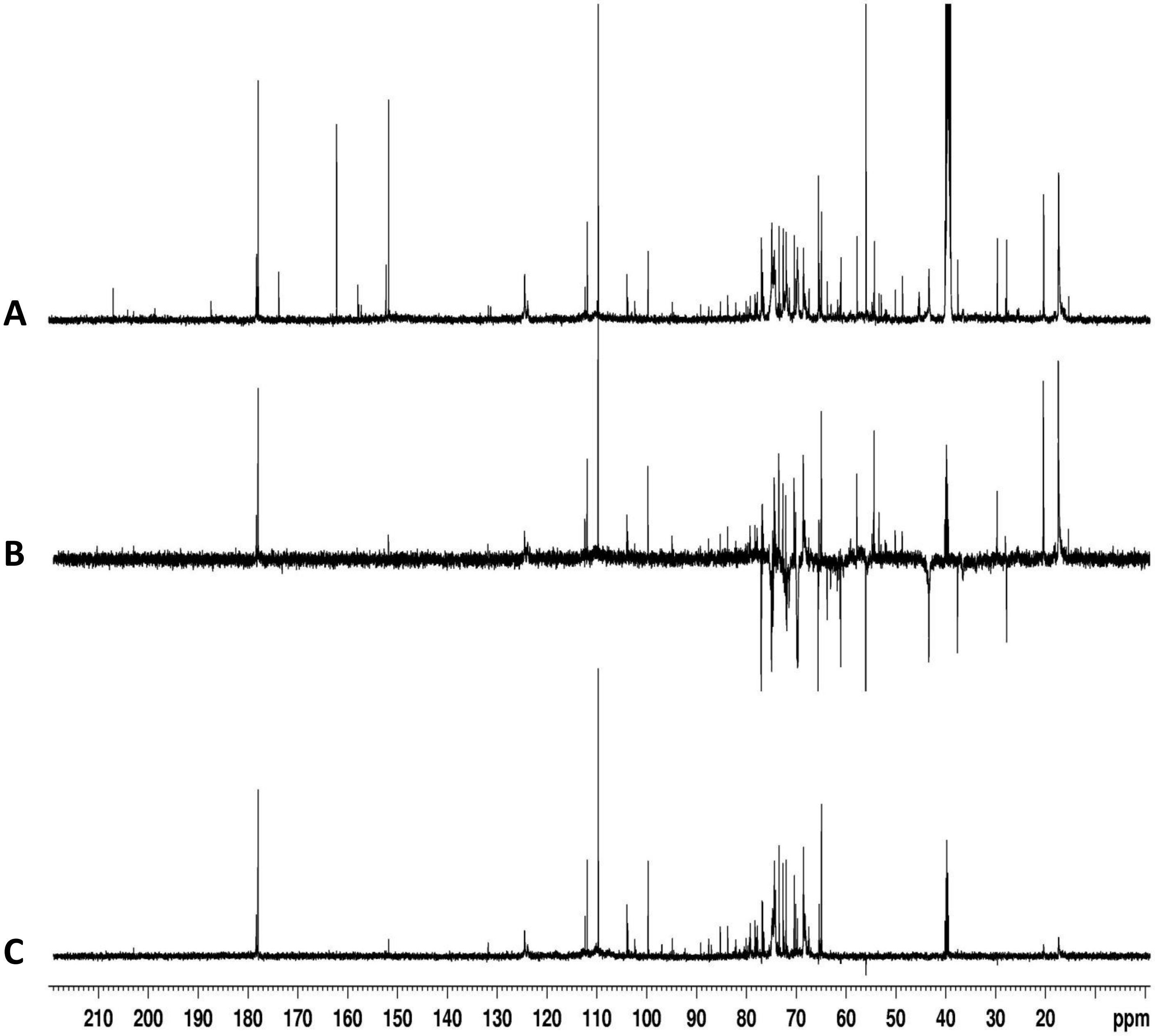

Figure S8b – ^{13}C-NMR spectrum for Capcure + Humins after 90 H at 353 K - A : C13CPD - B : DEPT135 : C : DEPT90

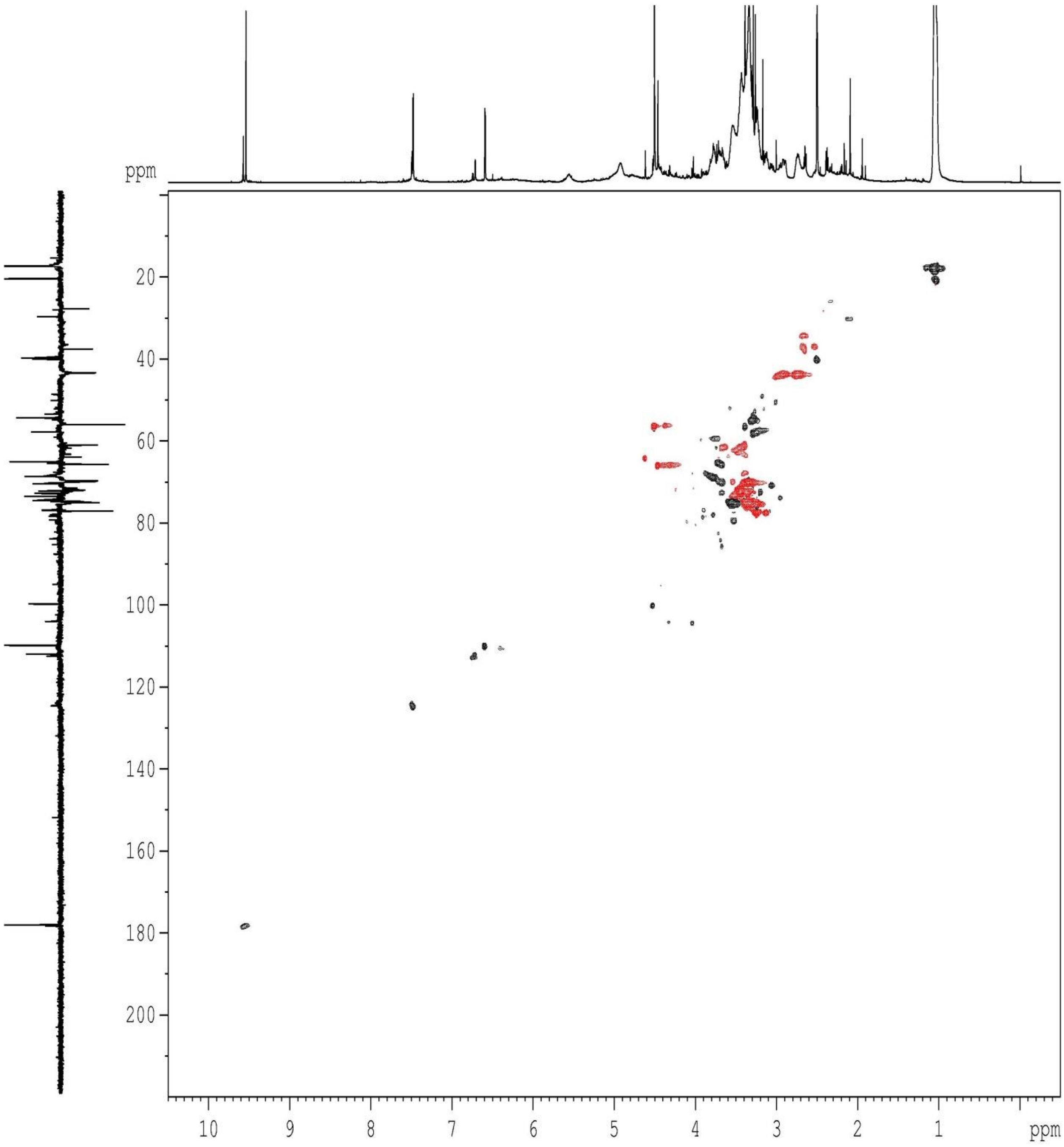

Figure S8c – HSQC spectrum for Capcure + Humins after 90 H at 353 K

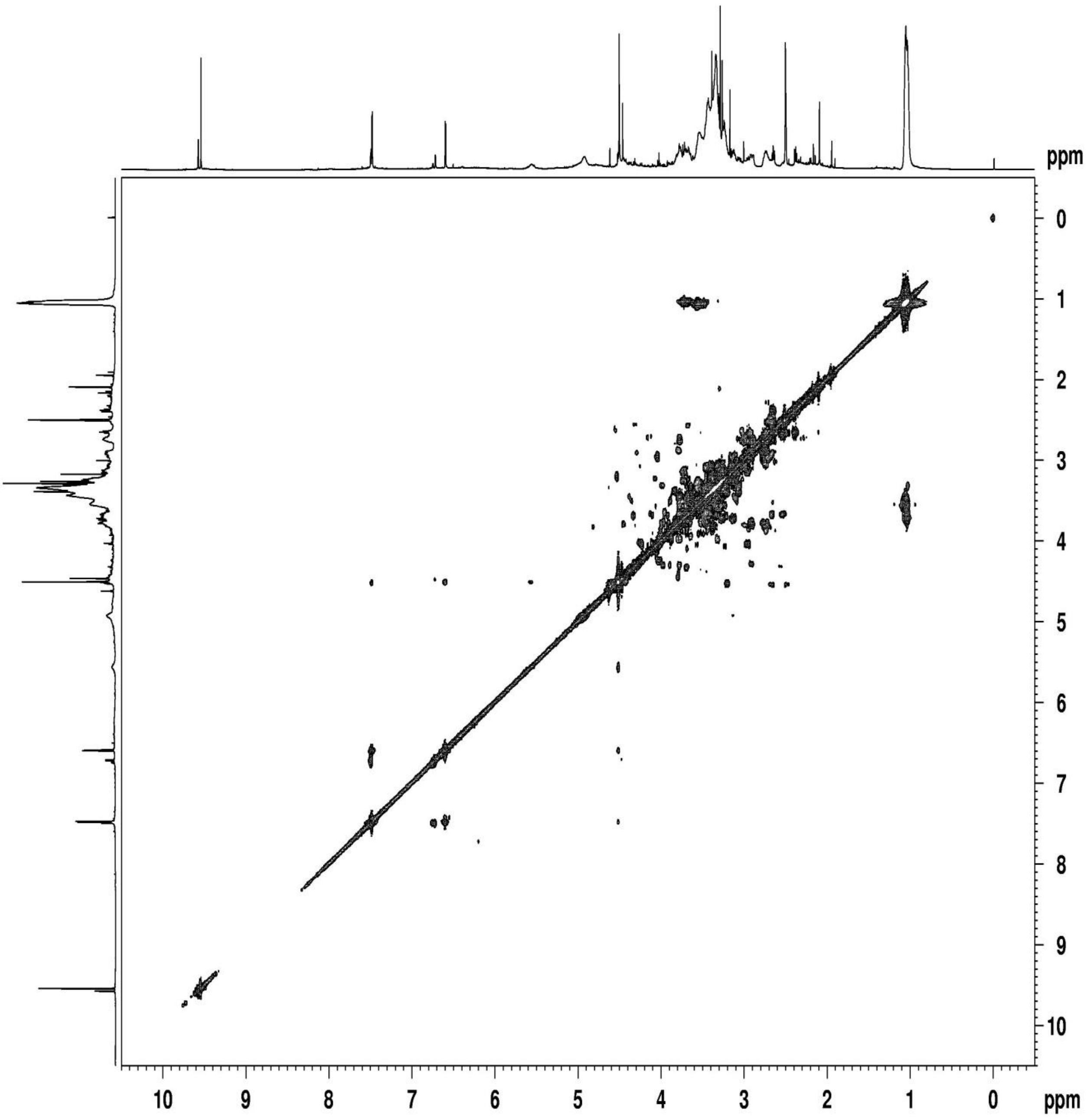

Figure S8d – COSY spectrum for Capcure + Humins after 90 H at 353 K

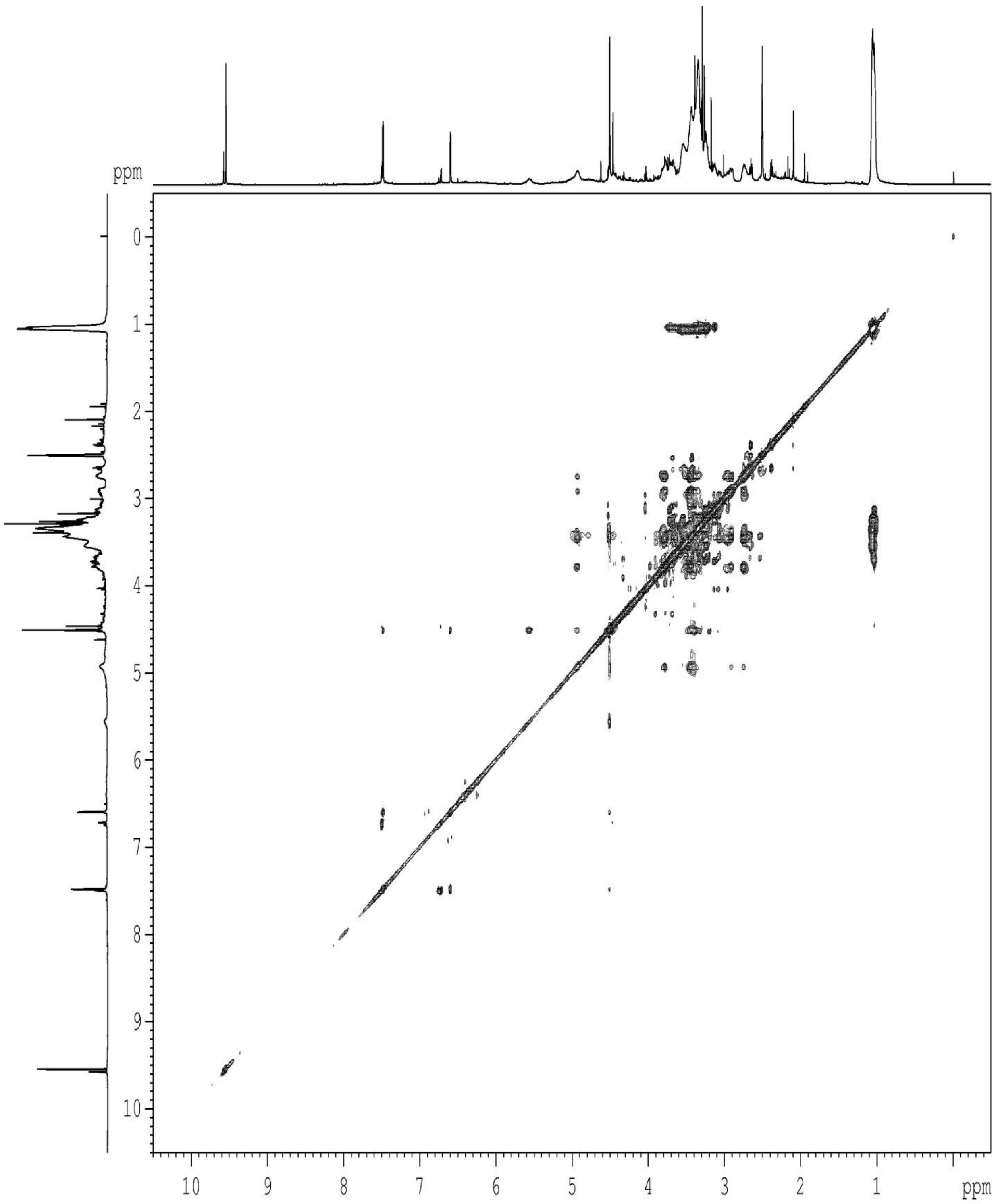

Figure S8e – TOCSY spectrum for Capcure + Humins after 90 H at 353 K

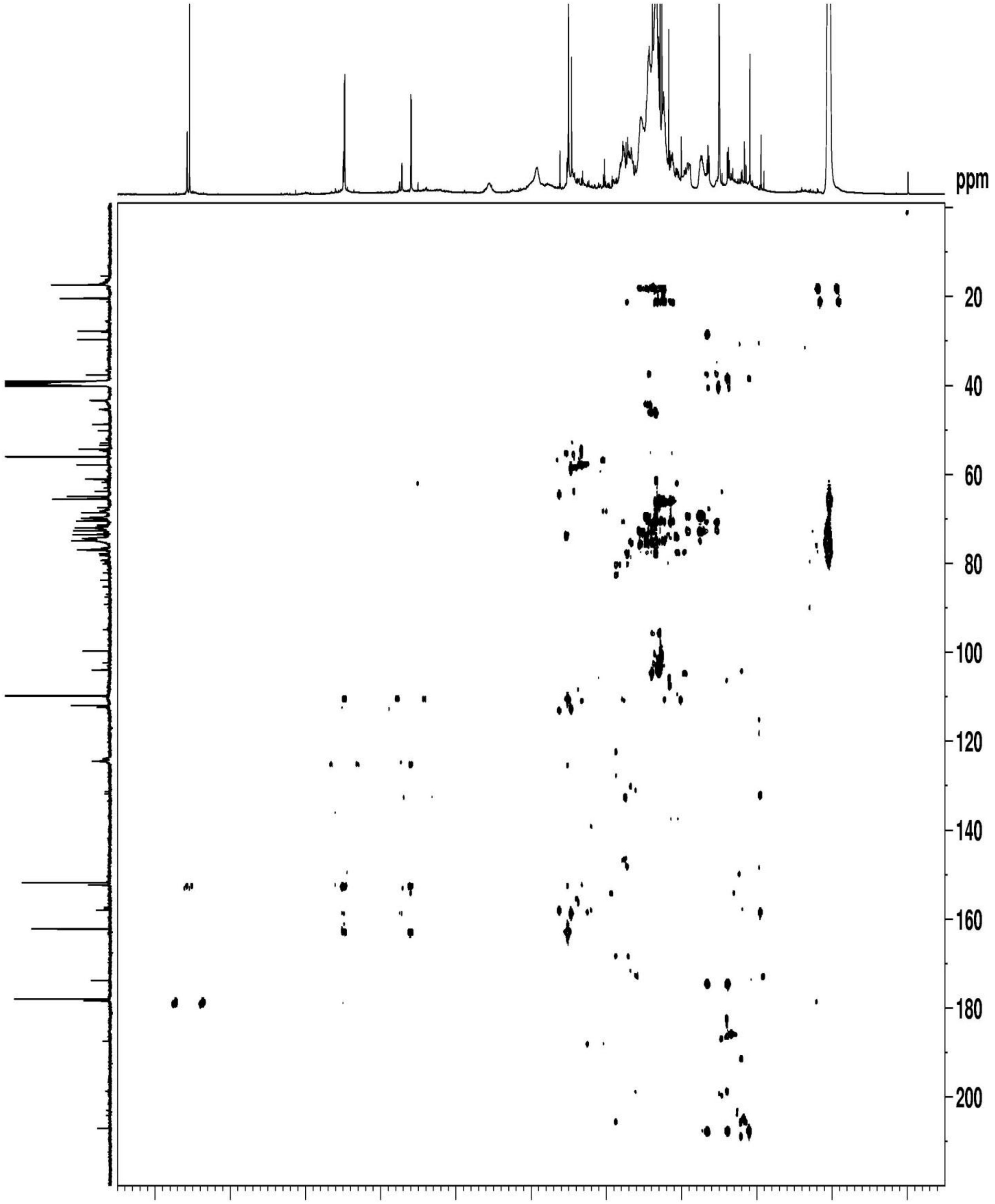

Figure S8f – HMBC spectrum for Capcure + Humins after 90 H at 353 K

IX - ELO + Humins after 90 H at 353 K

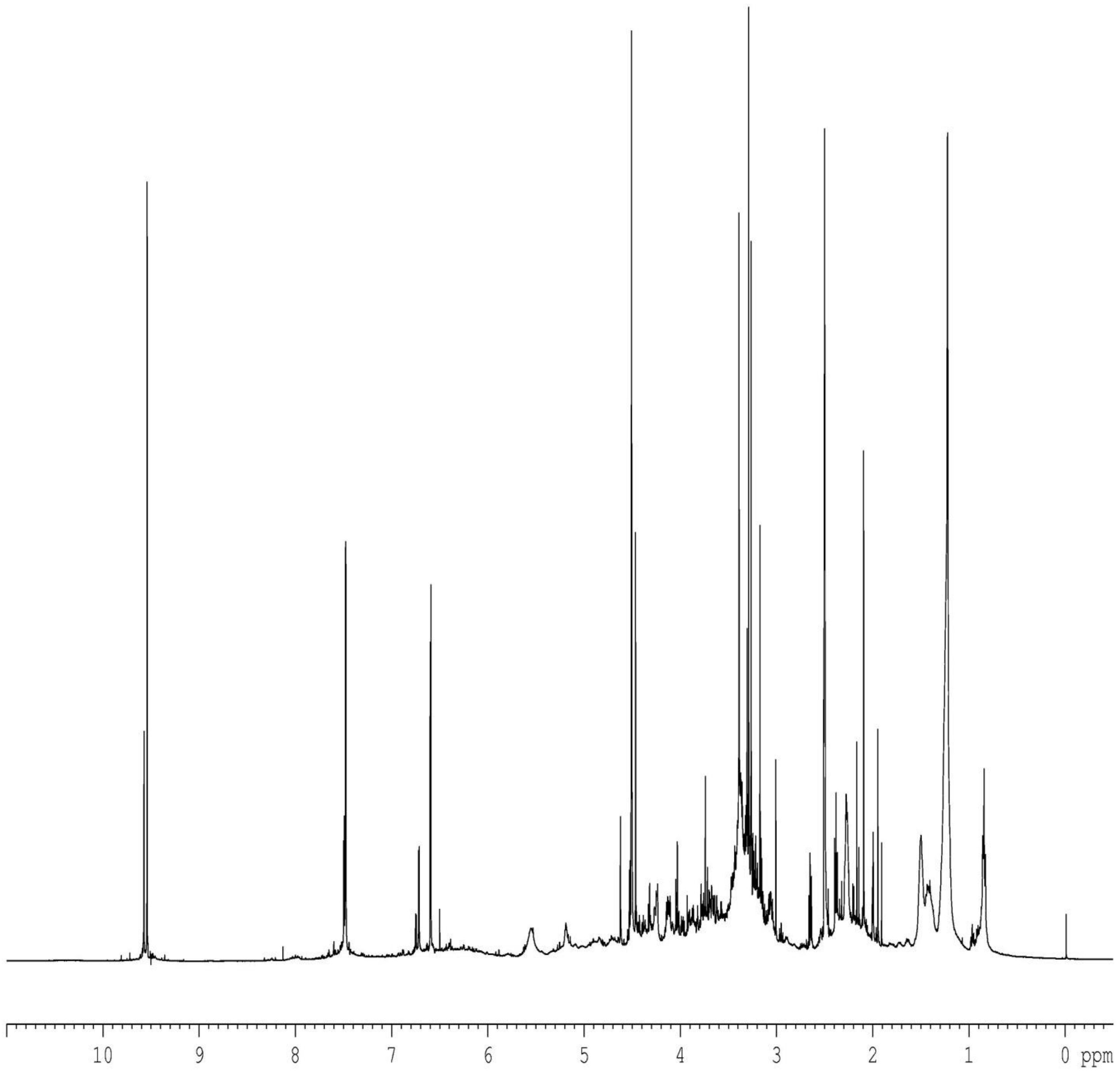

Figure S9a – ^{1}H-NMR spectrum for ELO+ Humins after 90 H at 353 K

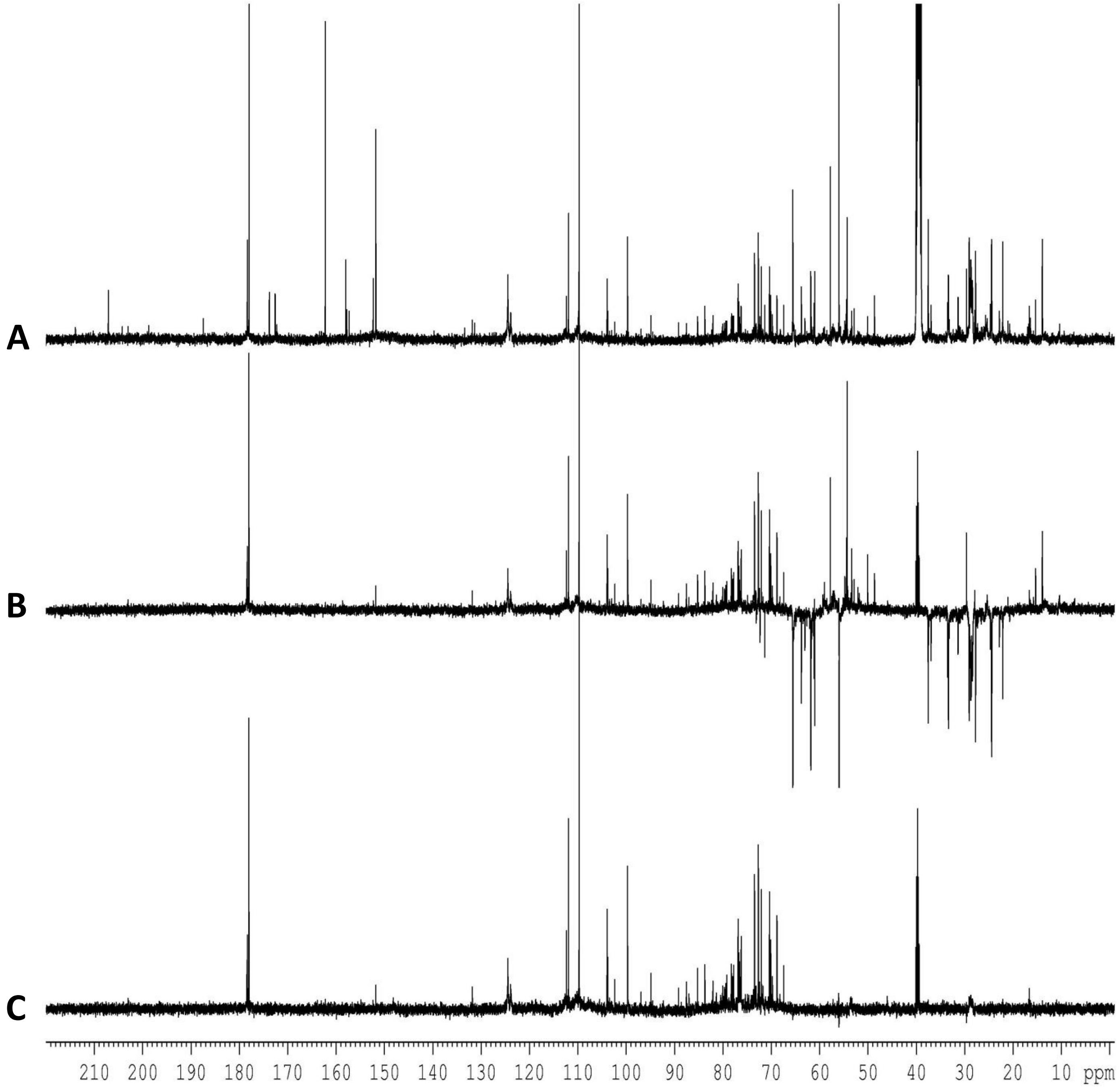

Figure S9b – ^{13}C-NMR spectrum for ELO + Humins after 90 H at 353 K - A : C13CPD - B : DEPT135 : C : DEPT90

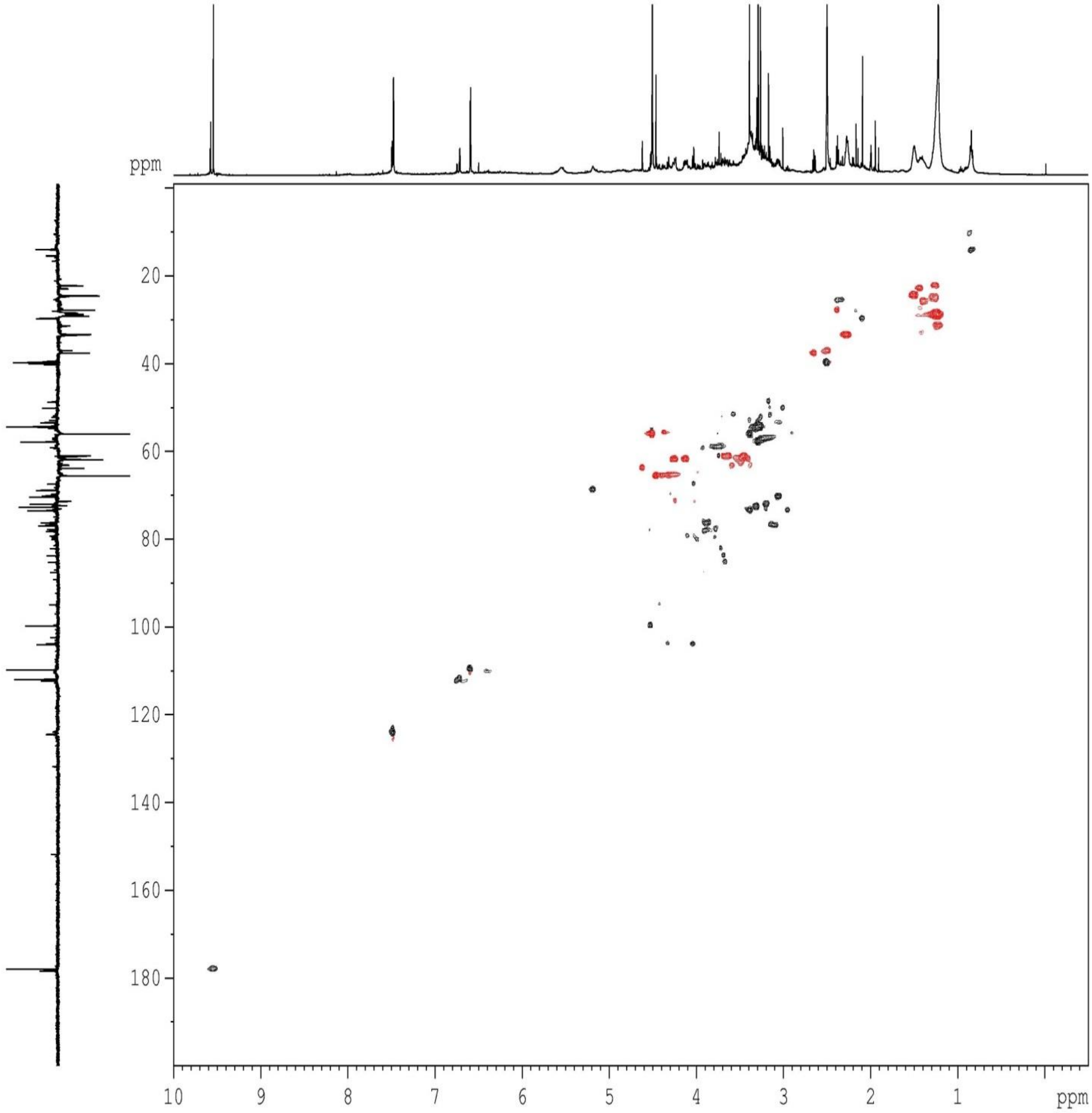

Figure S9c – HSQC spectrum for ELO+ Humins after 90 H at 353 K

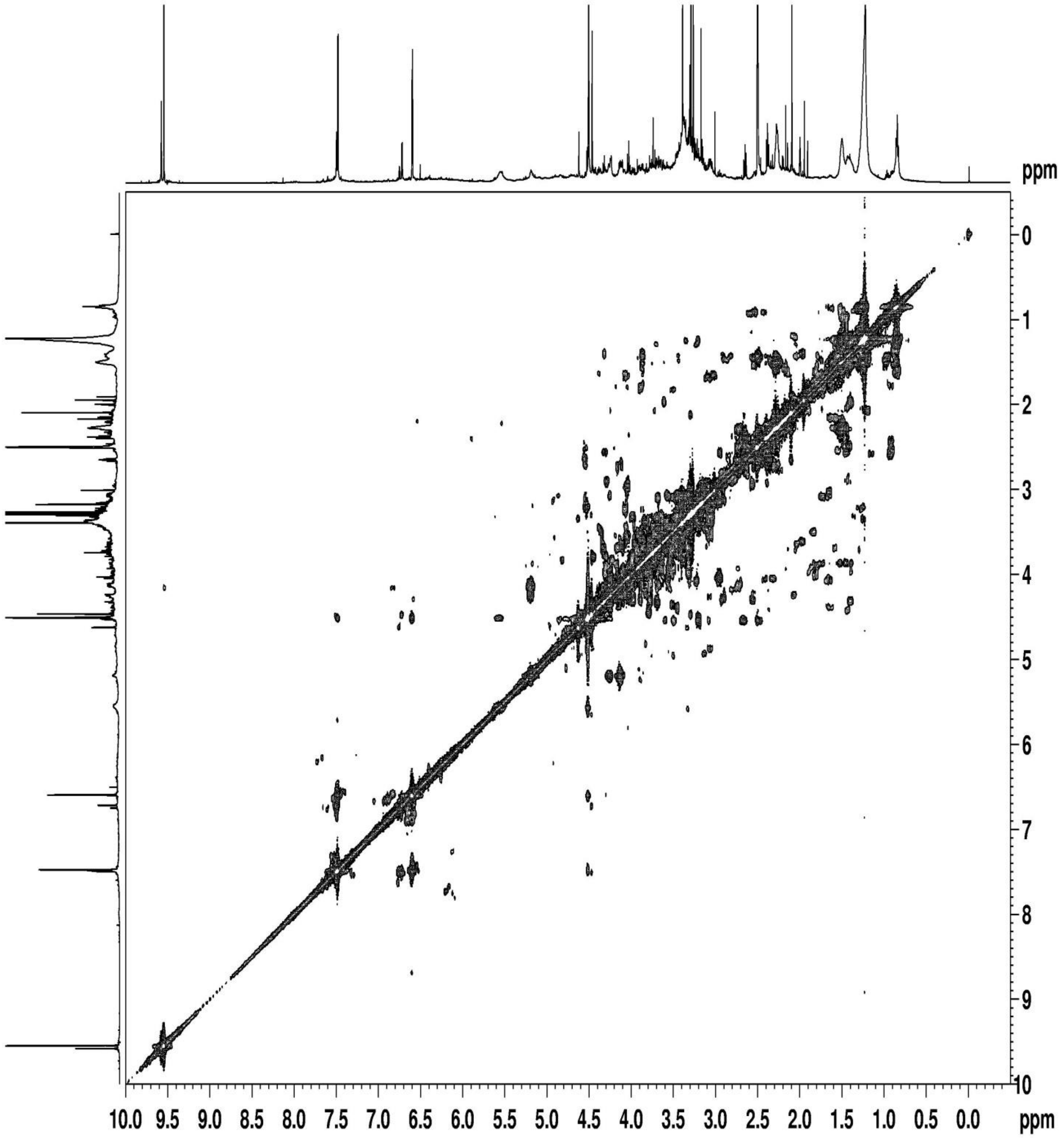

Figure S9d – COSY spectrum for ELO+ Humins after 90 H at 353 K

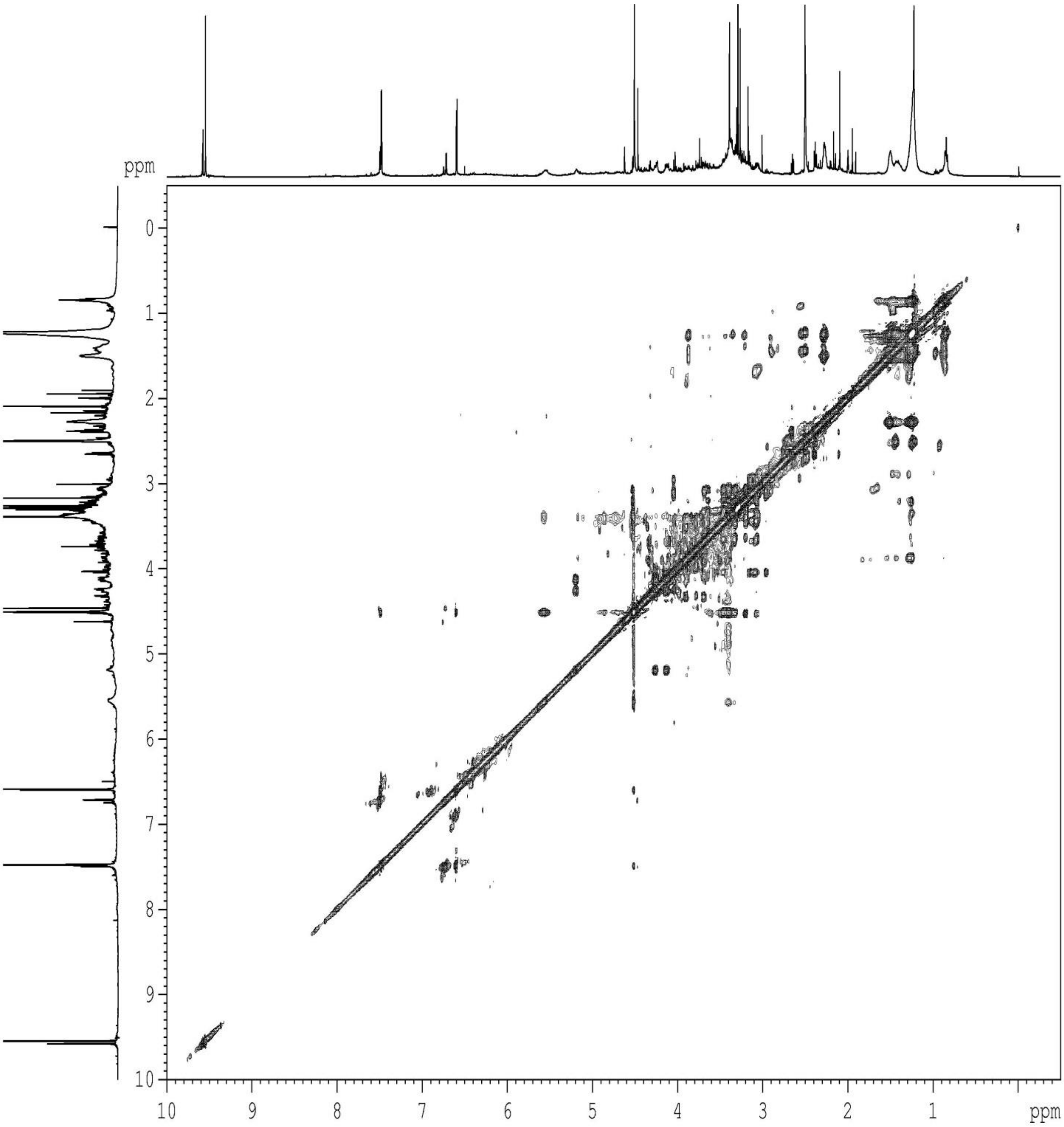

Figure S9e – TOCSY spectrum for ELO+ Humins after 90 H at 353 K

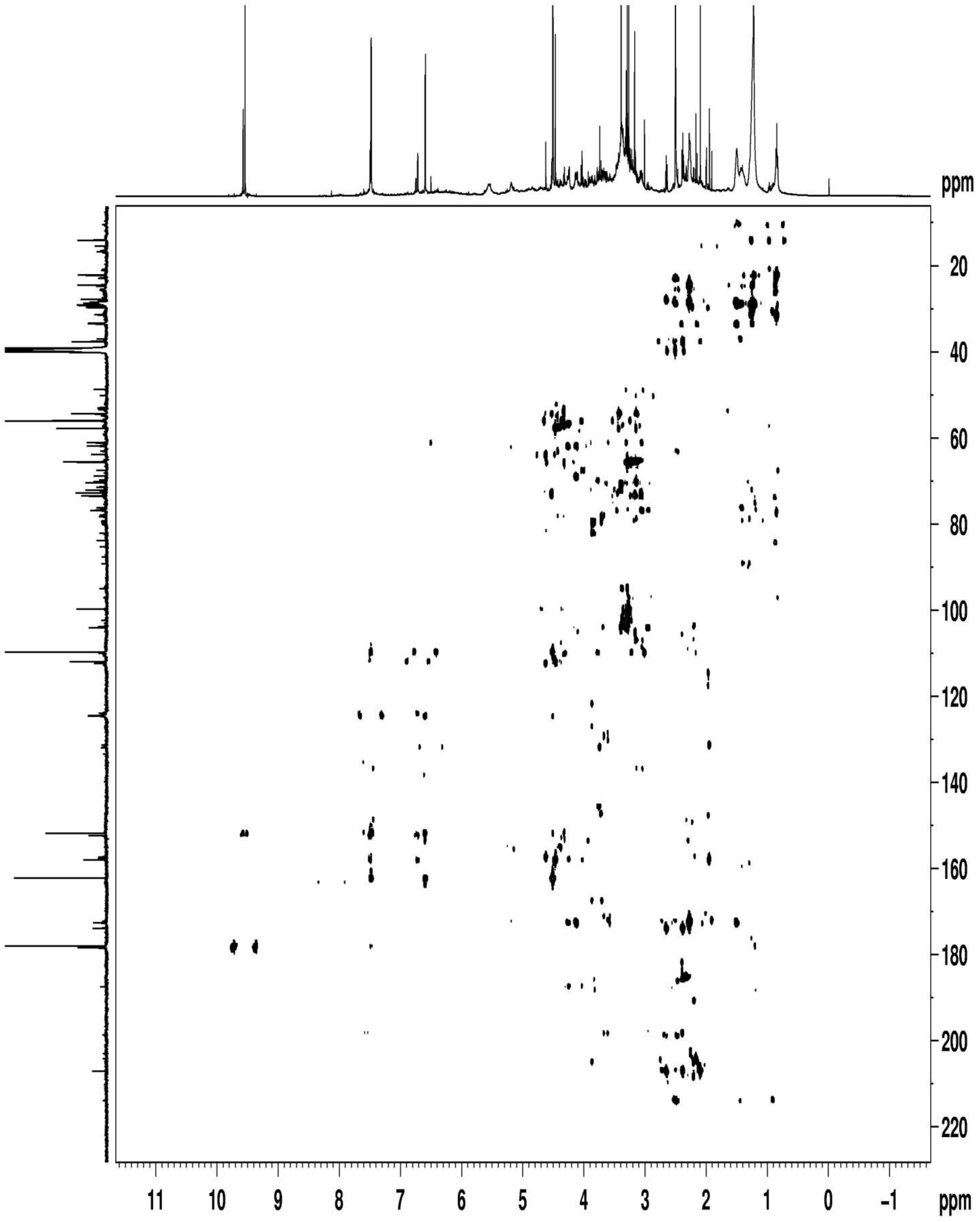

Figure S9f – HMBC spectrum for ELO+ Humins after 90 H at 353 K

X - ELO+ Capcure + Humins

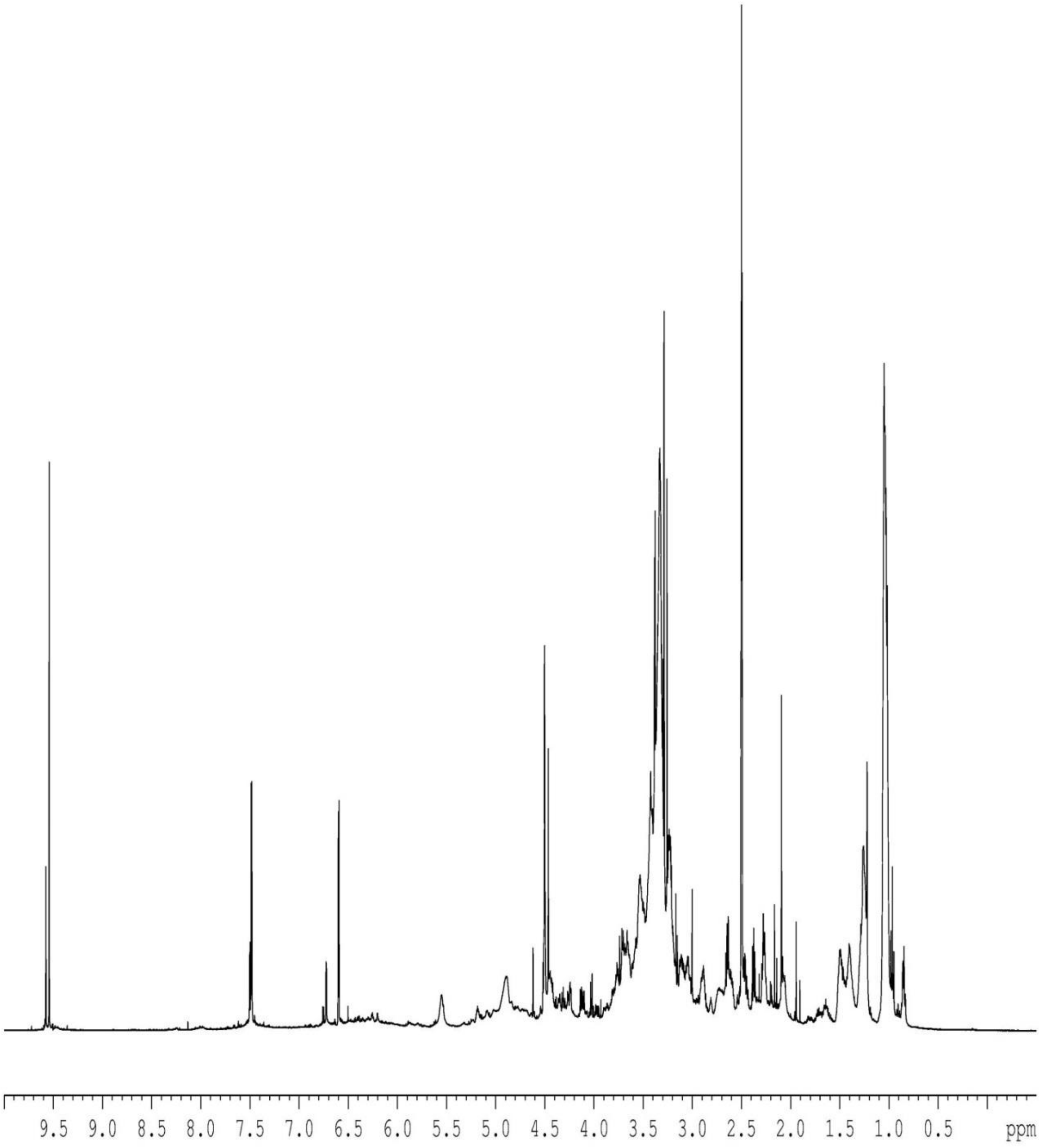

Figure S10a – ^{1}H-NMR spectrum for ELO+ Humins + Capcure

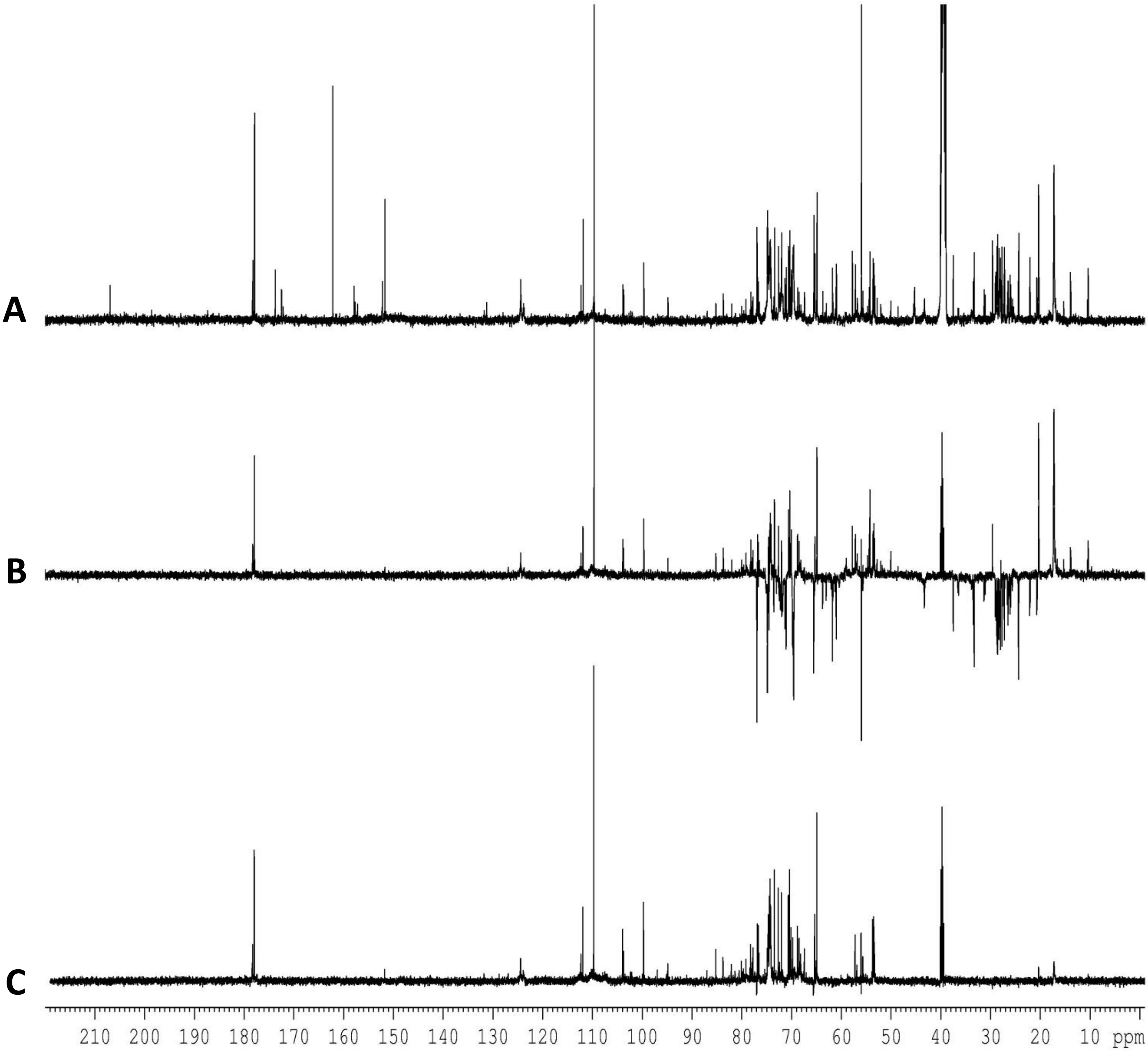

Figure S10b – ^{13}C-NMR spectrum for ELO + Humins + Capcure - A : C13CPD – B : DEPT135 : C : DEPT90

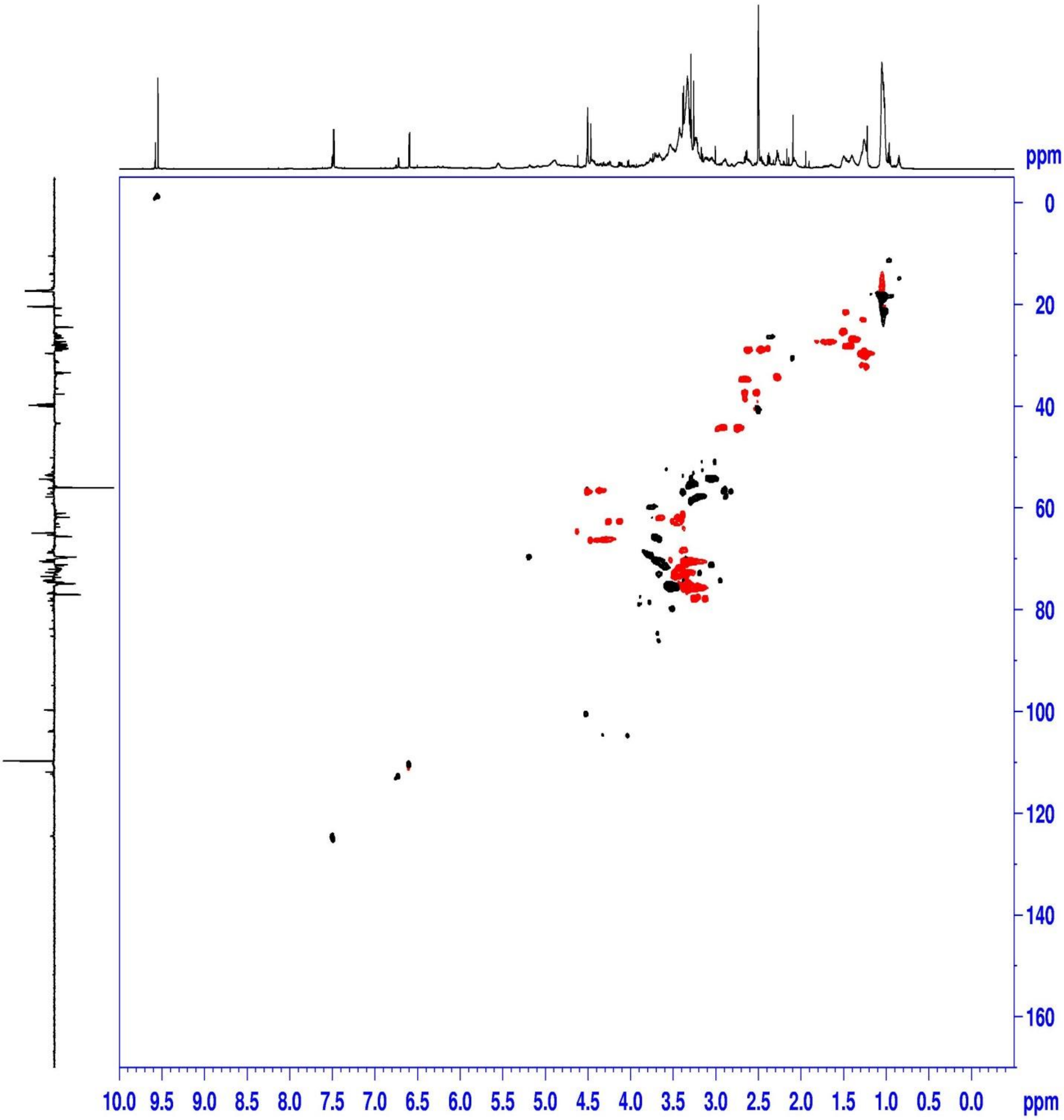

Figure S10c – HSQC spectrum for ELO+ Humins + Capcure

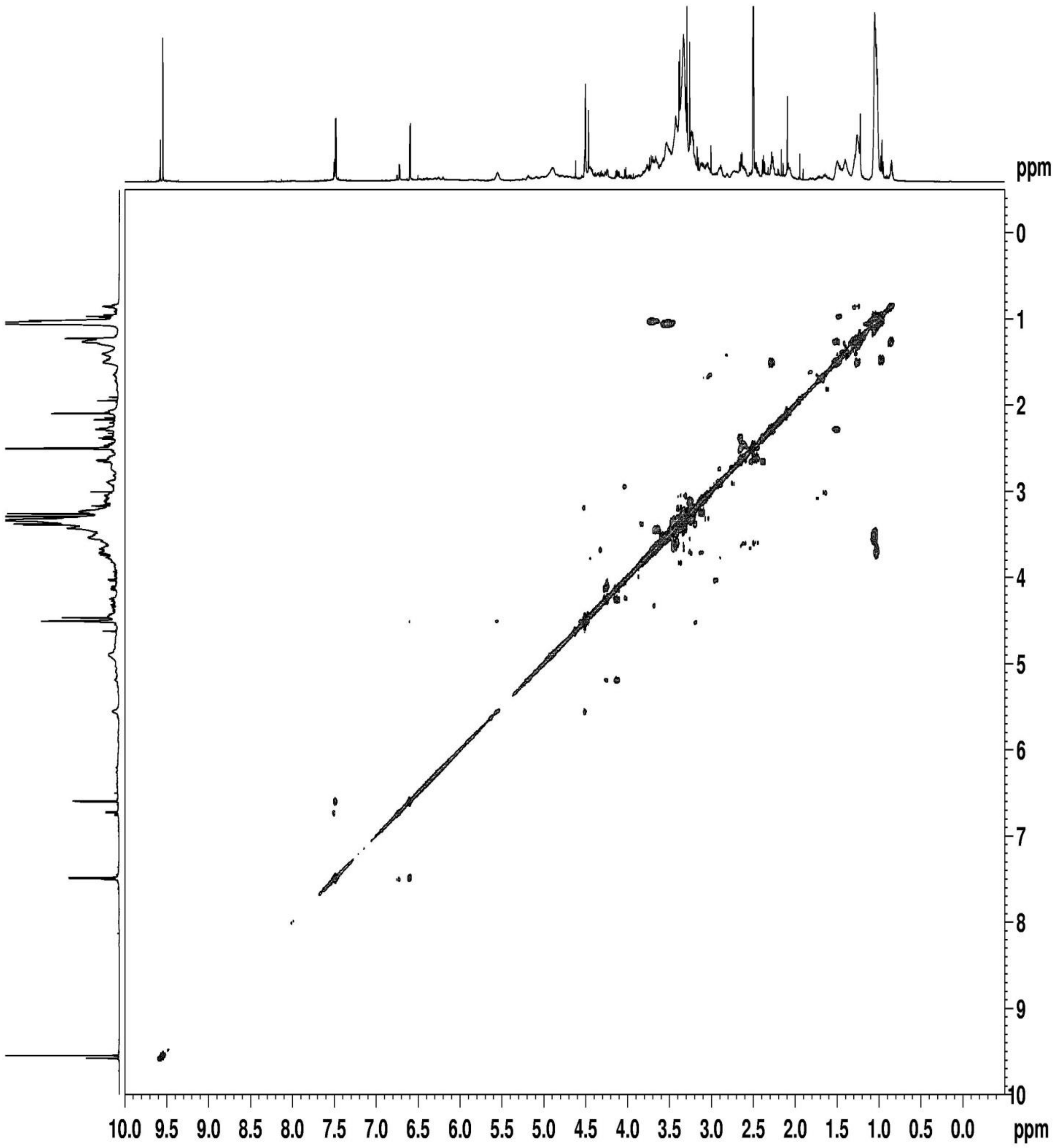

Figure S10d – COSY spectrum for ELO+ Humins + Capcure

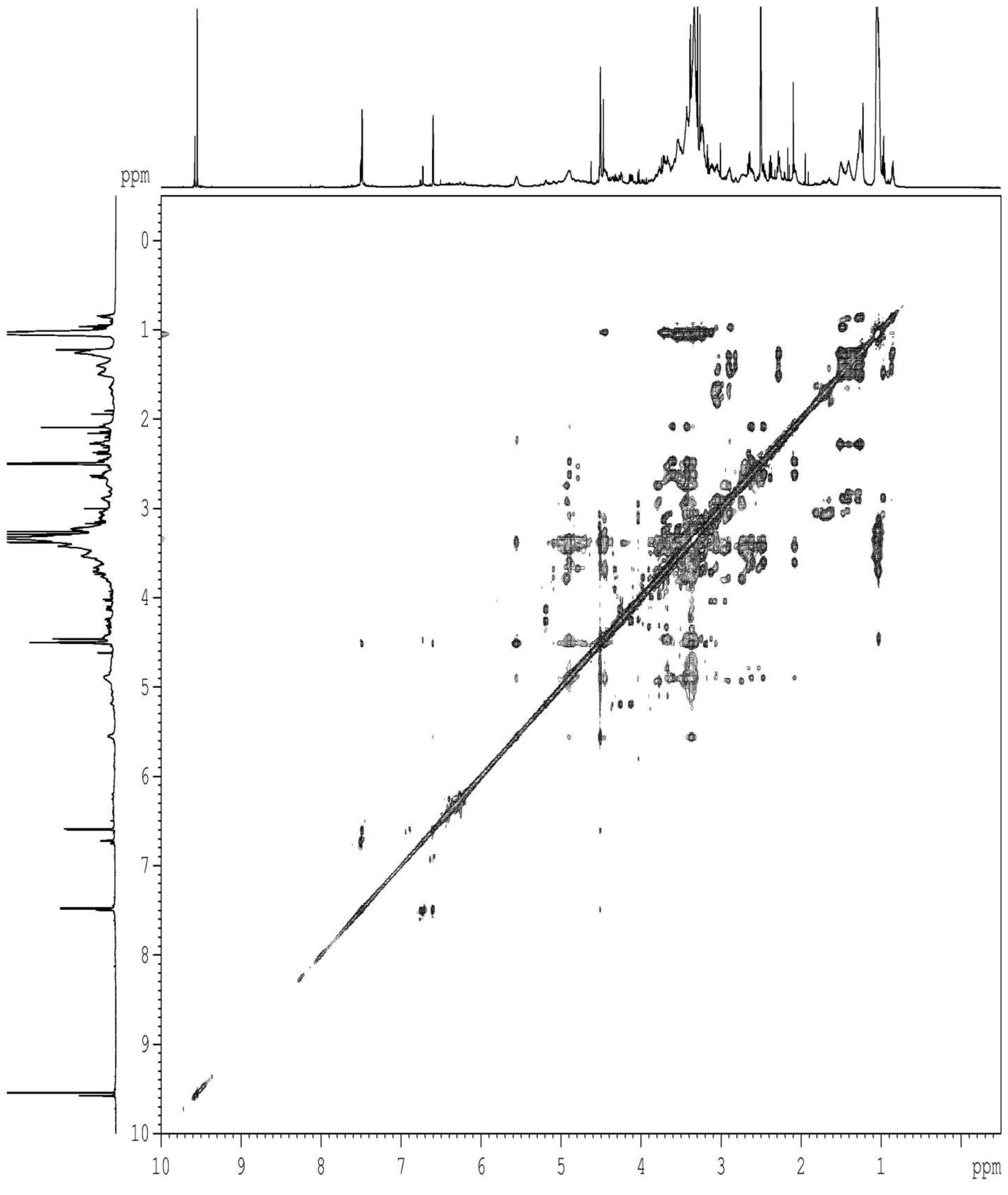

Figure S10e – TOCSY spectrum for ELO+ Humins + Capcure

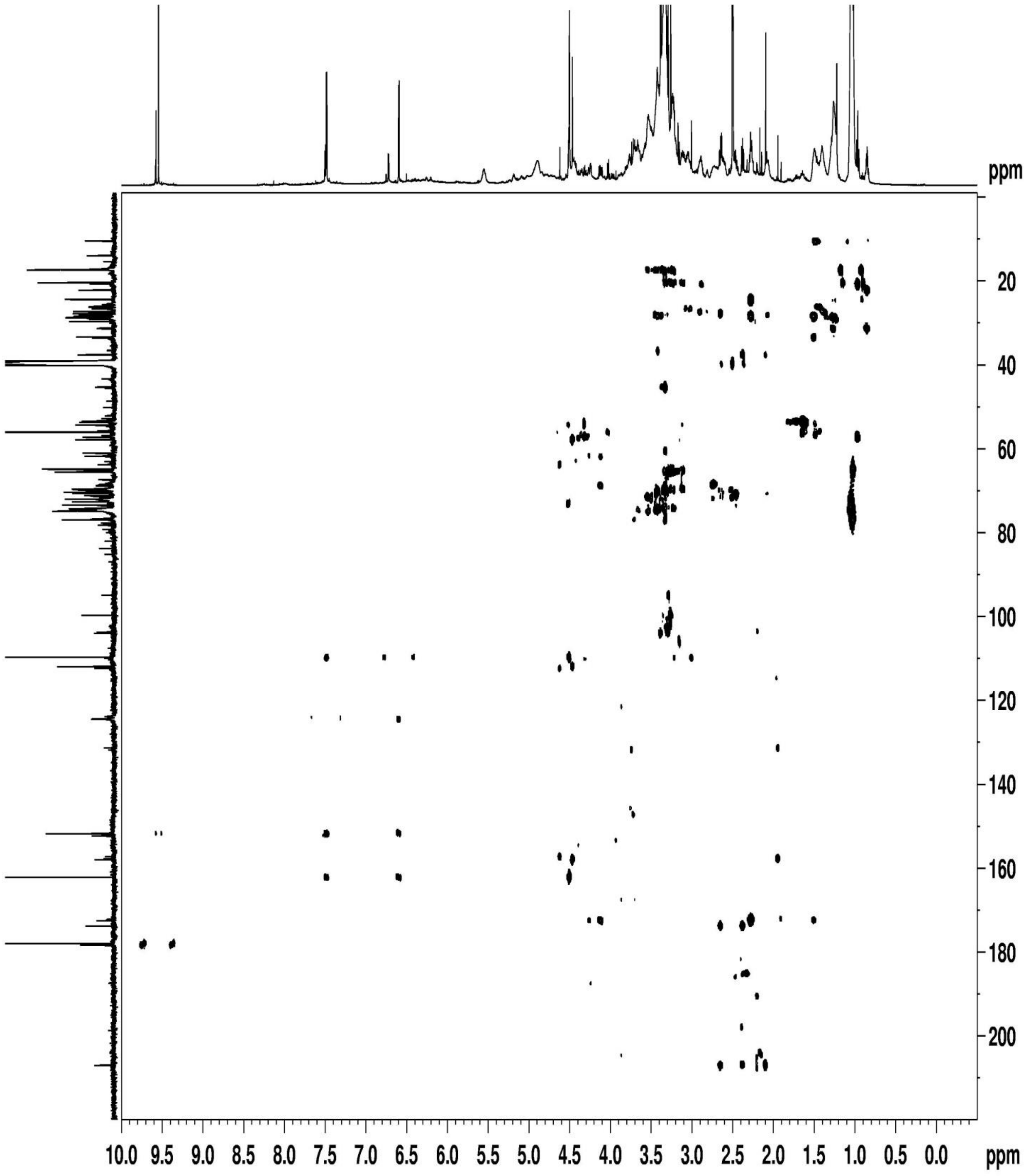

Figure S10f – HMBC spectrum for ELO+ Humins + Capcure

XI - ELO+ Capcure + Humins after 90 H at 353 K

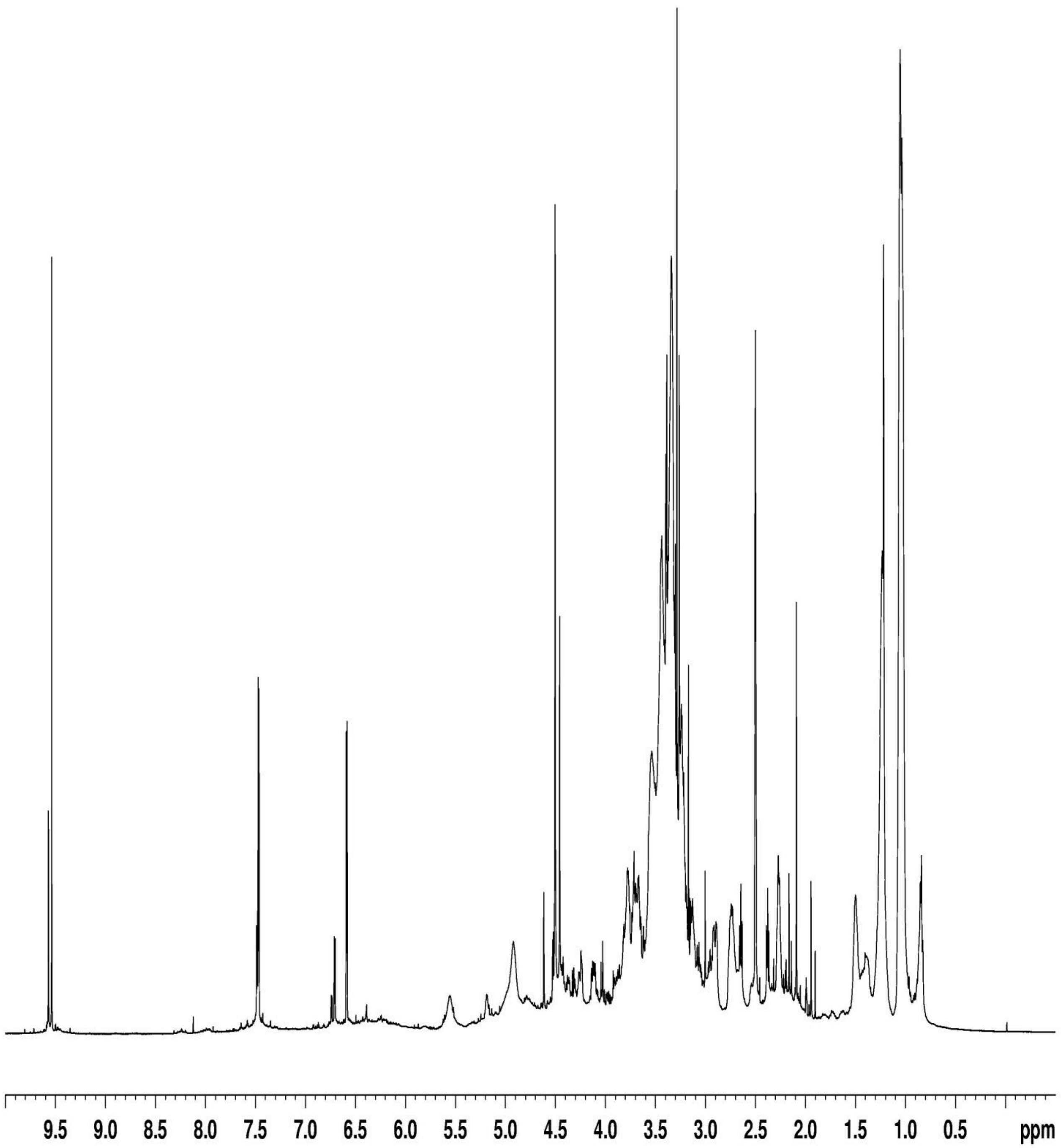

Figure S11a – ^{1}H-NMR spectrum for ELO+ Humins + Capcure after 90 H at 353 K

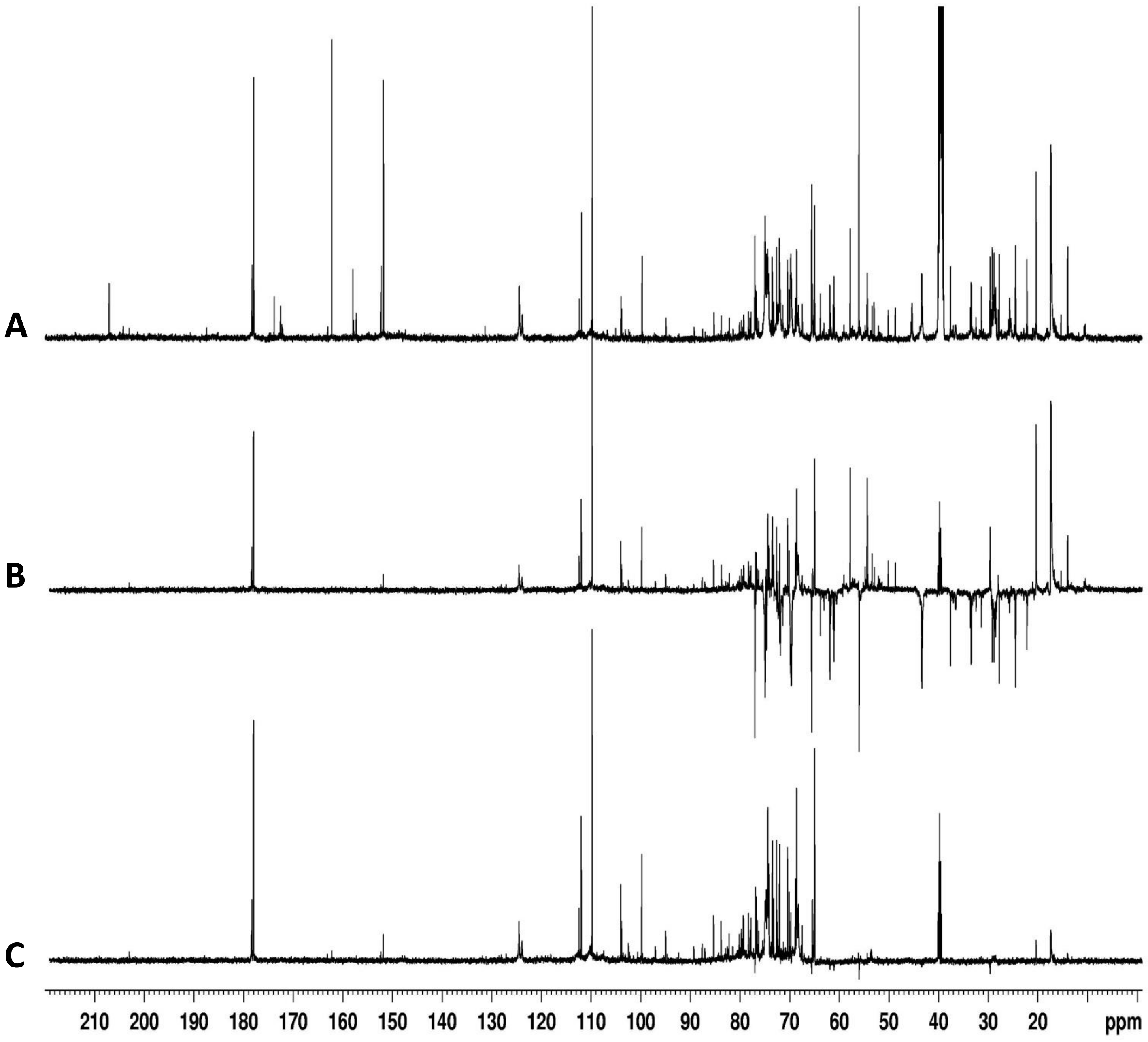

Figure S11b – ^{13}C-NMR spectrum for ELO + Humins + Capcure after 90 H at 353 K - A : C13CPD – B : DEPT135 : C : DEPT90

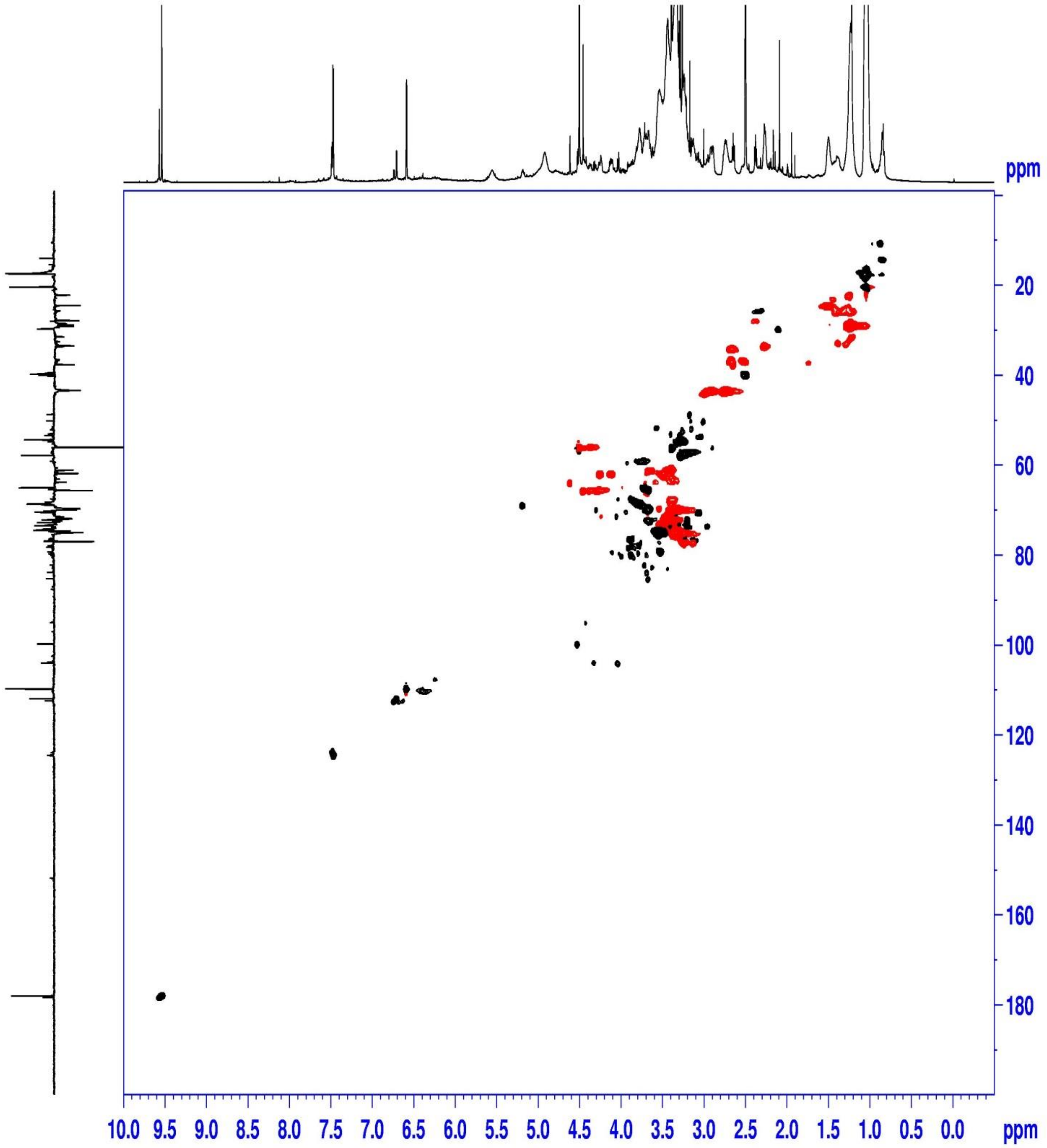

Figure S11c – HSQC-NMR spectrum for ELO+ Humins + Capcure after 90 H at 353 K

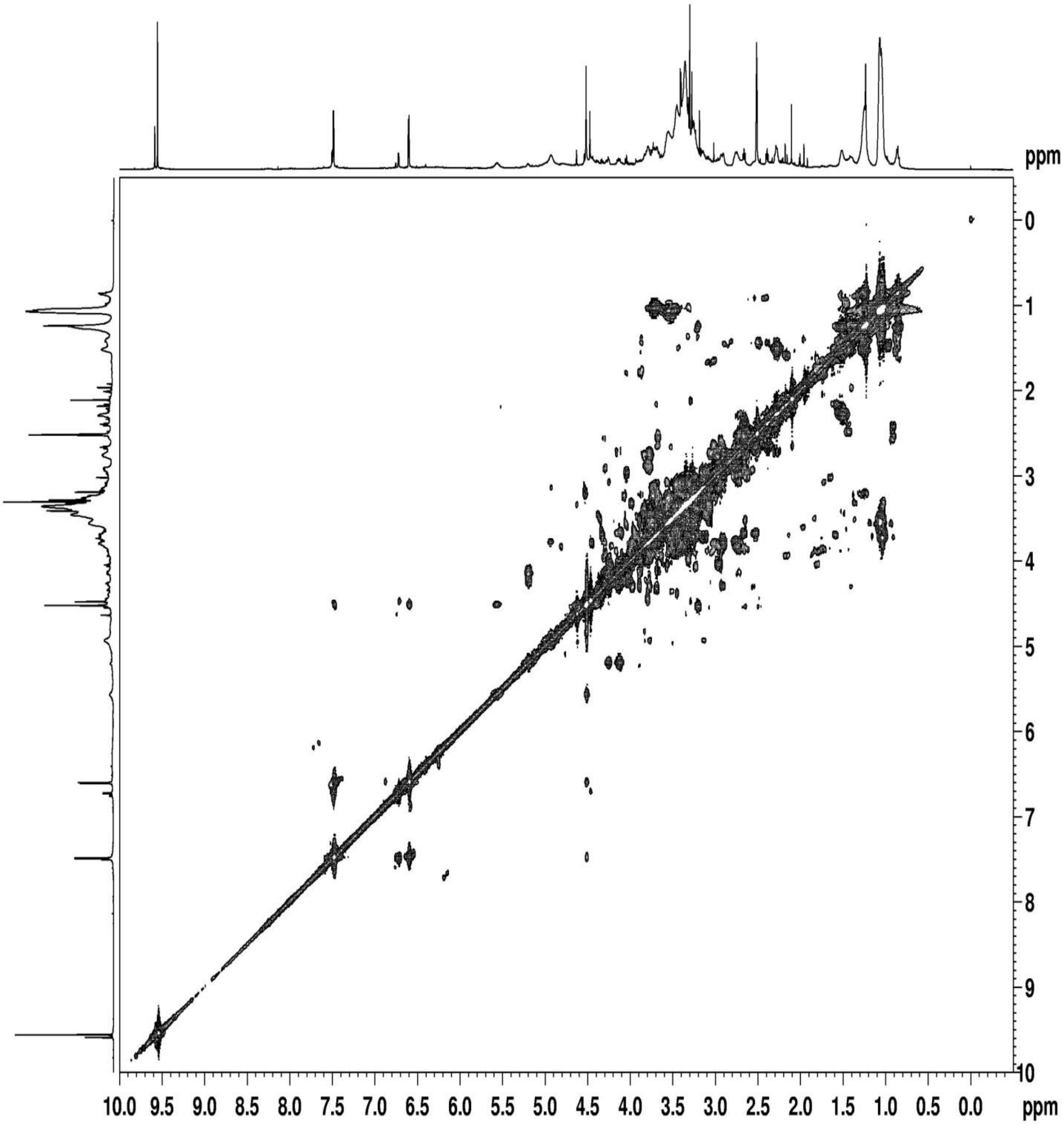

Figure S11d – COSY-NMR spectrum for ELO+ Humins + Capcure after 90 H at 353 K

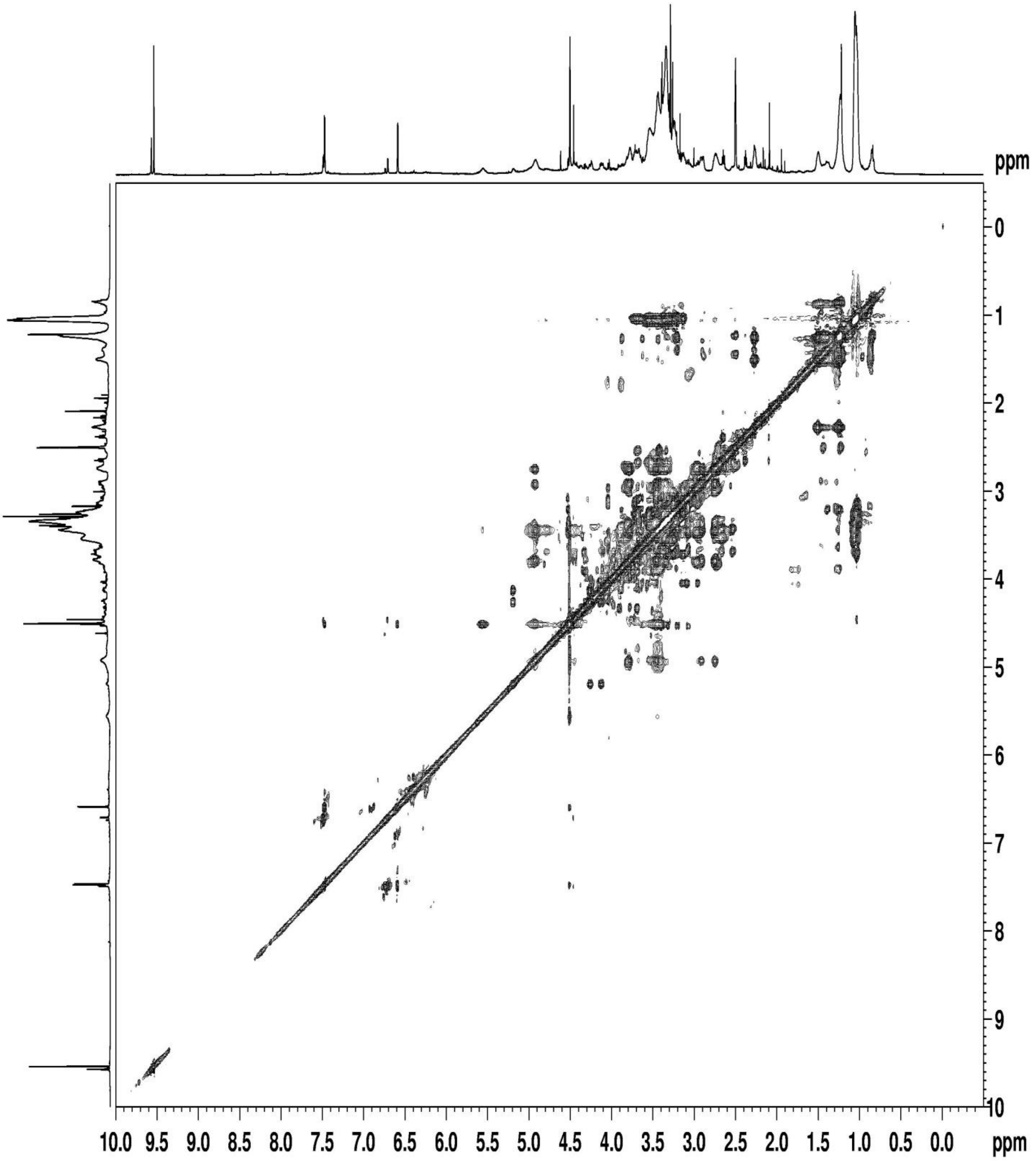

Figure S11e – TOCSY-NMR spectrum for ELO+ Humins + Capcure after 90 H at 353 K

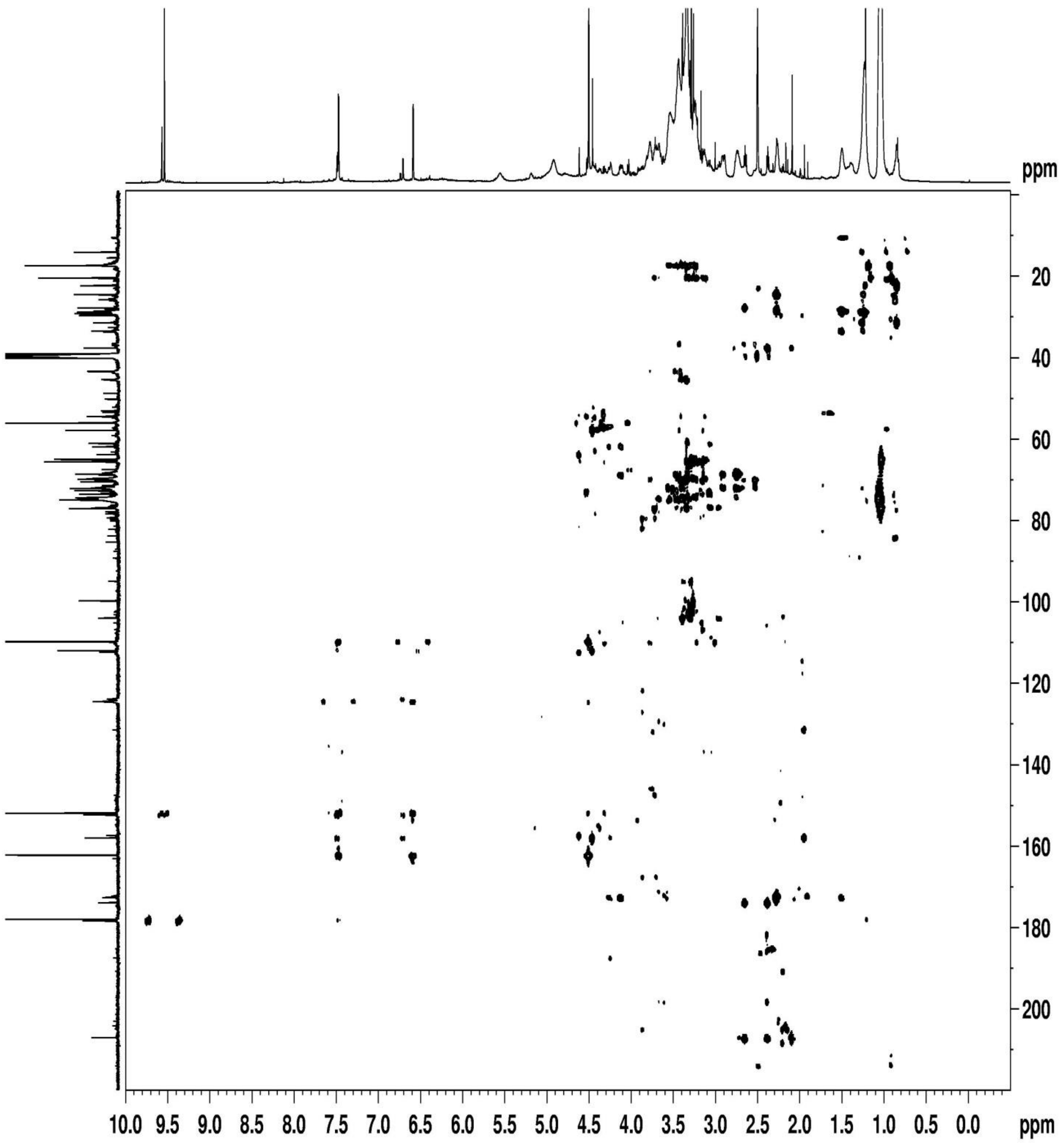

Figure S11f – HMBC-NMR spectrum for ELO+ Humins + Capcure after 90 H at 353 K

XII - ELO+ Capcure + Humins after 238 H at 353 K

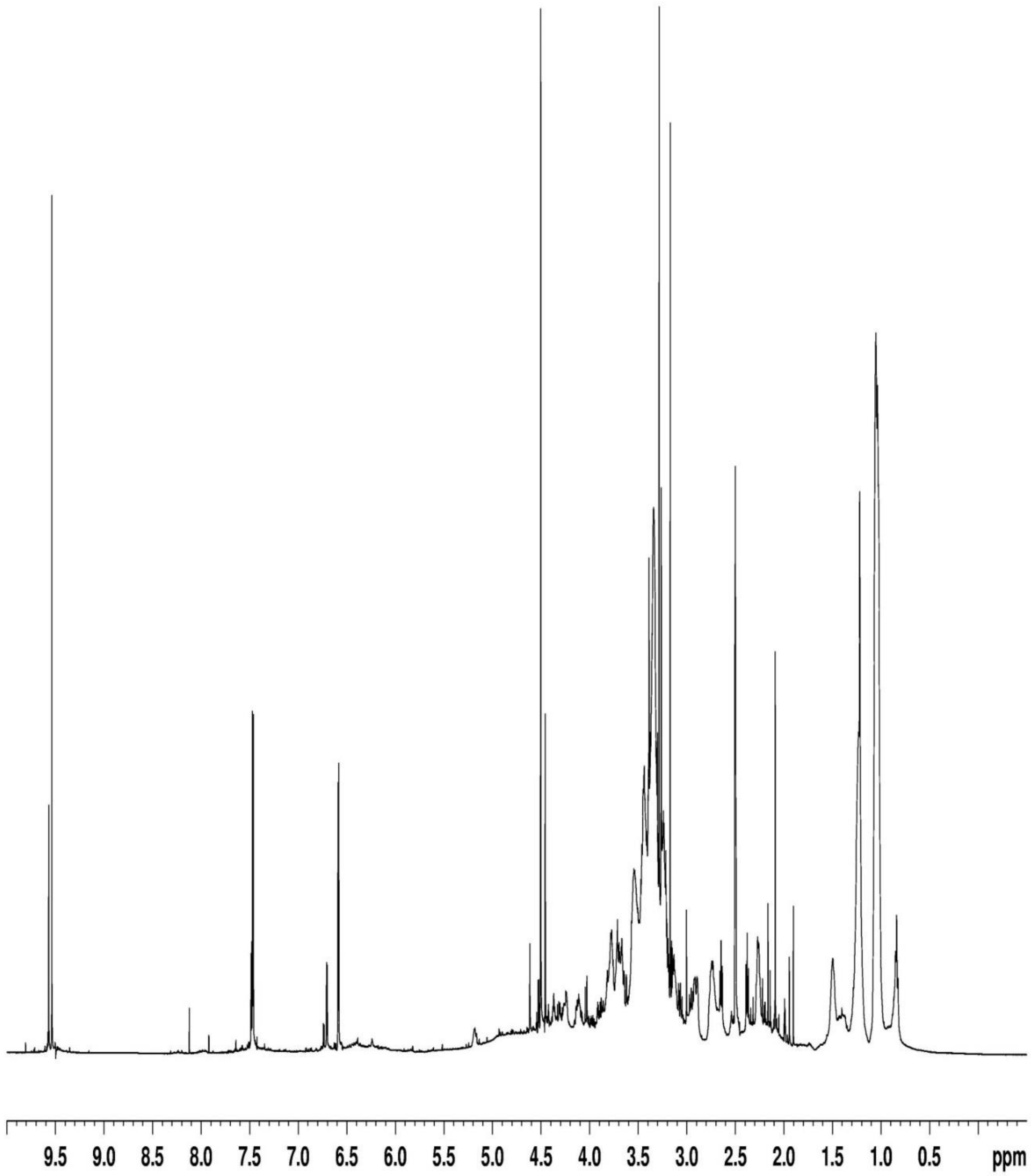

Figure S12a – ^{1}H-NMR spectrum for ELO+ Humins + Capcure **after 238 H at 353 K**

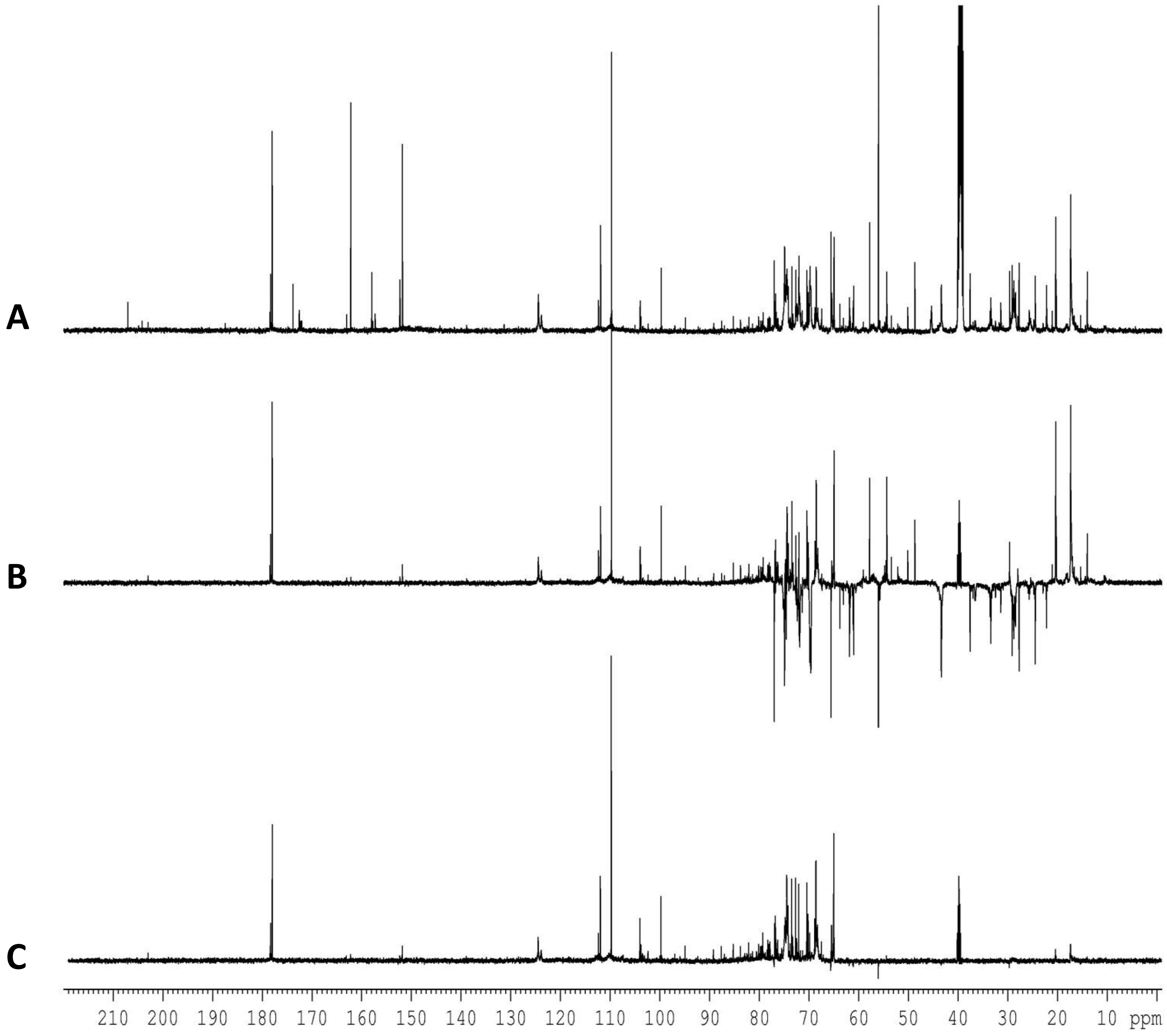

Figure S12b – ^{13}C-NMR spectrum for ELO + Humins + Capcure **after 238 H at 353 K -** A : C13CPD – B : DEPT135 : C : DEPT90

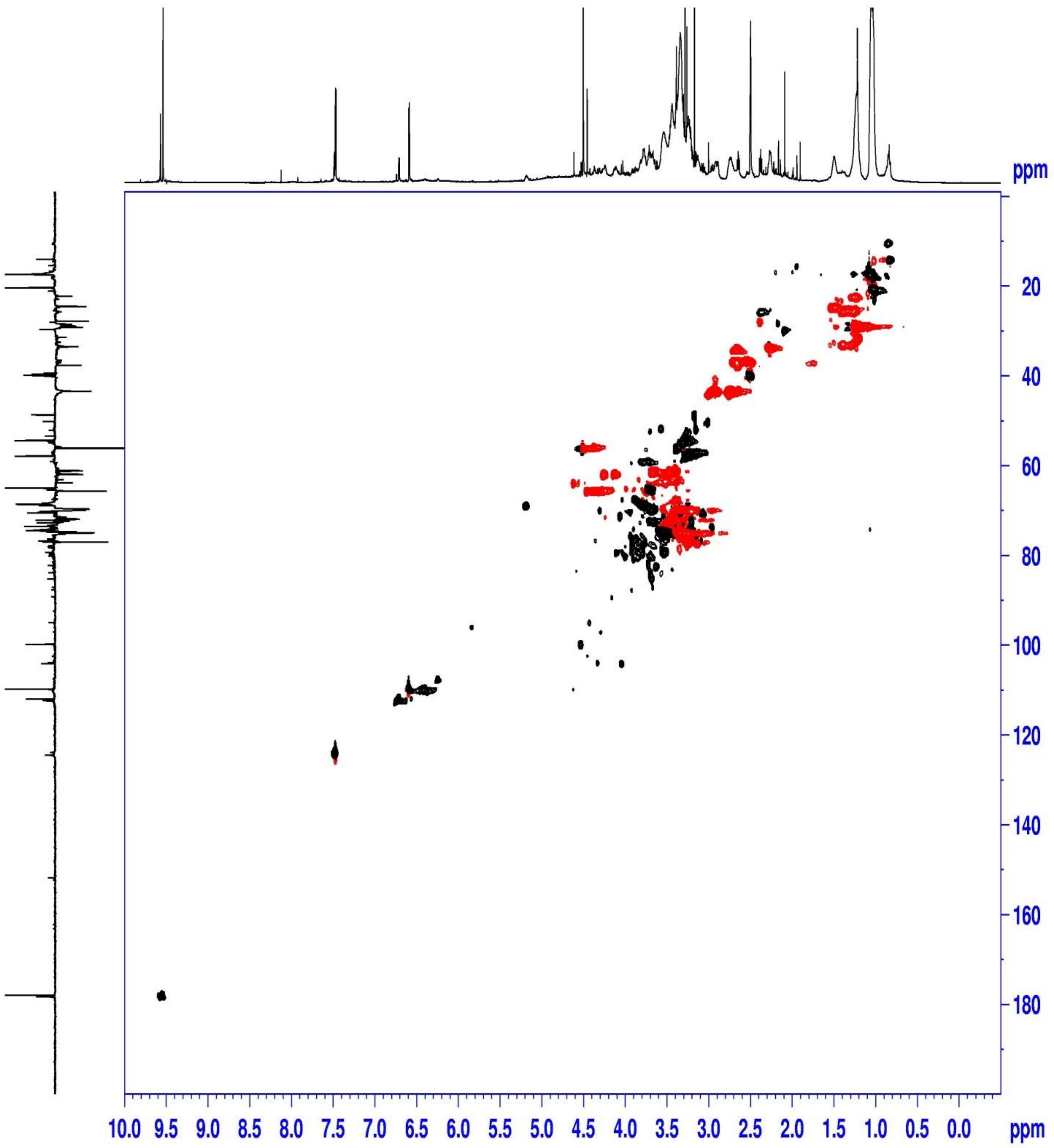

Figure S12c – HSQC-NMR spectrum for ELO+ Humins + Capcure **after 238 H at 353 K**

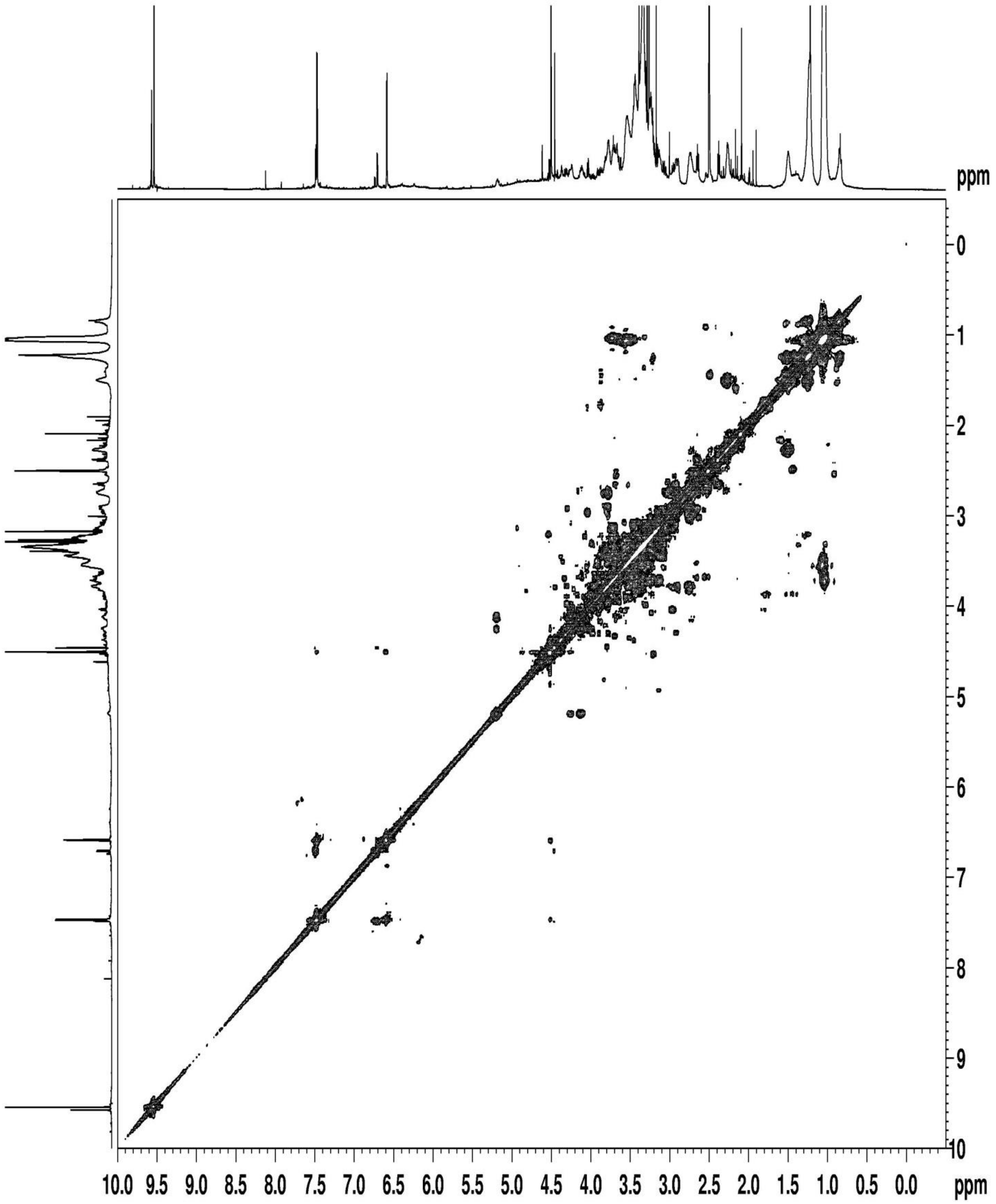

Figure S12d – COSY - NMR spectrum for ELO+ Humins + Capcure **after 238 H at 353 K**

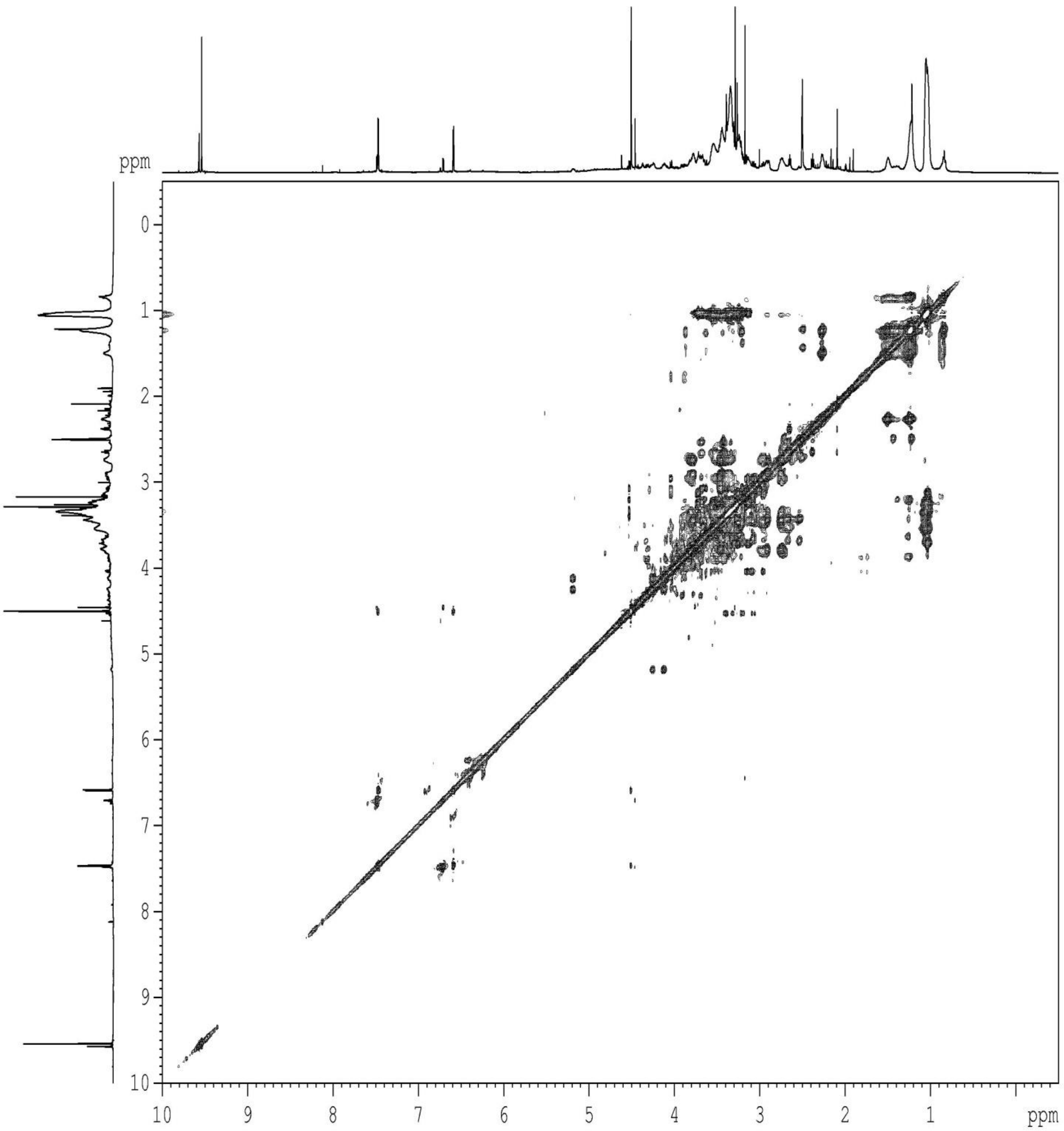

Figure S12e – TOCSY-NMR spectrum for ELO+ Humins + Capcure **after 238 H at 353 K**

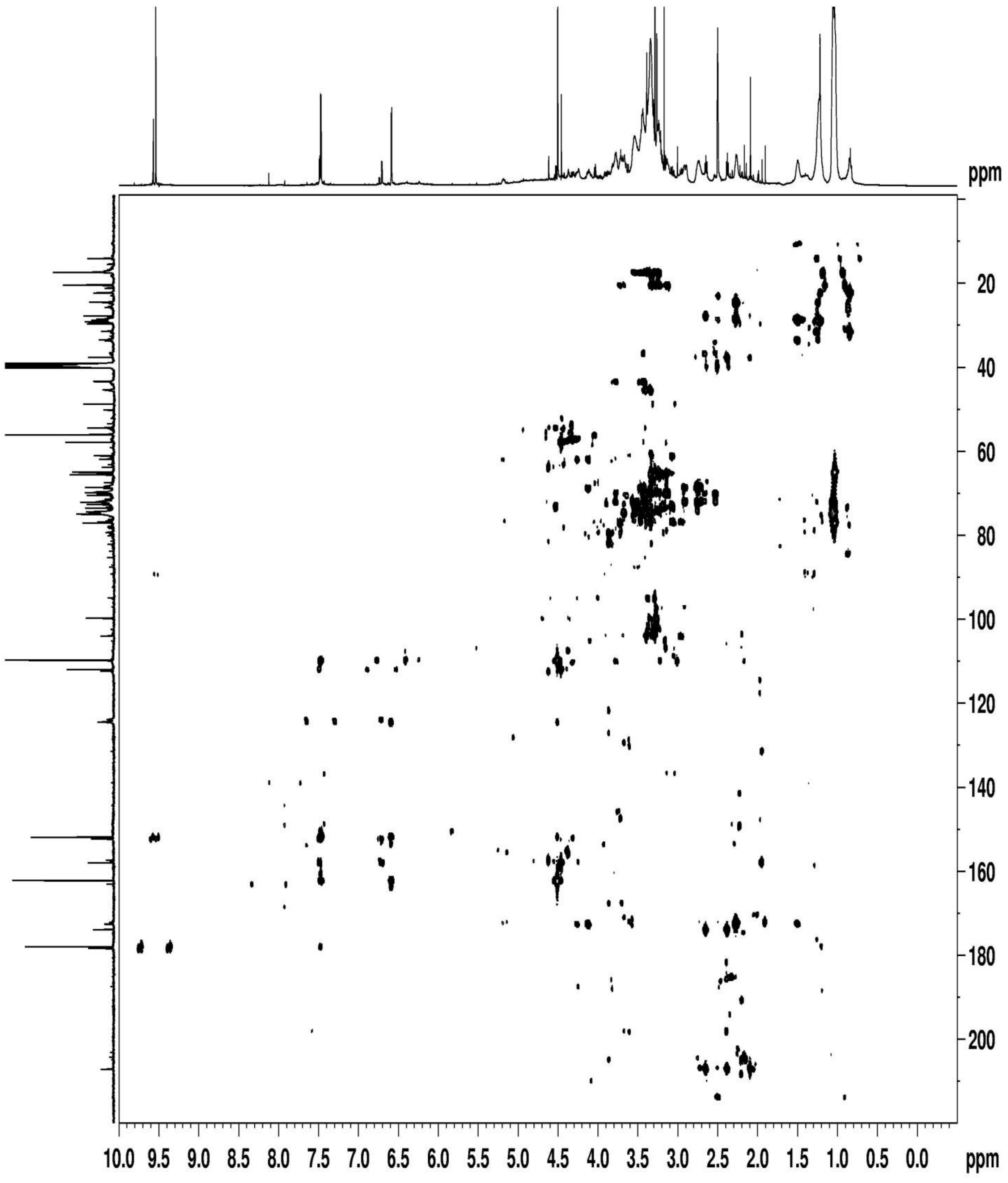

Figure S12f – HMBC-NMR spectrum for ELO+ Humins + Capcure **after 238 H at 353 K**
